# Health Risk Assessment for the Residential Area Adjacent to a Former Chemical Plant

**DOI:** 10.3390/ijerph19052590

**Published:** 2022-02-23

**Authors:** Eleonora Wcisło, Joachim Bronder

**Affiliations:** Institute for Ecology of Industrial Areas, 6 Kossutha St., 40-844 Katowice, Poland; j.bronder@ietu.pl

**Keywords:** soil pollution, human health risk assessment, risk-based remedial levels, chemical plant, residential area

## Abstract

A health risk assessment was carried out for the residents of Łęgnowo-Wieś settlement adjacent to a former Zachem Chemical Plant, Bydgoszcz, Poland. Due to the unique Zachem site history and contamination profile, an innovative strategy for soil sampling and contaminant selection was applied. The novelty in the developed strategy consisted of selecting substances for the health risk assessment, taking into consideration the location and boundaries of the groundwater contamination plumes in relation to contamination sources. This allowed limiting the number of the analysed contaminants. The risk assessment focused on the surface soil of a residential area, which was divided into 20 sampling sectors and 6 backyards with wells from which water was used for watering edible plants. A total of 80 inorganic and organic substances were determined, including metals, phenol, aniline, BTEX, diphenyl sulphone, chloroaniline, epichlorohydrin, hydroxybiphenyl, nitrobenzene, octylphenols, toluenediamine, toluidine, 16 polycyclic aromatic hydrocarbons, tetrachloroethylene and trichloroethylene. For the health risk assessment, the United States Environmental Protection Agency’s deterministic method was applied. This applies conservative assumptions to obtain risk estimates protective for most of the potential receptors. Three exposure pathways were analysed: (1) incidental soil ingestion, (2) dermal contact with soil and (3) inhalation of fugitive soil particles and volatiles. In all sampling sectors and backyards, the total non-cancer risks (hazard index) were significantly lower than the acceptable level of 1. The acceptable cancer risk level for the single carcinogen of 1 × 10^−5^ was only insignificantly exceeded in the case of benzo(a)pyrene in three sectors and one backyard. The total cancer risks were lower than the acceptable level of 1 × 10^−4^ in all sampling sectors and all backyards. The findings show that the soil in the entire residential area is safe for the residents’ health and no remedial actions are required. However, since not all possible exposure pathways were analysed in this study, further research focused on assessing the health risk resulting from the consumption of locally grown food is strongly recommended.

## 1. Introduction

There are millions of abandoned and still used contaminated sites around the world. It is estimated that only in the European Union (EU) there may be about 2.8 million contaminated sites, with more than 650,000 of them being registered in the national and regional inventories [1] (The problem of contaminated sites is particularly significant in the case of historical contamination and relates to abandoned sites. Such sites, if not remediated, may pose a health risk to current or future users and nearby residents, and, in particular, to the most sensitive groups, such as foetuses, children and pregnant women [2].

Soil contamination can be caused by various anthropogenic activities, such as industrial processes, mining, inappropriate waste management and treatment, agriculture, extraction and processing of fossil fuels or emissions from transport [2,3,4]. Soil contaminants can affect the air, surface and groundwater. They can also enter the food chain and cause diseases and mortality in living organisms, including humans [2,5,6,7]. The impact of soil contamination on human health has been studied for many years, particularly intensively in recent years. These studies have been conducted in areas of different anthropogenic activities, especially in urban and mining areas, in the vicinity of industrial areas, in the areas affected by warfare activities and agricultural areas [8,9,10,11]. In the assessment of the impact on human health, both inorganic and organic contaminants were taken into account. However, potentially toxic elements (arsenic and heavy metals) were assessed the most often [9,12,13,14,15,16,17,18]. Among organic compounds the studies often included polycyclic aromatic hydrocarbons (PAHs), [19,20,21,22,23,24,25], petroleum hydrocarbons [22,26,27] and pesticides [3,11,28,29].

In Europe, the problem of historically contaminated soil is caused by using dangerous substances in many production processes and the lack of legally imposed emission control or protective measures. This problem is visible especially in Eastern and Central Europe, including Poland [1]. 

The economic changes taking place in the nineties of the 20th century caused liquidation of industrial plants located in cities, which resulted in a number of abandoned, heavily contaminated sites. The complex environmental problems they cause and their scale allow calling these sites “megasites“ or even “environmental bombs“. In Poland alone there are several such sites, for example: Tarnowskie Góry Chemical Plant [30], Organika-Azot S.A. Chemical Plant in Jaworzno [31] and Zachem Chemical Plant in Bydgoszcz [32].

As for the abandoned Zachem Chemical Plant (CP), the area was partially remediated and re-used as a technological park. However, the impact of this site on the health of people who live in the vicinity of this plant has not been assessed, causing concern for them and their children.

The aim of the study was to assess the health risk posed to the residents of the Łęgnowo-Wieś settlement (ŁWS) situated in the vicinity of the Zachem CP. It was intended to be achieved through:

▪ development of an innovative strategy for soil sampling and contaminant selection, taking into consideration the location and boundaries of the groundwater contamination plumes in relation to contamination sources;
▪ selecting substances for health risk assessment according to the developed strategy;
▪ soil sampling and chemical analyses;
▪ assessing the health risk to the residents of ŁWS as a result of the potential exposure to the selected contaminants;
▪ developing the site-specific health risk-based target levels (HRBTLs) for the selected contaminants.

## 2. Materials and Methods

### 2.1. Site Area

**Figure 1:**
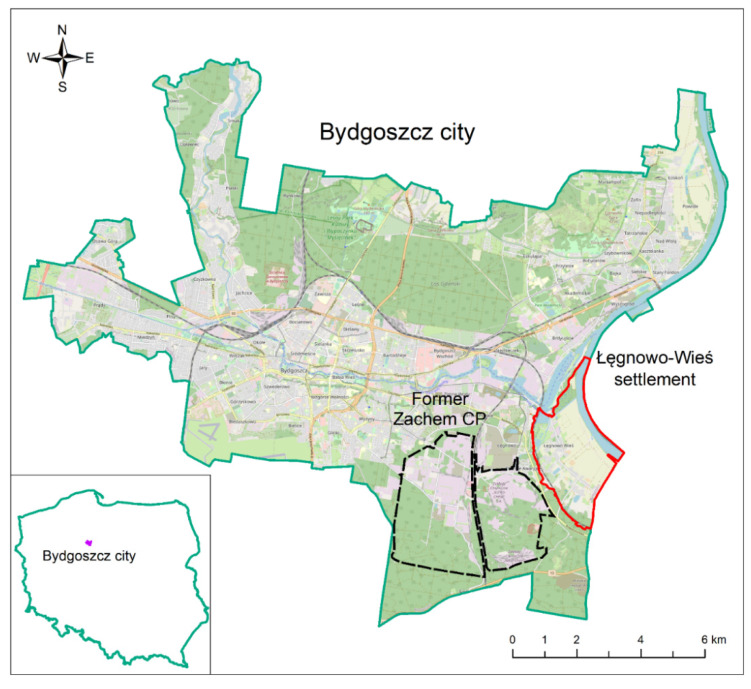
Location of the former Zachem Chemical Plant and the Łęgnowo-Wieś settlement on the map of Bydgoszcz, Poland.

The former Zachem CP is located in the city of Bydgoszcz (53°07′24″ N; 18°00′27″ E), in the northern part of Poland. The site is situated in the south-eastern part of the city, at a distance of about 7 km from the city centre (Figure 1). Its area covers ca. 2000 ha.

The Zachem CP was established in the place of the former German explosives factory built during the Second World War [33]. The main products of the factory were: nitrocellulose, smokeless gunpowder and nitroglycerine, trinitrotoluene—TNT (trotyl), dinitrobenzene, V1 missiles, aircraft bombs, artillery shells and gunpowder fuses [34]. In 1948–1952, the plant produced mainly explosives, such as trotyl, penthrite and tetryl for military and civilian needs. Acid denitration and nitrating acid management were also conducted. Moreover, dyes, dyeing intermediates, pigments, phenol, optical brighteners, nitrobenzene, aniline and the products made from processed poly(vinyl chloride) (PVC) were produced. In the 70s, the production of flexible polyurethane (PU) foams and fittings from PU foams for the automotive industry was started and installations for the production of phosgene, dinitrotoluene (DNT), toluenediamine (TDA), toluene diisocyanate (TDI), epichlorohydrin (EPI) and electrolysis of brine were launched [33].

In 1981–1990, the basic production of the Zachem CP was continued, but later on there was no further development due to economic problems in Poland. In the 90s, the eco-restructuring of the production was undertaken, which resulted in the closure of the most harmful installations. Since that time the plant has been reorganised, modernised and its production profile has altered. In the first decade of the 2000s, the most important production profile included: products made from processed PVC, PU foams, EPI and TDI [33]. In 2012, the production of the plant ceased. In 2014, the company was declared bankrupt, and since then its assets have been managed by a trustee [35].

The Łęgnowo-Wieś settlement (ŁWS) is located at the south-eastern side of the former Zachem CP (Figure 1). It covers the area of 736 ha and has ca. 1600 inhabitants. People live mainly in detached houses with backyards. In the eastern part of the settlement an agricultural area of 378.5 ha with a high groundwater level is located. Since 1963, residents of the ŁWS have reported the deterioration of water quality in private domestic wells. Zachem CP started providing drinking water to them from its own water intake [35]. Since 2014, drinking water has been provided for residents from the municipal water mains. However, some domestic wells are still used for watering edible plants grown in home gardens.

**Figure 2:**
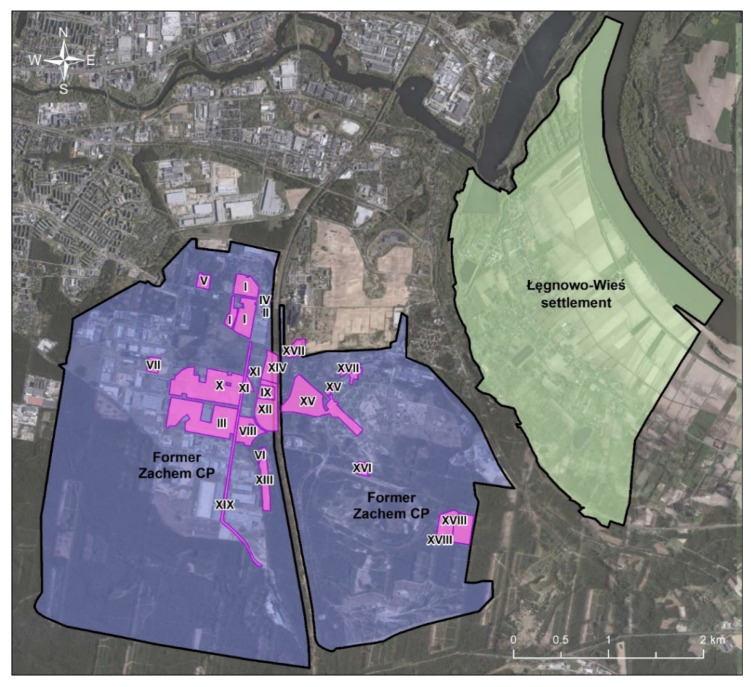
Location of the main contamination sources in the former Zachem CP area by BRDEP, based on Czop et al. [37]; I: dye plant, II: dye waste pit, III: monomer complex installation, IV: cold control unit, V: manufacturing of polyester polyols and polyurethane systems, VI: TDI tar extinguishing site (former electroplating waste landfill), VII: brine electrolysis area, brine reservoirs, VIII: propylene warehouse, IX: heat and power plant ash and slag landfill, X: TDI/TDA contaminated area, XI: EPI installation, XII: EPI sedimentary pond, XIII: Lisia landfill, XIV: central sewage neutralisation station, XV: three aniline sludge storages (pits), XVI: DNT installation, XVII: old boiler house, XVIII: Zielona landfill complex, XIX: brine pipeline route.

In the previous decade, detailed exploratory studies in the Zachem CP area were conducted, mainly by AGH University of Science and Technology, Cracow and the Bydgoszcz Regional Directorate for Environmental Protection (BRDEP), [36,37]. AGH and BRDEP delineated 17 and 19 main contamination sources in the Zachem CP area, respectively (Figure 2). The studies pointed out the following most dangerous contamination sources: Zielona landfill (tens of thousands of tons of post-phenol sodium sulphite accumulated on 11 ha area), Lisia landfill (sodium sulphite contaminated with phenol), the former EPI sediment pond, the aniline sludge storages, the nitrobenzene pocket area, the TDI/TDA contaminated area and DNT installation [37]. Findings of the same studies conducted in and outside the Zachem CP area showed that the contaminated groundwater flowed from the Zachem area towards north-east and eastwards to the Vistula River and northwards to the Brda River and impacted the nearby residential area (ŁWS). Moreover, five probable partially overlapping plumes of groundwater contamination were identified in association with pollution sources: plume 1—incineration site in Żółwino, where, additionally, waste from heat and power plant was stored (this groundwater contamination source was unexpectedly identified outside the CP), plume 2—Zielona landfill complex, plume 3—nitrobenzene pocket or DNT installation, plume 4 and 5—central part of the former CP, including aniline sludge, Lisia landfill and EPI sediment pond [37].

### 2.2. Soil Sampling Approach and Selection of Contaminants

The soil sampling plan for the ŁWS was developed in compliance with the Regulation of the Minister of the Environment dated 1 September 2016 on the method of conducting the assessment of land surface contamination [36], based on the findings of the previous investigations conducted in the Zachem CP area by the AGH University of Science and Technology [37,38] and Arcadis [39]. Apart from that, numerical cartographic data in the form of a land and building registry map and a map of the ŁWS borders were used for this purpose.

According to the Regulation [38], the number of soil sampling sectors was established based on land use patterns (residential, industrial and agricultural) and the site area. Minimal number of soil samples for residential area is presented in Table 1.

**Figure 3:**
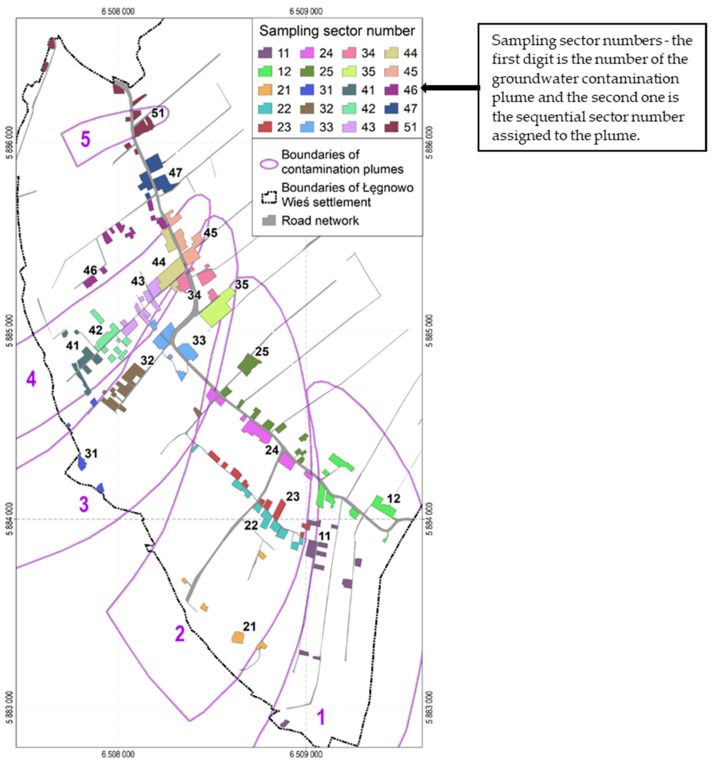
Location of soil sampling sectors in the residential development area of ŁWS against the background of groundwater contamination plumes (based on Czop et al. [37].

The ŁWS covers an area of about 736 ha. Residential buildings are situated on 164 plots, which constitute a residential development area of 31.1 ha. This area was divided into 20 soil sampling sectors, taking into account the location of groundwater contamination plumes and topographic relief. Most of the soil sampling sectors (seven) were within the extent of the contamination plume 4 and only one within the extent of the plume 5; five sampling sectors were within the extent of contamination plumes 2 and 3, and two were within the plume 1. Figure 3 presents the location of the soil sampling sectors in the residential development area of ŁWS against the background of groundwater contamination plumes. According to the Regulation [39], a total of 20 composite soil samples were taken, i.e., one in each soil sampling sector. Each composite soil sample was made up of at least 15 individual samples taken from the surface soil layer of 0–0.25 m. The composite soil samples were intended for measuring the content of substances in the surface layer as this is the soil layer people make contact with directly.

**Figure 4:**
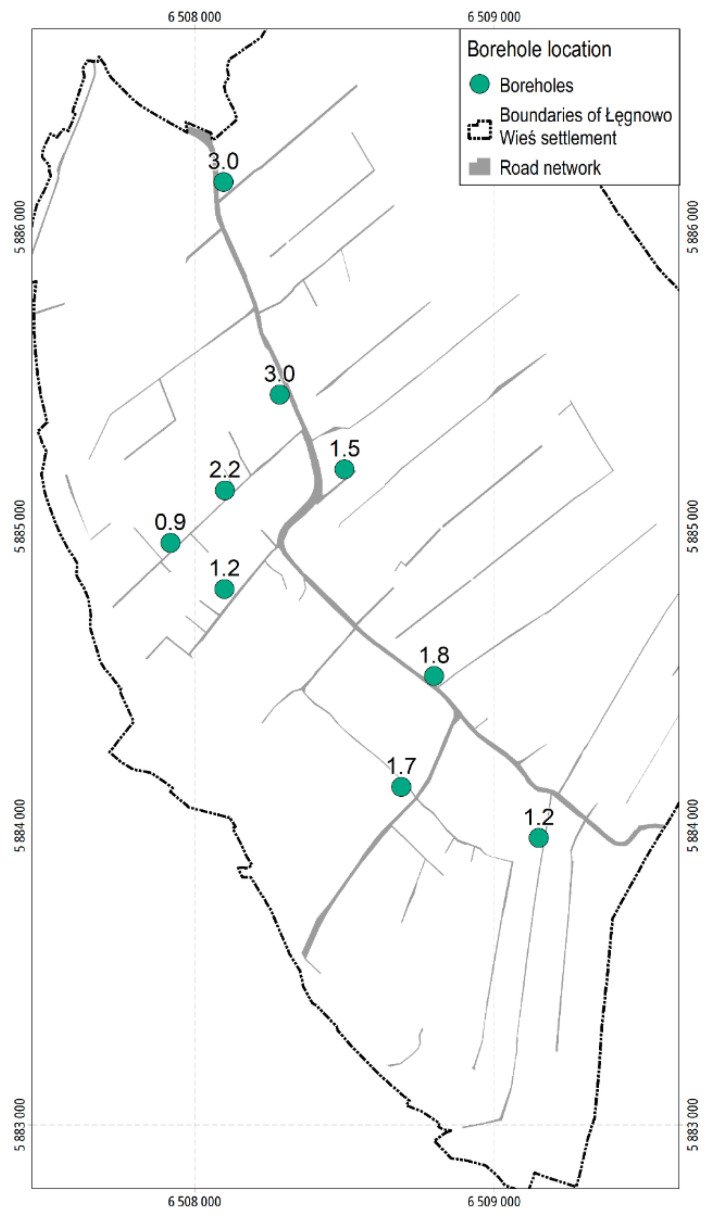
Location of boreholes in the residential development area of ŁWS (the number above the marked borehole means a soil sampling depth).

For identification of volatiles, it was necessary to collect samples from deeper soil layers. This was related to the possibility of penetration of substance vapours from deeper layers above the surface of the land, posing risk to human health. Therefore, 9 boreholes were made on the plots centrally located in the given group of residential plots within a given contamination plume. Samples were taken at the depths greater than 0.25 m, i.e., at the depths where contamination was expected to occur. Location of the boreholes along with the depth of the sampling are presented in Figure 4.

The substances for health risk analysis were selected, taking into consideration the location and boundaries of the groundwater contamination plumes in relation to the contamination sources. At the same time, it was indicated which substances should be determined in soil samples taken from the surface layer in particular sectors and which from the deeper layers (boreholes, volatile substances). This approach allowed selecting substances for risk assessment specifically for each sector of the residential area. It resulted in limiting the number of the analysed contaminants necessary to carry out the health risk assessment. The substance assignment is presented in Table 2.

**Figure 5:**
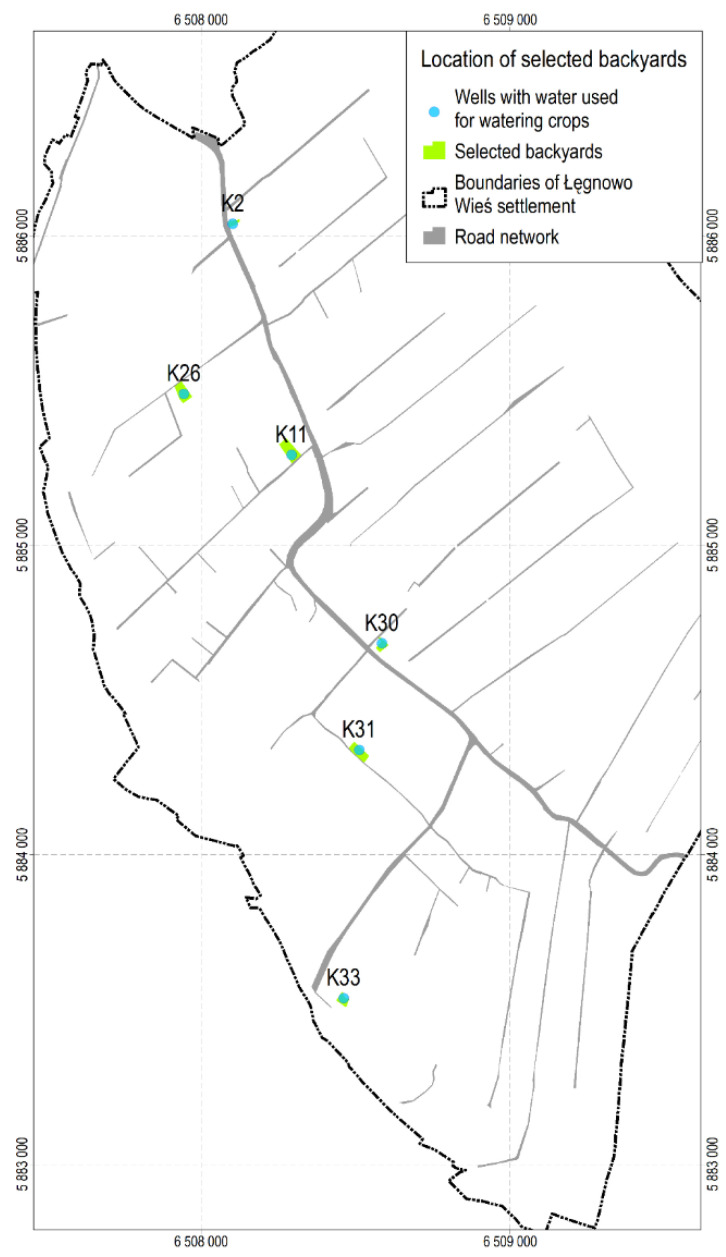
Location of backyards with marked wells.

Additionally, based on the reports of Czop et al. [36,37] and Arcadis [38], 6 backyards with wells from which water was used for watering home crops were selected, and one composite soil sample was taken from the surface layer (up to the depth of 0.25 m) in each of them. Location of the backyards with the marked wells (K02, K11, K26, K30, K31 and K33) is shown in Figure 5. The substances determined in the surface soil of the analysed backyards, assigned to a particular sector and plume number, are presented in Table 3.

### 2.3. Analytical Methods 

A total of 80 inorganic and organic substances were determined. The laboratory analyses were conducted in an accredited laboratory. The analytical methods applied for the determination of the substances are summarised in Table 4.

### 2.4. Health Risk Assessment (HRA)

In most European counties, the risk-based approaches are often applied for dealing with contaminated sites [1]. In Poland, health risk assessment is applied at contaminated sites, although it is not legally binding [40].

To assess the health risk to the residents of ŁWS, the HRA2 software developed by the Institute for Ecology of Industrial Areas (IETU), was applied [41]. The HRA2 software algorithms are based on the USEPA site-specific risk assessment methodology [42,43,44,45,46,47,48,49,50]. This methodology has been adapted to the Polish conditions and applied in practice in Poland since 1996 [51]. The USEPA human risk assessment is also widely used outside the United States [9,10,12,52,53,54,55,56,57].

The USEPA risk-based approach was also proposed for the process of contaminated land remediation in Poland [51]. Recently, its updated version has been proposed for the assessment of significant health risk [40], which is required in remediation plans and remediation project plans for contaminated soil under the Polish Environmental Protection Law (EPL), [58]. However, the risk assessment method has not been legally established as the reference one.

The site-specific HRA is based on site investigation data, including geochemical data and site-specific exposure conditions strictly related to the land use pattern. In the case of ŁWS it was the residential pattern. Exposure assessment describes the variables and their interactions that result in the exposure to contaminants. In the carried out study three exposure pathways were analysed: (1) incidental soil and dust ingestion (e.g. through hand-to-mouth contact), (2) dermal contact with soil, and (3) inhalation of fugitive soil particles and volatiles. These exposure pathways are the main pathways taken into account in risk assessment conducted in the contaminated sites [9,12,24,57].

Exposure to the soil contaminants was quantified separately for each exposure pathway: oral Exp_o_, dermal Exp_d_ and inhalation Exp_inh_, as shown in Equations (1)–(3), respectively [47,48,49,50]. The exposure was assessed separately for children and adults.

(1) $$Exp_{o}\;\left(mg\;kg^{-1}d^{-1}\right)=\frac{C\times EF\times ED\times IR_{o}\times CF_{1}\times RBA}{BW\times AT}\;\;\;$$

(2) $$Exp_{d}\left(mg\;kg^{-1}d^{-1}\right)=\frac{C\times EF\times ED\times SA\times AF\times ABS_{d}\times CF_{1}}{BW\times AT}\;\;$$

(3) $$Exp_{inh\;}\left(mg\;m^{-3}\right)\frac{C\times EF\times ED\times ET\times \left(\frac{1}{PEF}+\frac{1}{VF}\right)}{AT}\;\;\;\;\;\;$$

The applied parameter values are presented in Table 5. 

Concerning carcinogens with a mutagenic mode of action, the age dependent adjustment factors (ADAFs) were applied for calculating the residential pathway-specific exposure. ADAFs are related to the susceptibility to early-life exposure to such carcinogens. They refer to the following human life periods: 0–2 years (ADAF_0–2_ = 10), 2–6 years (ADAF_2–6_ = 3), 6–16 years (ADAF_6–16_ = 3) and 16–30 years (ADAF_16–30_ = 1) [50,59].

The health risks were characterised separately for non-carcinogenic and carcinogenic effects.

The non-cancer risk was estimated as the pathway-specific hazard quotient (oral HQ_o_, dermal HQ_d_ and inhalation HQ_inh_) through dividing the pathway-specific exposure by the relevant reference dose (oral RfD_o_, dermal RfD_d_) or the inhalation reference concentration (RfC), using Equations (4)–(6) [42,48,50].

(4) $$HQ_{o}=\frac{Exp_{o}}{RfD_{o}}$$

(5) $$HQ_{d}=\frac{Exp_{d}}{RfD_{d}}$$

(6) $$HQ_{inh}=\frac{Exp_{inh}}{RfC}$$

Summing the pathway-specific HQs, the hazard index (HI) for a given non-carcinogen was obtained, using Equation (7).

(7) $$HI=HQ_{o}+HQ_{d}+HQ_{inh}$$

The total hazard index (total HI) was obtained by summing the HIs calculated for all analysed non-carcinogens, as shown in Equation (8).

(8) $$Total\;HI=\overset{n}{\underset{i=1}{\sum }}HI_{i}$$

The total HI >1 means that there might be potential adverse health effects (USEPA, 1989).

Concerning the cancer risk, the pathway-specific cancer risk (oral CR_o_, dermal CR_d_ and inhalation CR_inh_) was estimated by the multiplication of the pathway-specific exposure and the relevant cancer slope factor (oral CSF_o_, dermal CSF_d_) or the inhalation unit risk (IUR), using Equations (9)–(11), [42,48,50].

(9) $$CR_{o}=Exp_{o}\times CSF_{o}$$

(10) $$CR_{d}=Exp_{d}\times CSF_{d}$$

(11) $$CR_{inh}=Exp_{inh}\times IUR$$

The cancer risk (CR) for a given non-carcinogen was obtained by summing the pathway-specific CRs, as shown in Equation (12).

(12) $$CR=CR_{o}+CR_{d}+CR_{inh}$$

The total cancer risk was estimated as a sum of CRs obtained for all analysed carcinogens, as shown in Equation (13).

(13) $$Total\;CR=\overset{n}{\underset{i=1}{\sum }}CR_{i}$$

The CRs were compared with the acceptable cancer risk values. The value of 1 × 10^−5^ (one in a hundred thousand) was used as the acceptable cancer risk for the individual carcinogen, as it is required in Poland [39]. In contrast, the value of 1 × 10^−1^ was used as the maximum acceptable total cancer risk according to the USEPA approach [43,44], and this was used by other authors as well [6,54,61,62].

The HRA results obtained in this study are presented for both types of receptors, i.e., children and adults for non-cancer effects and aggregate residents for cancer effects according to USEPA approach [45,47,48]. The aggregate receptor means an individual who is exposed in his/her childhood (for 6 years) and adult life (for 24 years). Aggregate cancer risk is calculated as the sum of child cancer risk and adult cancer risk [45,47,48,63]. The health risks were assessed separately for each sampling sector and each selected backyard.

### 2.5. Uncertainty in the Risk Assessment Process

Uncertainties are inherent in the risk assessment process and cannot be avoided. Uncertainties may be associated with data and analyses at each step of the risk assessment, e.g., the selection of substances, toxicity assessment and/or exposure assessment. Risk assessment results should be presented with the accompanying evaluation of the key uncertainties specific to the analysed chemicals and exposure pathways in order to support proper risk management decisions [64,65].

Uncertainties may be expressed either quantitatively or qualitatively, however, it depends on the applied risk assessment methodology—deterministic or probabilistic. In deterministic risk assessment, i.e., when relying on point values as inputs and presenting results also as point values, uncertainties may be discussed only qualitatively or semi-quantitatively. As for probabilistic risk analysis, using multiple risk estimates provides the likelihood of various risk levels, allowing uncertainties to be expressed quantitatively [66].

The health risk assessment conducted in the study is deterministic. Therefore, uncertainties could be addressed only qualitatively. This risk assessment applies conservative assumptions to obtain risk estimates protective for most of the potential receptors [64]. It uses default exposure parameter values that are selected to produce an overall estimate of exposure, which is at the higher end of the range of plausible exposures. Such an exposure is referred to as the reasonable maximum exposure (RME) and is defined as “the highest exposure that is reasonably expected to occur at a site” [42].

### 2.6. Developing the Site-Specific Health Risk-Based Target Levels (HRBTLs) for Soil Contaminants

Site-specific health risk-based target levels are estimated for the individual contaminants using the risk estimates obtained in the site-specific HRA [50,51,67]. They are calculated separately for non-cancer and cancer effects, using Equation (14) [50].

(14) $$HRBTL=C\times \frac{TR}{Calculated\;risk}$$

C: chemical content in soil (mg/kg)
HRBTL: health risk-based target level
TR: target risk (non-cancer target risk–TRN–HQ/HI or cancer target risk–TRC).

The HRBTLs were estimated for the most sensitive human receptors under the residential exposure scenario, i.e., children for non-cancer effects and aggregate residents for cancer effects [45,50]. In the case of non-cancer effects, the child life stage from birth to 6 years old is considered to be the most sensitive life stage. Due to the higher intake rate of soil by children and their lower body weight, the estimated exposure in children is greater than in adults [45,48]. For carcinogenic effects, the aggregate residents are considered to be sensitive receptors because the exposures are averaged over the whole (70 years) lifetime [45,48,63].

In the study, the HRBTLs correspond to the TR_N_ (HQ) of 1 for non-carcinogens as it is required by USEPA [50] and to the TR_C_ of 1 × 10^−5^ for carcinogens, according to [39]. If the contaminant produces both non-cancer and cancer effects, the non-cancer and cancer HRBTLs are compared and the lower of them is chosen as the final HRBTL.

## 3. Results and Discussion

### 3.1. Soil Contamination in Sampling Sectors and Backyards

The health risk assessment focused on substances, the content of which in the surface soil was equal to or higher than their limit of quantification (LOQ). The content of the volatiles determined in the deeper layer was not taken into account because only in one sample (sector 51) was the content of benzo(a)anthracene and in two samples was the content of pyrene (sectors 25 and 51) higher than their LOQ but lower than in the surface layer. The content of 3,5-dichlorophenol was equal to its LOQ in only one sample, in other sectors being lower than its LOQ. Taking into account that the relevant toxicity value is unavailable, this substance was not considered in the HRA. Ultimately, the content of metals, arsenic, PAHs and phenol in the surface soil was the input data for the assessment of health risk to ŁWS residents (see Supplementary Material 1; Table S1a,b).

The descriptive statistics of these substances, along with their worldwide background mean content, are shown in Table 7. The coefficient of variation (CV) estimated for As and heavy metals shows lower content variability of these elements. The CVs for all of them are below 0.6. The CV estimated for PAHs shows very high content variability of all PAHs, especially in the sampling sectors. The highest CV is observed for fluorene (CV = 3.01) and for naphthalene, anthracene, acenaphthene, phenanthrene and fluoranthene (CV above 2). Very high variability in the content of all PAHs may indicate a different origin of PAH contamination than from the Zachem CP.

When compared with the worldwide background mean content, it can be observed that in the analysed sectors and backyards the mean content of arsenic and all heavy metals, except for Zn, was lower than the relevant background content. The copper content in the backyard soil was only slightly higher than the corresponding background level. The mean contents of arsenic and most heavy metals are below their background values, which indicates that these elements are of natural origin.

To assess soil contamination with PAHs, the criteria for the sum of PAHs proposed by Maliszewska-Kordybach [69] were applied. According to them, the sum of 16 PAHs’ content in the soil below 0.2 mg/kg indicates that the soil is considered to be uncontaminated. The sum of 16 PAHs’ content is shown in Table 8.

The sum of 16 PAHs’ content was above the threshold value of 0.2 mg/kg in most of the sectors and all backyards. Only in two sectors (41 and 42), was the sum of 16 PAHs equal to 0.2 mg/kg. The highest sum of 16 PAHs’ content was found in sector 32, exceeding by 378 times the threshold value. Among the backyards, the highest sum of 16 PAHs’ content was found in backyard K30, exceeding the value of 0.2 mg/kg by about 60 times. The mean content of 16 PAHs estimated for all sectors (9.5 mg/kg) was 47.5 times higher than the threshold value and several times higher than those reported for different urban areas, e.g., Beijing, China (1.23 mg/kg), [70], Bergen, Norway (6.78 mg/kg), [71] and Lisbon, Portugal (1.54 mg/kg), [10].

### 3.2. Health Risk Assessment in Sampling Sectors

The total non-cancer risks (total HIs) calculated for children and adults and the total CRs calculated for aggregate residents in all sampling sectors are shown in Table 9. More detailed results, including the HQs, HI and CRs estimated for the individual contaminant in each sampling sectors are shown in Tables S1–S60 (see Supplementary Material 2).

The total HIs for children vary from HI = 0.001 in sectors 41 and 42 to HI = 0.35 in sector 24. They are significantly lower than the TR_N_ of 1. The total HIs for adults range from HI = 0.00022 in sector 41 to HI = 0.045 in sector 32 and are obviously lower than for children in all sampling sectors. Due to hand- and object-to-mouth behaviour, young children unintentionally ingest more soil than adults, which results in higher HIs [45,48,72,73,74].

**Figure 6:**
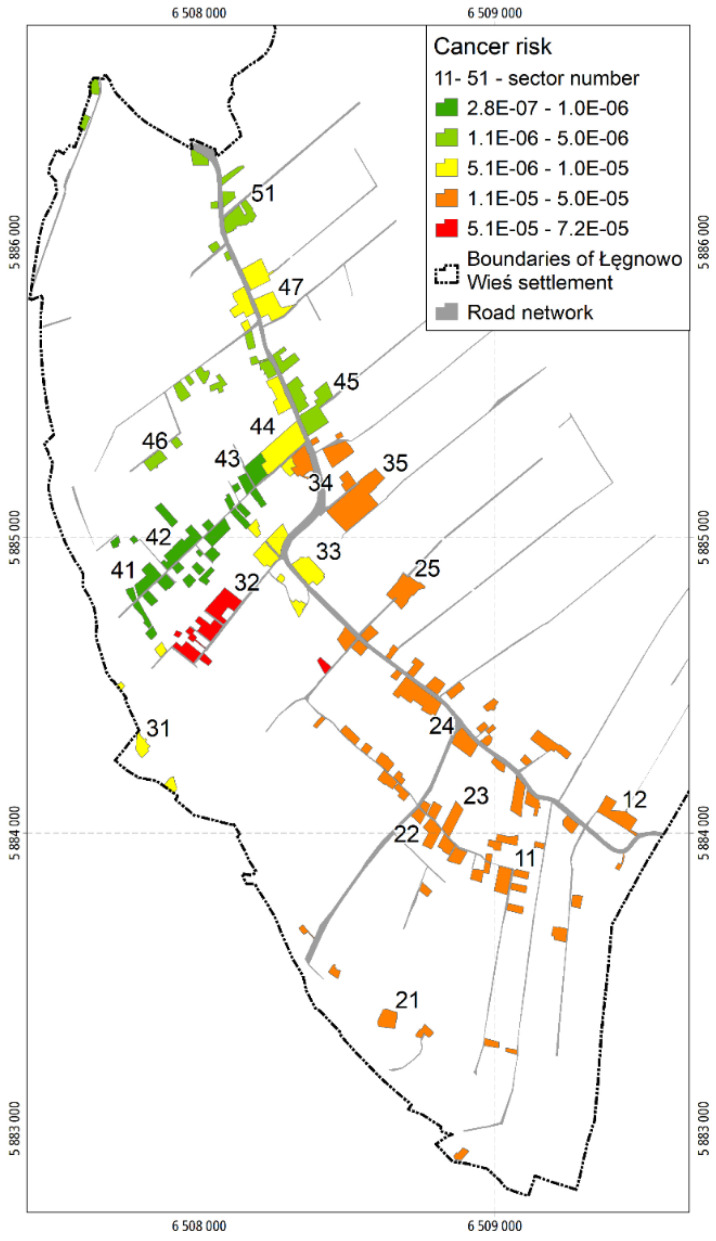
Map of total cancer risks in sectors.

The total CRs vary from CR = 2.8 × 10^−7^ in sectors 41 and 42 to CR = 7.2 × 10^−5^ in sector 32. In no sector does the total CR exceed the TR_C_ of 1 × 10^−4^. The total CRs in the sampling sectors are illustrated in Figure 6.

Considering the individual carcinogens, only CRs calculated for benzo(a)pyrene and chromium (VI) are insignificantly higher than 1 × 10^−5^ and relate to sectors 32, 34, 35 (Tables S27, S33 and S36) and sectors 23 and 24 (Tables S15 and S18), respectively (see Supplementary Material 2).

As for chromium (VI), the CR risk is subject to uncertainty because it was calculated assuming that the content of chromium (VI) in soil is 1/7 of the total chromium content [75], which is not necessarily reflected in local conditions.

### 3.3. Health Risk Assessment in Backyards

The total non-cancer risks (total HIs) calculated for children and adults and the total CRs calculated for aggregate residents in all analysed backyards are shown in Table 10. More detailed results, including the HQs, HI and CRs estimated for the individual contaminant in each backyard are shown in Tables S61–S78 (see Supplementary Material 2).

The total HIs for children vary from HI = 0.0051 (backyard K26) to HI = 0.40 (backyard 30). They are significantly lower than the TR_N_ of 1. The total HIs for adults vary from HI = 0.00071 (backyard K26) to HI = 0.046 (backyard K30) and are lower than for children in all analysed backyards.

**Figure 7:**
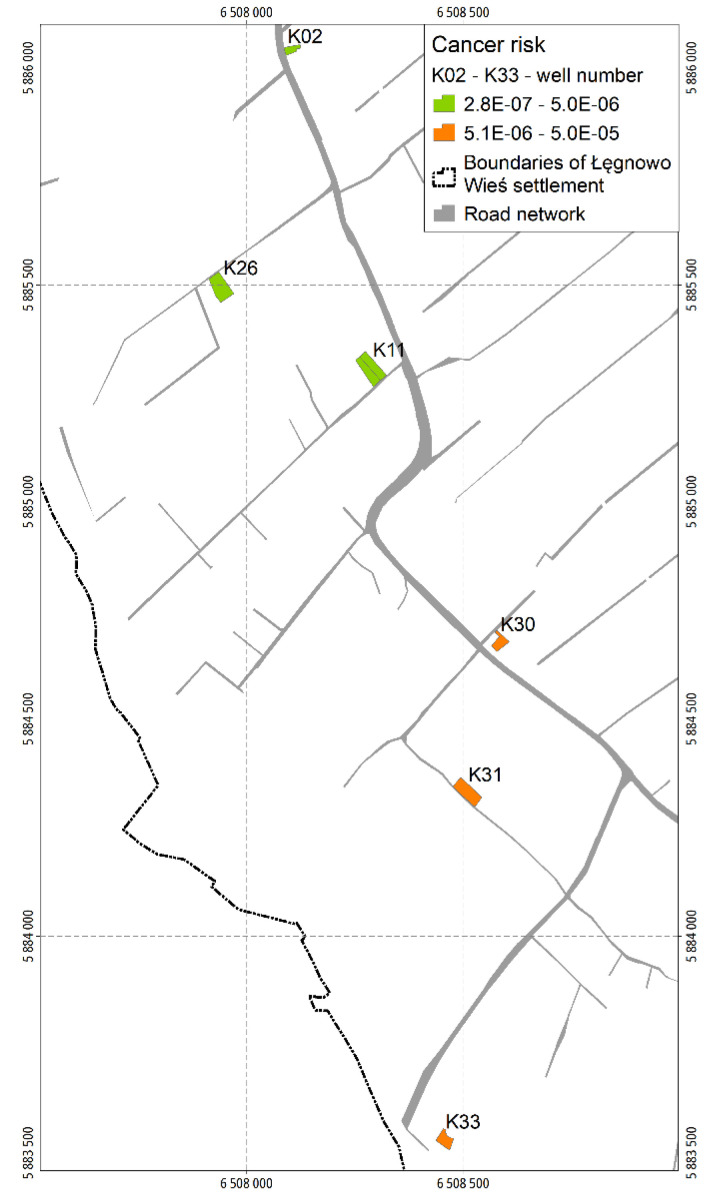
Map of total cancer risks in backyards.

The total CRs vary from CR = 1.2 × 10^−6^ (backyard K26) to CR = 3.4 × 10^−5^ (backyard K30). In no backyard does the total CR exceed the TR_C_ of 1 × 10^−4^. The total CRs in the backyards are presented also in Figure 7.

As in the case of the sampling sectors, only CRs calculated for benzo(a)pyrene and chromium (VI) are insignificantly higher than 1 × 10^−5^ and relate to the backyard K30. Their values amount to 1.2 × 10^−5^ for both benzo(a)pyrene and chromium (VI) (see Table S72 in Supplementary Material 2).

Soil ingestion is the main exposure pathway and oral HIs and oral CRs mostly contribute to the total HIs and total CRs, respectively (see Tables S1–S78). It should be noticed that direct ingestion is generally considered to be the most important pathway in human exposure to soil contaminants, constituting the basis for soil remediation [72,76]. 

### 3.4. Uncertainties of the Risk Assessment 

The presented risk estimates may be accompanied by uncertainties. They may be related to the substance selection, exposure assumptions, toxicity assessment and risk characterisation, as described below.

(1) Selection of substances

The ŁWS contaminants were selected based on the contamination sources and the most probable location of the groundwater contamination plumes delineated in 2017 [37]. However, the extent of the identified plums may change as further investigation is carried out. This would require the installation of additional piezometers, as well as the collection and application of new data on contaminant content in the groundwater. The application of updated data could improve the quality of information about the location and boundaries of the plumes and their contamination. As a consequence, it could result in the verification of the list of selected contaminants.

(2) Exposure assumptions

The site-specific exposure parameter values for the residents of the ŁWS were unavailable; therefore, the defaults suggested by Wcisło [40,51] were used. These values were generally adapted from USEPA documents [47,48,49,50]; however, they can be considered as realistic estimates to be applied to calculate RME in Polish conditions when the site-specific data are unavailable or incomplete. Moreover, it should be noted that although assumptions made in the residential exposure scenario were based on the best professional judgement, they may not be accurate for specific individuals whose characteristics may vary from the conservative generic conditions.

Another uncertainty may be associated with the assumption that the residents’ exposure to the site contaminants remains constant over time under the present conditions, although the actual conditions in the site are more likely to reflect an intermittent or irregular exposure situation.

(3) Toxicity assessment

The substances that are simultaneously present in the mixture, which is the case in ŁWS, may interact and cause different toxic effects than the individual substances separately. Potential interactions between components have been described as additive, synergistic, antagonistic and potentiating [64,77]. However, there are still many gaps in knowledge and data regarding the quantification of these interactions and the assessment of the toxicity of the chemical mixtures, despite the fact that many new methods and approaches have been recently developed [77,78,79,80,81]. Due to the lack of adequate knowledge, the potential interactions between the selected contaminants could not be considered in the HRA for the ŁWS.

Another uncertainty may be related to the use of parent organic compounds in the HRA, although their toxic derivatives may also be present in the ŁWS soil. However, it was not possible to predict which derivatives were potentially present in the soil.

Another area of uncertainty is the use of the USEPA-derived toxicity measures, i.e., cancer slope factors, reference doses and reference concentrations 50]. They are used as single point estimates that have the uncertainty associated with their derivation. On the other hand, it should be emphasised that they are derived to be conservative and provide upper bound risk estimates [82].

(4) Risk characterisation

The uncertainties in the risk characterisation result from the uncertainties identified in the previous steps of the risk assessment. Because of the lack of scientific knowledge about the interactions among the substances occurring in the ŁWS soil, risk additivity was applied to calculate HIs and CRs.

The uncertainties in the HRA for the ŁWS residents could only be described in qualitative terms, and it was impossible to make scientifically rigorous statements about the impact of these uncertainties on the HRA results. It should be emphasised, however, that the assumptions made in the risk assessment are conservative and help prevent the underestimation of non-cancer health effects or cancer risk in the ŁWS. This protective approach is reflected in the use of conservative toxicity measures and default exposure parameter values that represent RME.

Summing up, the risk assessment findings show that both non-cancer and cancer risks are below the acceptable risk levels, i.e., total hazard index equal to 1 and total cancer risk of 1 × 10^−4^, respectively. They can be considered to be protective for the majority of residents in Łęgnowo-Wieś settlement.

### 3.5. Site-Specific Health Risk-Based Target Levels (HRBTLs) for Soil Contaminants

The HRBTLs developed for soil contaminants are presented in Table 11. For each HRBTL, the effect (cancer, non-cancer or mutagenic) was indicated, which was the basis for deriving these values.

The HBRLs were compared to the soil contaminant content determined in each sector and backyard (see Supplementary Material—Table S1a,b). The findings show that the content of all the analysed contaminants are lower than their HRBTLs, except for benzo(a)pyrene. B(a)P content, as the only one, was insignificantly higher than the HBRL of 1.1 mg/kg in three sectors (32, 34 and 35) and one backyard (K30), which is consistent with the HRA results.

It should be noticed, however, that the total cancer risks in all sectors and backyards do not exceed the acceptable total cancer risk of 1 × 10^−4^. Taking into consideration that this risk assessment applies conservative assumptions, it can be concluded that the soil in the residential area is safe for the ŁWS residents.

Nevertheless, it must be remembered that the health risk was assessed taking into account only direct soil exposure pathways. It did not include other possible exposure pathways, for example, those resulting from the consumption of fruit from home gardens and local arable crops [14,15,28,85]. Fruit contamination can occur even when soil is uncontaminated if the fruit tree roots reach the contaminated groundwater. The arable crops, on the other hand, can be contaminated if they are cultivated in the area with a high level of groundwater, as is the case in ŁWS. Therefore, further research should take into account the exposure of ŁWS residents to potential contaminants through the consumption of locally grown food.

## 4. Conclusions

The health risk posed to the residents of the Łęgnowo-Wieś settlement situated in the vicinity of the former Zachem Chemical Plant was assessed. Due to the unique site history and site contamination profile, a specific strategy for soil sampling and contaminant selection was proposed. The strategy is innovative, entirely developed by the authors. Taking into account the location and boundaries of the groundwater contamination plumes in relation to contamination sources, the strategy allowed selecting substances for risk assessment specifically for each sector of the residential area. It resulted in limiting the number of the analysed contaminants necessary to carry out the assessment. In spite of many contamination sources being identified at the site and the complexity of the contamination, the risk assessment findings show that both non-cancer and cancer risks are below their acceptable risk levels, i.e., total hazard index equal to 1 and total cancer risk of 1 × 10^−4^, respectively. Taking into consideration that this risk assessment applies conservative assumptions, it can be concluded that the soil in the residential area is safe for the ŁWS residents and no remedial actions are required. However, it must be pointed out that this conclusion refers only to direct soil exposure pathways, i.e., incidental ingestion, dermal contact and inhalation, which means that other potential exposure pathways cannot be neglected, e.g., consumption of fruit from home gardens and local arable crops. Therefore, further research focused on the assessment of health risk resulting from the consumption of locally grown food would be highly recommended.

## Figures and Tables

**Table 1:** Minimal number of soil samples in sampling sectors for residential area [39].

Site Area (ha)	Sector Area (ha)	Number of Sectors (Composite Samples)	Number of Individual Soil Samples
≤0.05	≤0.05	1	15
0.05–1	(0.05/3)–0.1	3–10	45–150
1–10	0.1–0.5	10–20	150–300
>10	≤5	≥20	≥300

**Table 2:** Assignment of substances to contamination sources and plumes, broken down into substances taken from the surface layer and deeper layers.

Sector No. ^a^	Area (ha)	Plume No.	Contamination Sources	Substances—Soil Surface Layer (0–0.25 m)	Number of Boreholes	Volatiles
					Soil Layers Below 0.25 m Depth	
11	1.6669	1	Incineration site in Żółwino with waste from heat and power plant	PAHs (16 USEPA-regulated PAHs) ^b^, phenol, metals ^c^ and arsenic (As)	1	PAHs (anthracene, acenaphthene, fluorene, pyrene, naphthalene, benzo(a)anthracene)
12	2.0672					
21	0.7400	2	Zielona landfill	PAHs (16 USEPA-regulated PAHs) ^b^, metals ^c^ and As, phenol, chlorophenols, toluene, aniline, chloroaniline, toluidine, 2-phenylphenol, ethylene glycol, diphenyl sulphone, trichloroethene (TCE), tetrachloroethene (PCE), octylphenols	2	PAHs (anthracene, acenaphthene, fluorene, pyrene, naphthalene, benzo(a)anthracene), toluene
22	1.5726					
23	1.3557					
24	1.9670					
25	1.8549					
31	0.5320	3	Nitrobenzene pocket or dinitrotoluene installation	PAHs (16 USEPA–regulated PAHs) ^b^, phenol, nitrobenzene, o-nitrotoluene, dinitrotoluene mixture (2,4/2,6-)	2	PAHs (anthracene, acenaphthene, fluorene, pyrene, naphthalene, benzo(a)anthracene), nitrobenzene, o-nitrotoluene
32	1.7503					
33	1.7314					
34	1.3277					
35	1.7811					
41	1.2494	4	Central part of Zachem CP, incl. aniline sludge, Lisia landfill, epichlorohydrin (EPI) sediment pond	Aniline, chloroaniline, epichlorohydrin, toluenediamine (TDA), toluene diisocyanate (TDI), phenol, PAHs (16 USEPA–regulated PAHs) ^b^ nitrobenzene, toluidine, o-nitrotoluene, dinitrotoluene mixture (2,4/2,6-), toluene	3	Epichlorohydrin, toluene diisocyanate (TDI), PAHs (anthracene, acenaphthene, fluorene, pyrene, naphthalene, benzo(a)anthracene), nitrobenzene, o-nitrotoluene, toluene
42	1.4752					
43	1.6802					
44	1.8907					
45	1.6507					
46	1.3698					
47	1.8290					
51	1.6121	5	Central part of the Zachem CP, incl. aniline sludge, Lisia landfill, epichlorohydrin (EPI) sediment pond	Aniline, chloroaniline, epichlorohydrin, toluenediamine (TDA), toluene diisocyanate (TDI), phenol, PAHs (16 USEPA–regulated PAHs ^b^), nitrobenzene, toluidine, o-nitrotoluene, dinitrotoluene mixture (2,4/2,6-)	1	Epichlorohydrin, toluene diisocyanate (TDI), PAHs (anthracene, acenaphthene, fluorene, pyrene, naphthalene, benzo(a)anthracene), nitrobenzene, o-nitrotoluene

^a^: The first digit is the number of the groundwater contamination plume and the second one is the sequential sector number assigned to the plume. ^b^: 16 PAHs: naphthalene, acenaphthylene, acenaphthene, fluorene, phenanthrene, anthracene, fluoranthene, pyrene, benzo(a)anthracene, chrysene, benzo(b)fluoranthene, benzo(k)fluoranthene, benzo(a)pyrene, dibenzo(a,h)anthracene, benzo(g,h,i)perylene, indeno(1,2,3-cd)pyrene. ^c^ Metals: barium (Ba), chromium (Cr), tin (Sn), zinc (Zn), cadmium (Cd), cobalt (Co), copper (Cu), molybdenum (Mo), nickel (Ni), lead (Pb), mercury (Hg).

**Table 3:** Substances determined in the surface soil of the backyards, assigned to the sector and plume number (based on Czop et al. [36,37] and Arcadis [38].

Backyard (Well No.)	Sector No.	Area (m^2^)	Plume No.	Substances Determined in the Soil Surface Layer (0–0.25 m)
K02	51	522.2	5	Aniline, chloroaniline, epichlorohydrin, toluenediamine (TDA), toluene diisocyanate (TDI), phenol, PAHs (16 USEPA–regulated PAHs) ^a^, nitrobenzene, toluidine, o-nitrotoluene, dinitrotoluene mixture (2,4/2,6-)
K11	44	2568.0	4	Aniline, chloroaniline, epichlorohydrin, toluenediamine (TDA), toluene diisocyanate (TDI), phenol, PAHs (16 USEPA–regulated PAHs) ^a^, nitrobenzene, toluidine, o-nitrotoluene, dinitrotoluene mixture (2,4/2,6-), toluene
K26	46	2080.0		
K30	25	856.6	2	PAHs (16 USEPA–regulated PAHs) ^a^, metals ^b^ and As, phenol, chlorophenols, toluene, aniline, chloroaniline, toluidine, 2-phenylphenol, ethylene glycol, diphenyl sulphone, trichloroethene (TCE), tetrachloroethene (PCE), octylphenols
K31	23	1896.0		
K33	21	984.7		

^a^: 16 PAHs (polycyclic aromatic hydrocarbons): naphthalene, acenaphthylene, acenaphthene, fluorene, phenanthrene, anthracene, fluoranthene, pyrene, benzo(a)anthracene, chrysene, benzo(b)fluoranthene, benzo(k)fluoranthene, benzo(a)pyrene, dibenzo(a,h)anthracene, benzo(g,h,i)perylene, indeno(1,2,3-cd)pyrene. ^b^: Metals: barium (Ba), chromium (Cr), tin (Sn), zinc (Zn), cadmium (Cd), cobalt (Co), copper (Cu), molybdenum (Mo), nickel (Ni), lead (Pb), mercury (Hg).

**Table 4:** Analytical methods applied for the determination of substances.

Substance	Method
2,3-toluenediamine, 2,4-toluenediamine, 2,6-toluenediamine	ISO 11916-1: 2014-11, LC-UV
2,4-dinitrotoluene, 2,6-dinitrotoluene, o-nitrotoluene	ISO 11916-1: 2014-11, LC-UV
2-chloroanilin, 3-chloroanilin, 4-chloroanilin, aniline	DIN 38407-16: 1999-06, GC-MS
2-phenylphenol	Non-standard method, GC-MS-/MS
2-toluidine, 3-toluidine, 4-toluidine, nitrobenzene	ISO 11916-1: 2014-11, LC-UV
Arsenic and heavy metals	PN-EN 16171:2017-02, ICP-MS
Benzene, toluene, ethylbenzene, xylenes (BTEX)	PN-EN ISO 22155:2016-07, HS-GC-MS
Chlorophenols	Non-standard method, GC-MS
Diphenyl sulphone	Non-standard method, GC-MS-MS
Epichlorohydrin	Non-standard method, HS-GC-MS
Ethylene glycol	Non-standard method, GC-FID
Octylphenols	Non-standard method, GC-MS-/MS
Phenol	Non-standard method, GC-MS
Polycyclic aromatic hydrocarbons	PN-ISO 18287:2008; GC-MS
Tetrachloroethene Trichloroethene	NEN ISO 22155, HS-GC-MS
Toluene diisocyanate	Non-standard method, LC-MS-MS

ICP-MS: inductively coupled plasma mass spectrometry; GC-MS: liquid chromatography–mass spectrometry; LC-UV: liquid chromatography–ultraviolet; HS-GC-MS: headspace gas chromatography–mass spectrometry; LC-MS-MS: liquid chromatography–tandem mass spectrometry; GC-FID: gas chromatography–flame ionisation detection; GC-MS-MS: gas chromatography–tandem mass spectrometry.

**Table 5:** Parameter values applied for health risk assessment in ŁWS [43,44,48,49].

Parameter	Definition (Unit)	Value
ABS_d_	dermal absorption fraction (unitless)	chemical-specific (see Table 6)
ABS_GI_	gastrointestinal absorption factor (unitless)	chemical-specific (see Table 6)
AF (adult)	soil-to-skin adherence factor (mg/cm^2^/day)	0.07
AF (child)	soil-to-skin adherence factor (mg/cm^2^/day)	0.2
AT_N_ (adult)	averaging time for non-carcinogens (days); AT_N_ = ED × 365 days	8760
AT_N_ (child)	averaging time for non-carcinogens (days); AT_N_ = ED × 365 days	2190
AT_C_	averaging time (carcinogens) (days); AT_C_ = 70 years × 365 days	25,550
BW (adult)	body weight (kg)	70
BW (child)	body weight (kg)	15
C	contaminant content in the surface soil	(see Supplementary Material 1; Table S1a,b)
CF_1_	conversion factor (kg/mg)	1 × 10^−6^
CSF_d_	dermally adjusted cancer slope factor (mg/kg/day)^−1^	chemical-specific (CSF_d_ = CSF_o_/ABS_GI_)
CSF_o_	oral cancer slope factor (mg/kg/day)^−1^	chemical-specific (see Table 6)
ED (adult)	exposure duration (years)	24
ED (child)	exposure duration (years)	6
EF	exposure frequency (days/year)	350
ET	exposure time in a day (h/h)	1 (24h/24h, i.e. whole day)
IR_o_ (adult)	ingestion rate (mg/day)	100
IR_o_ (child)	ingestion rate (mg/day)	200
IUR	inhalation unit risk (mg/m^3^)^−1^	chemical-specific (see Table 6)
PEF	particulate emission factor (m^3^/kg)	sector/backyard area-specific; calculated according to USEPA (2002)
RBA	relative bioavailability factor	0.6 for As; 1 for other substances
RfC	inhalation reference concentration (mg/m^3^)	chemical-specific (see Table 6)
RfD_d_	dermally adjusted reference dose (mg/kg/day)	chemical-specific (RfD_d_ = RfD_o_ × ABS_GI_)
RfD_o_	oral reference dose (mg/m^3^)	chemical-specific (see Table 6)
SA (adult)	skin surface area exposed (cm^2^)	5700
SA (child)	skin surface area exposed (cm^2^)	2800
TR_C_	cancer target risk	1 × 10^−5^ [39]
TR_N_	non-cancer target risk	1
VF	volatile factor (m^3^/kg)	chemical-specific and sector/backyard area-specific; calculated according to USEPA [48,50]

**Table 6:** Chemical-specific parameter values [49,50,60].

Substance		CAS Number		RfD_o_ (mg/kg/day)	RfC (mg/m^3^)	CSF_o_ (mg/kg/day)^−1^	IUR (µg/m^3^)^−1^	ABS_GI_ (Unitless)	ABS_d_ (Unitless)
Metals and metalloids									
Arsenic		007440-38-2		3.0 × 10^−4^	1.5 × 10^−5^	1.50	4.3 × 10^−3^	1	0.03
Barium		007440-39-3		2.0 × 10^−1^	5.0 × 10^−4^	NA	NA	0.07	0.01
Cadmium		007440-43-9		1.0 × 10^−3^	1.0 × 10^−5^	NA	1.8 × 10^−3^	0.025	0.001
Chromium (III)		016065-83-1		1.5	NA	NA	NA	0.013	0.01
Chromium (VI)	M	018540-29-9		3.0 × 10^−3^	1.0 × 10^−4^	5.00 × 10^−1^	8.4 × 10^−2^	0.025	0.01
Cobalt		007440-48-4		3.0 × 10^−4^	6.0 × 10^−6^	NA	9.0 × 10^−3^	1	0.01
Copper		007440-50-8		4.0 × 10^−2^	NA	NA	NA	1	0.01
Mercury		007487-94-7		3.0 × 10^−4^	3.0 × 10^−4^	NA	NA	0.07	0.01
Molybdenum		007439-98-7		5.0 × 10^−3^	NA	NA	NA	1	0.01
Nickel		007440-02-0		2.0 × 10^−2^	9.0 × 10^−5^	NA	2.6 × 10^−4^	0.04	0.01
Tin		007440-31-5		6.0 × 10^−1^	NA	NA	NA	1	0.01
Zinc		007440-66-6		3.0 × 10^−1^	NA	NA	NA	1	0.01
Polycyclic aromatic hydrocarbons (PAHs)									
Acenaphthene		000083-32-9		6.0 × 10^−2^	NA	NA	NA	1	0.13
Acenaphthylene *		000208-96-8		NA	NA	NA	NA	1	0.13
Anthracene		000120-12-7		3.0 × 10^−1^	NA	NA	NA	1	0.13
Benzo(a)anthracene	M	000056-55-3		NA	NA	1.00 × 10^−1^	6.0 × 10^−5^	1	0.13
Benzo(a)pyrene	M	000050-32-8		3.0 × 10^−4^	2.0 × 10^−6^	1.00	6.0 × 10^−4^	1	0.13
Benzo(b)fluoranthene	M	000205-99-2		NA	NA	1.00 × 10^−1^	6.0 × 10^−5^	1	0.13
Benzo(g,h,i)perylene *		000191-24-2		NA	NA	NA	NA	1	0.13
Benzo(k)fluoranthene	M	000207-08-9		NA	NA	1.00 × 10^−2^	6.0 × 10^−6^	1	0.13
Chrysene	M	000218-01-9		NA	NA	1.00 × 10^−3^	6.0 × 10^−7^	1	0.13
Dibenzo(a,h)anthracene	M	000053-70-3		NA	NA	1.00	6.0 × 10^−4^	1	0.13
Fluoranthene		000206-44-0		4.0 × 10^−2^	NA	NA	NA	1	0.13
Fluorene		000086-73-7		4.0 × 10^−2^	NA	NA	NA	1	0.13
Phenanthrene *		000085-01-8		NA	NA	NA	NA	1	0.13
Indeno(1,2,3-cd)pyrene	M	000193-39-5		NA	NA	1.00 × 10^−1^	6.0 × 10^−5^	1	0.13
Naphthalene		000091-20-3		2.0 × 10^−2^	3.0 × 10^−3^	NA	3.4 × 10^−5^	1	0.13
Pyrene		000129-00-0		3.0 × 10^−2^	NA	NA	NA	1	0.13
Other substances									
3,5-dichlorophenol *		000591-35-5		NA	NA	NA	NA	NA	NA
Phenol		000108-95-2		3.0 × 10^−1^	2.0 × 10^−1^	NA	NA	1	0.1

NA: not available; M: mutagenic substance; RfD_o_: oral reference dose; CSF_o_: oral cancer slope factor; RfC: reference concentration; IUR: inhalation unit risk; ABS_GI_: gastrointestinal absorption factor; ABS_d_: dermal absorption fraction; *: substance skipped in the health risk assessment due to the lack of data on its toxicity measures.

**Table 7:** Descriptive statistics of substances analysed in sectors and backyards.

Substance	N	Minimum (mg/kg)	Maximum (mg/kg)	Mean (mg/kg)	GM (mg/kg)	SD	CV	Worldwide Background Mean Content (mg/kg) [68]
Sampling sectors								
Arsenic and heavy metals								
As	7	1.64	4.37	2.72	2.59	0.94	0.35	4.7
Ba	7	40.20	127.00	81.56	77.30	27.49	0.34	362
Co	7	1.28	3.95	2.48	2.34	0.88	0.35	6.9
Cr	7	4.81	14.60	8.62	8.11	3.33	0.39	42
Cu	7	7.52	14.20	10.44	10.24	2.21	0.21	14
Hg	7	*0.05*	*0.05*	*0.05*	*0.05*	0.00	NA	0.1
Ni	7	3.22	15.00	7.34	6.59	3.88	0.53	18
Pb	7	12.00	25.70	17.31	16.74	4.90	0.28	25
Sn	7	*0.50*	1.49	0.84	0.75	0.45	0.54	-
Zn	7	54.70	156.00	102.74	97.26	35.25	0.34	62
PAHs and phenol								
ACTE	20	*0.0125*	0.7390	0.0554	0.0202	0.1614	2.92	-
ACTY	20	*0.0125*	0.0760	0.0260	0.0218	0.0166	0.64	-
ANT	20	*0.0125*	2.9500	0.3076	0.0846	0.7410	2.41	-
B(a)A	20	*0.0125*	6.3800	0.8670	0.3214	1.5868	1.83	-
B(a)P	20	*0.0125*	5.0980	0.8231	0.3439	1.2643	1.54	-
B(b)F	20	*0.0125*	7.5100	1.1633	0.4686	1.7990	1.55	-
B(g,h,i)P	20	*0.0125*	2.8800	0.5270	0.2480	0.7133	1.35	-
B(k)F	20	*0.0125*	2.5900	0.4171	0.1896	0.6256	1.50	-
CHR	20	*0.0125*	6.7200	0.9126	0.3412	1.5991	1.75	-
D(a,h)A	20	*0.0125*	0.8780	0.1112	0.0358	0.2131	1.92	-
FEN	20	*0.0125*	11.4000	0.8796	0.2752	2.4875	2.83	-
FLU	20	*0.0125*	0.9720	0.0706	0.0238	0.2127	3.01	-
FLUA	20	*0.0125*	14.0000	1.4941	0.5718	3.0549	2.04	-
I(1,2,3-cd)P	20	*0.0125*	3.6600	0.5970	0.2762	0.8567	1.43	-
NAF	20	*0.0125*	0.4910	0.0522	0.0177	0.1249	2.40	-
PIR	20	*0.0125*	10.3000	1.1927	0.4875	2.2556	1.89	-
Phenol	20	*0.0050*	0.0100	0.0053	0.0052	0.0011	0.21	-
Backyards								
Arsenic and heavy metals								
As	3	2.53	3.94	3.26	3.21	0.71	0.22	4.7
Ba	3	46.20	125.00	82.13	75.73	39.85	0.49	362
Co	3	1.85	3.30	2.48	2.41	0.74	0.30	6.9
Cr	3	5.26	11.30	8.00	7.62	3.06	0.38	42
Cu	3	9.40	18.10	14.57	14.02	4.57	0.31	14
Hg	3	0.05	0.12	0.07	0.07	0.04	0.57	0.1
Ni	3	4.60	10.40	7.29	6.90	2.92	0.40	18
Pb	3	14.50	31.00	21.83	20.79	8.40	0.38	25
Sn	3	0.50	1.44	1.11	1.00	0.53	0.48	-
Zn	3	58.70	162.00	98.00	88.67	55.90	0.57	62
PAHs and phenol								
ACTE	6	*0.0125*	0.0430	0.0176	0.0154	0.0125	0.71	-
ACTY	6	*0.0125*	0.0460	0.0181	0.0155	0.0137	0.76	-
ANT	6	0.0125	0.1970	0.0599	0.0405	0.0683	1.14	-
B(a)A	6	0.0680	0.7230	0.2777	0.2182	0.2281	0.82	-
B(a)P	6	0.0820	1.3400	0.4007	0.2700	0.4655	1.16	-
B(b)F	6	0.1360	1.8700	0.5632	0.3870	0.6471	1.15	-
B(g,h,i)P	6	0.0610	0.9660	0.3003	0.2067	0.3316	1.10	-
B(k)F	6	0.0380	0.6070	0.1940	0.1355	0.2064	1.06	-
CHR	6	0.0730	1.1500	0.3330	0.2191	0.4044	1.21	-
D(a,h)A	6	*0.0125*	*0.0125*	*0.0125*	*0.0125*	0.0000	NA	-
FEN	6	0.0500	0.5170	0.1970	0.1526	0.1658	0.84	-
FLU	6	*0.0125*	0.0390	0.0169	0.0151	0.0108	0.64	-
FLUA	6	0.1210	1.7960	0.5825	0.4118	0.6083	1.04	-
I(1,2,3-cd)P	6	0.0720	1.1200	0.3447	0.2370	0.3854	1.12	-
NAF	6	*0.0125*	*0.0125*	*0.0125*	*0.0125*	0.0000	NA	-
PIR	6	0.1040	1.6400	0.5173	0.3582	0.5611	1.08	-
Phenol	6	*0.0050*	*0.0050*	*0.0050*	*0.0050*	0.0000	NA	-

NAF: naphthalene, D(a,h)A: dibenzo(a,h)anthracene, B(g,h,i)P: benzo(g,h,i)perylene, FLU: fluorene, ANT: anthracene, B(a)P: benzo(a)pyrene, I(1,2,3-cd)P: indeno ((1,2,3-cd) pyrene, FEN: phenanthrene, CHR: chrysene, B(b)F: benzo(b)fluoranthene, ACTE: acenaphthene, FLUA: fluoranthene, B(a)A: benzo(a)anthracene, B(k)F: benzo(k)fluoranthene, ACTY: acenaphthylene, PIR: pyrene; ND: not determined; *0.0125*, *0.050*, *0.005*: numbers in italics mean half of the limit of quantification (LOQ) and relate to the content of the substance below LOQ.

**Table 8:** The sum of 16 PAHs in sectors and backyards (mg/kg).

Sector No.	11	12	21	22	23	24	25	31	32	33	34	35	41	42	43	44	45	46	47	51
Sum of 16 PAHs	3.0	6.5	2.8	2.8	2.3	5.8	6.3	4.8	75.5	7.3	23.4	24.7	0.2	0.2	1.0	6.1	3.4	2.5	7.1	4.3
**Backyard No.**	K02	K11	K26	K30	K31	K33														
Sum of 16 PAHs	2.8	2.4	0.9	12.1	2.8	2.1														

Mean content of 16 PAHs in sectors–9.50 mg/kg; mean content of 16 PAHs in backyards–3.85 mg/kg.

**Table 9:** Total hazard indices (HIs) and total cancer risks (CRs) in sectors.

Sector No.	Total HI—Children	Total HI—Adults	Total CR—Aggregate Residents
11	2.0 × 10^−1^	2.4 × 10^−2^	1.4 × 10^−5^
12	3.2 × 10^−1^	3.7 × 10^−2^	2.3 × 10^−5^
21	1.4 × 10^−1^	1.7 × 10^−2^	1.1 × 10^−5^
22	1.9 × 10^−1^	2.2 × 10^−2^	1.2 × 10^−5^
23	2.3 × 10^−1^	2.7 × 10^−2^	1.8 × 10^−5^
24	3.5 × 10^−1^	4.1 × 10^−2^	2.7 × 10^−5^
25	3.2 × 10^−1^	3.6 × 10^−2^	2.2 × 10^−5^
31	2.9 × 10^−2^	3.7 × 10^−3^	5.8 × 10^−6^
32	3.2 × 10^−1^	4.5 × 10^−2^	7.2 × 10^−5^
33	3.8 × 10^−2^	5.0 × 10^−3^	9.0 × 10^−6^
34	1.4 × 10^−1^	1.8 × 10^−2^	3.2 × 10^−5^
35	2.0 × 10^−1^	2.8 × 10^−2^	4.2 × 10^−5^
41	1.0 × 10^−3^	2.2 × 10^−4^	2.8 × 10^−7^
42	1.0 × 10^−3^	2.3 × 10^−4^	2.8 × 10^−7^
43	4.2 × 10^−3^	6.3 × 10^−4^	9.2 × 10^−7^
44	3.5 × 10^−2^	4.5 × 10^−3^	7.9 × 10^−6^
45	2.0 × 10^−2^	2.6 × 10^−3^	4.3 × 10^−6^
46	1.3 × 10^−2^	1.7 × 10^−3^	2.8 × 10^−6^
47	3.8 × 10^−2^	5.1 × 10^−3^	8.8 × 10^−6^
51	2.1 × 10^−2^	2.8 × 10^−3^	4.8 × 10^−6^

**Table 10:** Total hazard indices (HIs) and total cancer risks (CRs) in backyards.

Backyard (Well No.)	Sector No.	Total HI—Children	Total HI—Adults	Total CR—Aggregate Residents
K02	51	1.6 × 10^−2^	2.0 × 10^−3^	3.4 × 10^−6^
K11	44	1.6 × 10^−2^	2.0 × 10^−3^	3.3 × 10^−6^
K26	46	5.1 × 10^−3^	7.1 × 10^−4^	1.2 × 10^−6^
K30	25	4.0 × 10^−1^	4.6 × 10^−2^	3.4 × 10^−5^
K31	23	2.5 × 10^−1^	2.8 × 10^−2^	1.7 × 10^−5^
K33	21	1.9 × 10^−1^	2.2 × 10^−2^	1.2 × 10^−5^

**Table 11:** Health risk-based target levels (HRBTLs).

Substance	HRBTL (mg/kg)	
Metals and metalloids		
Arsenic	6.10	C
Barium	11,000	N
Chromium (III)	37,000	N
Chromium (VI)	1.40	M
Tin	46,000	N
Zinc	23,000	N
Cobalt	23	N
Copper	3000	N
Nickel	920	N
Lead	400	[83,84]
Mercury	17	N
Polycyclic aromatic hydrocarbons (PAHs)		
Acenaphthene	3400	N
Anthracene	17,000	N
Benzo(a)anthracene	11	M
Benzo(a)pyrene	1.1	M
Benzo(b)fluoranthene	11	M
Benzo(k)fluoranthene	110	M
Chrysene	1100	M
Dibenzo(a,h)anthracene	1.1	M
Fluoranthene	2300	N
Fluorene	2300	N
Indeno(1,2,3-cd)pyrene	11	M
Naphthalene	18	C
Pyrene	1700	N
Other substances		
Phenol	18,000	N

N: non-cancer effect; C: cancer effect; M: mutagenic effect.

## Supplementary Materials

The following supporting information can be downloaded at: https://www.mdpi.com/article/10.3390/ijerph19052590/s1: Supplementary Materials 1: Content of analysed contaminants in surface soil (Table S1a,b); Supplementary Materials 2: Health risk assessment results (Tables S1–S78).

## Abbreviations

BRDEP—Bydgoszcz Regional Directorate for Environmental Protection; CP—Chemical Plant; CR—cancer risk; DNT—dinitrotoluene; EPI—epichlorohydrin; EPL—Environmental Protection Law; EU—European Union; HI—hazard index; HQ—hazard quotient; HRA—health risk assessment; HRBTL—health risk-based target level; LOQ—limit of quantification; ŁWS—Łęgnowo-Wieś settlement; PAH—polycyclic aromatic hydrocarbon; PCE—tetrachloroethene; PU—polyurethane; PVC—poly(vinyl chloride); RME—reasonable maximum exposure; TCE—trichloroethene; TDA—toluenediamine; TDI—toluene diisocyanate; TNT—trinitrotoluene; TR_C_—cancer target risk; TR_N_—non-cancer target risk; USEPA—United States Environmental Protection Agency
