# Supplementary material for: Health Risk Assessment for the Residential Area Adjacent to a Former Chemical Plant
Source: Int J Environ Res Public Health. 2022 Feb 23;19(5):2590. doi: 10.3390/ijerph19052590 (PMC8909588; doi:10.3390/ijerph19052590)
Supplement: Supplementary file 1 [file ijerph-19-02590-s001.zip › Supplementary_Material_1_Contaminant_content__Tables_S1a_S1b_11_02_2022.pdf]

Supplementary Material 1 - Content of analysed contaminants in surface soil

Health risk assessment for the residential area adjacent to a former chemical plant

Eleonora Wcisło *, Joachim Bronder

Environmental Health Risk Assessment Group, Departament of Research and Development,
Institute for Ecology of Industrial Areas, 6 Kossutha St., 40-844 Katowice, Poland; j.bronder@ietu.pl (J.B.)
* Correspondence: e.wcislo@ietu.pl; Tel.: +48 32 254 60 31

Table S1a. Content of metals and arsenic in the surface soil of the sectors and selected backyards (mg/kg).

Sector no./ Well no.	As	Ba	Cr	Sn	Zn	Co	Cu	Ni	Pb	Hg
11	2.23	127.00	6.900	*0.50*	95.50	1.96	10.70	5.02	19.50	*0.05*
12	4.37	94.60	8.960	1.00	94.70	2.64	11.80	7.88	25.70	*0.05*
21	1.64	40.20	4.810	*0.50*	54.70	1.28	7.52	3.22	12.00	*0.05*
22	1.87	68.40	5.750	*0.50*	128.00	1.93	8.32	4.90	17.20	*0.05*
23	2.55	64.10	10.800	1.49	68.30	2.47	9.93	6.46	12.50	*0.05*
24	3.12	91.40	14.600	1.40	122.00	3.95	14.20	15.00	20.10	*0.05*
25	3.28	85.20	8.540	*0.50*	156.00	3.12	10.60	8.89	14.20	*0.05*
K30	3.94	125.00	11.300	1.39	162.00	3.30	16.20	10.40	20.00	0.124
K31	3.31	75.20	7.450	*0.50*	58.70	2.30	9.40	6.87	14.50	*0.05*
K33	2.53	46.20	5.260	1.44	73.30	1.85	18.10	4.60	31.00	*0.05*

0.25 – number in italics means half of the limit of quantification (LOQ) and relates to the content of the substance below LOQ.

Table S1b. Content of PAHs, phenol and 3,5-dichlorophenol in the surface soil of the sectors and selected backyards (mg/kg)

Sector no./ Well no.	NAF	ANT	CHR	B(a)A	D(a,h)A	B(a)P	B(b)F	B(k)F	B(g,h,i)P	I(1,2,3-cd)P	ACTE	ACTY	FLU	FEN	FLUA	PIR	Phenol	3,5-Dichloro phenol
11	*0.0125*	0.0500	0.2670	0.2450	*0.0125*	0.2450	0.3400	0.1310	0.1680	0.1970	*0.0125*	*0.0125*	*0.0125*	0.265	0.539	0.439	*0.005*	ND
12	*0.0125*	0.1360	0.5530	0.5050	*0.0125*	0.5380	0.7290	0.2950	0.3850	0.4350	0.046	0.04	0.054	0.659	1.14	0.936	*0.005*	ND
21	*0.0125*	0.0440	0.1800	0.1740	*0.0125*	0.2640	0.3890	0.1480	0.1950	0.2250	*0.0125*	*0.0125*	*0.0125*	0.218	0.497	0.418	*0.005*	*0.0005*
22	*0.0125*	0.0550	0.2360	0.2390	*0.0125*	0.2330	0.3250	0.1270	0.1660	0.1850	*0.0125*	*0.0125*	0.028	0.257	0.505	0.424	*0.005*	*0.0005*
23	*0.0125*	0.0410	0.1980	0.2090	*0.0125*	0.2130	0.2860	0.1080	0.1530	0.1760	*0.0125*	*0.0125*	*0.0125*	0.165	0.375	0.325	0.01	*0.0005*
24	*0.0125*	0.0880	0.5190	0.4760	*0.0125*	0.5440	0.7590	0.2690	0.3960	0.4640	*0.0125*	0.03	0.032	0.415	0.929	0.789	*0.005*	*0.0005*
25	*0.0125*	0.0740	0.5740	0.5240	*0.0125*	0.6080	0.8360	0.3060	0.4810	0.5280	*0.0125*	0.032	*0.0125*	0.362	0.995	0.876	*0.005*	*0.0005*
31	*0.0125*	0.0720	0.2980	0.3290	*0.0125*	0.4720	0.6360	0.2440	0.3270	0.3870	*0.0125*	0.036	*0.0125*	0.33	0.877	0.745	*0.005*	ND
32	0.4910	1.8600	6.7200	6.3800	0.8780	5.0980	7.5100	2.5900	2.8800	3.6600	0.739	0.038	0.972	11.4	14	10.3	*0.005*	ND
33	*0.0125*	0.0750	0.7610	0.7170	0.0950	0.6320	1.1300	0.4050	0.5230	0.5370	*0.0125*	0.031	*0.0125*	0.225	1.08	1.05	*0.005*	ND
34	*0.0125*	0.2870	2.2300	1.3200	0.3690	2.3200	3.3700	1.1990	1.7900	1.8800	0.053	0.076	0.063	1.11	3.97	3.39	*0.005*	ND
35	0.3270	2.9500	3.5100	4.2500	0.4070	3.1200	3.8400	1.4020	1.4500	1.4700	*0.0125*	0.036	*0.0125*	0.33	0.877	0.745	*0.005*	ND
41	*0.0125*	*0.0125*	*0.0125*	*0.0125*	*0.0125*	*0.0125*	*0.0125*	*0.0125*	*0.0125*	*0.0125*	*0.0125*	*0.0125*	*0.0125*	*0.0125*	*0.0125*	*0.0125*	*0.005*	ND
42	*0.0125*	*0.0125*	*0.0125*	*0.0125*	*0.0125*	*0.0125*	*0.0125*	*0.0125*	*0.0125*	*0.0125*	*0.0125*	*0.0125*	*0.0125*	*0.0125*	*0.0125*	*0.0125*	*0.005*	ND
43	*0.0125*	0.0510	0.0720	0.0730	0.0125	0.0650	0.0820	0.0280	0.0360	0.0430	0.025	*0.0125*	0.031	0.174	0.164	0.128	*0.005*	ND
44	*0.0125*	0.0900	0.5430	0.4890	0.0890	0.5750	0.8150	0.2890	0.4690	0.5010	0.034	0.036	0.032	0.389	0.941	0.805	*0.005*	ND
45	*0.0125*	0.0410	0.3120	0.2800	0.0540	0.3160	0.4460	0.1650	0.2280	0.2580	*0.0125*	*0.0125*	*0.0125*	0.201	0.559	0.466	*0.005*	ND
46	*0.0125*	0.0540	0.2150	0.2090	0.0260	0.2080	0.2930	0.1050	0.1410	0.1640	*0.0125*	*0.0125*	*0.0125*	0.218	0.462	0.366	*0.005*	ND
47	*0.0125*	0.0960	0.6470	0.5630	0.1060	0.6390	0.9460	0.3210	0.4850	0.5330	*0.0125*	0.04	0.031	0.456	1.17	0.99	*0.005*	ND
51	*0.0125*	0.0620	0.3910	0.3320	0.0620	0.3470	0.5080	0.1850	0.2410	0.2720	0.035	*0.0125*	0.031	0.392	0.777	0.636	*0.005*	ND
K02	*0.0125*	0.0340	0.2470	0.2410	*0.0125*	0.2590	0.3840	0.1460	0.2150	0.2410	*0.0125*	*0.0125*	*0.0125*	0.15	0.464	0.395	*0.005*	ND
K11	*0.0125*	0.0270	0.2050	0.1970	*0.0125*	0.2580	0.3580	0.1390	0.2190	0.2370	*0.0125*	*0.0125*	*0.0125*	0.1	0.306	0.267	*0.005*	ND
K26	*0.0125*	0.0125	0.0730	0.0680	*0.0125*	0.0820	0.1360	0.0380	0.0610	0.0720	*0.0125*	*0.0125*	*0.0125*	0.05	0.121	0.104	*0.005*	ND
K30	*0.0125*	0.1970	1.1500	0.7230	*0.0125*	1.3400	1.8700	0.6070	0.9660	1.1200	0.043	0.046	0.039	0.517	1.796	1.64	*0.005*	0.001
K31	*0.0125*	0.0510	0.1550	0.2570	*0.0125*	0.2690	0.3740	0.1390	0.2010	0.2330	*0.0125*	*0.0125*	*0.0125*	0.209	0.476	0.415	*0.005*	*0.0005*
K33	*0.0125*	0.0380	0.1680	0.1800	*0.0125*	0.1960	0.2570	0.0950	0.1400	0.1650	*0.0125*	*0.0125*	*0.0125*	0.156	0.332	0.283	*0.005*	*0.0005*

NAF	naphthalene	D(a,h)A	dibenzo(a,h)anthracene	B(g,h,i)P	benzo(g,h,i)perylene	FLU	fluorene
ANT	anthracene	B(a)P	benzo(a)pyrene	I(1,2,3-cd)P	indeno (1,2,3-cd) pyrene	FEN	phenanthrene
CHR	chrysene	B(b)F	benzo(b)fluoranthene	ACTE	acenaphthene	FLUA	fluoranthene
B(a)A	benzo(a)anthracene	B(k)F	benzo(k)fluoranthene	ACTY	acenaphthylene	PIR	pyrene

ND – not determined; *0.0125* - number in italics means half of the limit of quantification (LOQ) and relates to the content of the substance below LOQ
