# Supplementary material for: Health Risk Assessment for the Residential Area Adjacent to a Former Chemical Plant
Source: Int J Environ Res Public Health. 2022 Feb 23;19(5):2590. doi: 10.3390/ijerph19052590 (PMC8909588; doi:10.3390/ijerph19052590)
Supplement: Supplementary file 1 [file ijerph-19-02590-s001.zip › Supplementary_Material_2_HRA_results_Tables_S1¿CS78_11_02_2022.pdf]

Supplementary Material 2 – Health risk assessment results

Health risk assessment for the residential area adjacent to a former chemical plant

Eleonora Wcisło *, Joachim Bronder

Environmental Health Risk Assessment Group, Departament of Research and Development,
Institute for Ecology of Industrial Areas, 6 Kossutha St., 40-844 Katowice, Poland; j.bronder@ietu.pl (J.B.)
* Correspondence: e.wcislo@ietu.pl; Tel.: +48 32 254 60 31

List of Tables

TABLE S1. SECTOR NO. 11 – NON-CANCER RISK – CHILDREN 3
TABLE S2 SECTOR NO. 11 – NON-CANCER RISK – ADULTS.......... 4
TABLE S3. SECTOR NO. 11 – CANCER RISK – AGGREGATE RESIDENTS 5
TABLE S4. SECTOR NO. 12 – NON-CANCER RISK – CHILDREN 6
TABLE S5. SECTOR NO. 12 – NON-CANCER RISK – ADULTS 7
TABLE S6. SECTOR NO. 12 – CANCER RISK – AGGREGATE RESIDENTS 8
TABLE S7. SECTOR NO. 21 – NON-CANCER RISK – CHILDREN 9
TABLE S8. SECTOR NO. 21 – NON-CANCER RISK – ADULTS 10
TABLE S9. SECTOR NO. 21 – CANCER RISK – AGGREGATE RESIDENTS 11
TABLE S10. SECTOR NO. 22 – NON-CANCER RISK – CHILDREN 12
TABLE S11. SECTOR NO. 22 – NON-CANCER RISK – ADULTS 13
TABLE S12. SECTOR NO. 22 – CANCER RISK – AGGREGATE RESIDENTS 14
TABLE S13. SECTOR NO. 23 – NON-CANCER RISK – CHILDREN 15
TABLE S14. SECTOR NO. 23 – NON-CANCER RISK – ADULTS 16
TABLE S15. SECTOR NO. 23 – CANCER RISK – AGGREGATE RESIDENTS 17
TABLE S16. SECTOR NO. 24 – NON-CANCER RISK – CHILDREN 18
TABLE S17. SECTOR NO. 24 – NON-CANCER RISK – ADULTS 19
TABLE S18. SECTOR NO. 24 – CANCER RISK – AGGREGATE RESIDENTS 20
TABLE S19. SECTOR NO. 25 – NON-CANCER RISK – CHILDREN 21
TABLE S20. SECTOR NO. 25 – NON-CANCER RISK – ADULTS 22
TABLE S21. SECTOR NO. 25 – CANCER RISK – AGGREGATE RESIDENTS 23
TABLE S22. SECTOR NO. 31 – NON-CANCER RISK – CHILDREN 24
TABLE S23. SECTOR NO. 31 – NON-CANCER RISK – ADULTS 25
TABLE S24. SECTOR NO. 31 – CANCER RISK – AGGREGATE RESIDENTS 26
TABLE S25. SECTOR NO. 32 – NON-CANCER RISK – CHILDREN 27
TABLE S26. SECTOR NO. 32 – NON-CANCER RISK – ADULTS 28
TABLE S27. SECTOR NO. 32 – CANCER RISK – AGGREGATE RESIDENTS 29
TABLE S28. SECTOR NO. 33 – NON-CANCER RISK – CHILDREN 30
TABLE S29. SECTOR NO. 33 – NON-CANCER RISK – ADULTS 31
TABLE S30. SECTOR NO. 33 – CANCER RISK – AGGREGATE RESIDENTS 32
TABLE S31. SECTOR NO. 34– NON-CANCER RISK – CHILDREN 33
TABLE S32. SECTOR NO. 34 – NON-CANCER RISK – ADULTS 34
TABLE S33. SECTOR NO. 34 – CANCER RISK – AGGREGATE RESIDENTS 35
TABLE S34. SECTOR NO. 35 – NON-CANCER RISK – CHILDREN 36
TABLE S35. SECTOR NO. 35 – NON-CANCER RISK – ADULTS 37
TABLE S36. SECTOR NO. 35 – CANCER RISK – AGGREGATE RESIDENTS 38
TABLE S37. SECTOR NO. 41 – NON-CANCER RISK – CHILDREN 39
TABLE S38. SECTOR NO. 41 – NON-CANCER RISK – ADULTS 40
TABLE S39. SECTOR NO. 41 – CANCER RISK – AGGREGATE RESIDENTS 41
TABLE S40. SECTOR NO. 42– NON-CANCER RISK – CHILDREN 42
TABLE S41. SECTOR NO. 42 – NON-CANCER RISK – ADULTS 43
TABLE S42. SECTOR NO. 42 – CANCER RISK – AGGREGATE RESIDENTS 44
TABLE S43. SECTOR NO. 43 – NON-CANCER RISK – CHILDREN 45
TABLE S44. SECTOR NO. 43 – NON-CANCER RISK – ADULTS 46
TABLE S45. SECTOR NO. 43 – CANCER RISK – AGGREGATE RESIDENTS 47
TABLE S46. SECTOR NO. 44 – NON-CANCER RISK – CHILDREN 48
TABLE S47. SECTOR NO. 44 – NON-CANCER RISK – ADULTS 49
TABLE S48. SECTOR NO. 44 – CANCER RISK – AGGREGATE RESIDENTS 50
TABLE S49. SECTOR NO. 45 – NON-CANCER RISK – CHILDREN 51

Table S50. Sector no. 45 – non-cancer risk – adults 52
Table S51. Sector no. 45 – cancer risk – aggregate residents 53
Table S52. Sector no. 46 – non-cancer risk – children 54
Table S53. Sector no. 46 – non-cancer risk – adults 55
Table S54. Sector no. 46 – cancer risk – aggregate residents 56
Table S55. Sector no. 47 – non-cancer risk – children 57
Table S56. Sector no. 47 – non-cancer risk – adults 58
Table S57. Sector no. 47 – cancer risk – aggregate residents 59
Table S58. Sector no. 51 – non-cancer risk – children 60
Table S59. Sector no. 51 – non-cancer risk – adults 61
Table S60. Sector no. 51 – cancer risk – aggregate residents 62
Table S61. Backyard K02 – non-cancer risk – children 63
Table S62. Backyard K02 – non-cancer risk – adults 64
Table S63. Backyard K02 – cancer risk – aggregate residents 65
Table S64. Backyard K11 – non-cancer risk – children 66
Table S65. Backyard K11 – non-cancer risk – adults 67
Table S66. Backyard K11 – cancer risk – aggregate residents 68
Table S67. Backyard K26 – non-cancer risk – children 69
Table S68. Backyard K26 – non-cancer risk – adults 70
Table S69. Backyard K26 – cancer risk – aggregate residents 71
Table S70. Backyard K30 – non-cancer risk – children 72
Table S71. Backyard K30 – non-cancer risk – adults 73
Table S72. Backyard K30 – cancer risk – aggregate residents 74
Table S73. Backyard K31 – non-cancer risk – children 75
Table S74. Backyard K31 – non-cancer risk – adults 76
Table S75. Backyard K31 – cancer risk – aggregate residents 77
Table S76. Backyard K33 – non-cancer risk – children 78
Table S77. Backyard K33 – non-cancer risk – adults 79
Table S78. Backyard K33 – cancer risk – aggregate residents 80

Table S1. Sector no. 11 – non-cancer risk – children

Substance	Content (mg/kg)	HQo	HQd	HQinh	HI
Acenaphtene	0.01	2.70E-06	9.70E-07	ND	3.60E-06
Anthracene	0.05	2.10E-06	7.80E-07	ND	2.90E-06
Arsenic	2.20	5.70E-02	8.00E-03	1.50E-04	6.50E-02
Barium	130.00	8.10E-03	3.20E-03	2.60E-04	1.20E-02
Benzo(a)pyrene	0.25	1.00E-02	3.80E-03	1.20E-04	1.40E-02
Chromium (III)	5.90	5.00E-05	1.10E-04	ND	1.60E-04
Chromium (VI)	0.99	4.20E-03	4.70E-03	1.00E-05	9.00E-03
Cobalt	2.00	8.40E-02	2.30E-03	3.30E-04	8.60E-02
Copper	11.00	3.40E-03	9.60E-05	ND	3.50E-03
Fluoranthene	0.54	1.70E-04	6.30E-05	ND	2.30E-04
Fluorenee	0.01	4.00E-06	1.50E-06	ND	5.40E-06
Mercury	0.05	2.10E-03	8.50E-04	1.70E-07	3.00E-03
Naphthalene	0.01	8.00E-06	2.90E-06	2.60E-04	2.70E-04
Nickel	5.00	3.20E-03	2.20E-03	5.60E-05	5.50E-03
Phenol	0.01	2.10E-07	6.00E-08	2.50E-11	2.70E-07
Pyrene	0.44	1.90E-04	6.80E-05	ND	2.60E-04
Tin	0.50	1.10E-05	3.00E-07	ND	1.10E-05
Zinc	96.00	4.10E-03	1.10E-04	ND	4.20E-03
Totals		1.80E-01	2.60E-02	1.20E-03	2.00E-01

Table S2 Sector no. 11 – non-cancer risk – adults

Substance	Content (mg/kg)	HQo	HQd	HQinh	HI
Acenaphtene	0.01	2.90E-07	1.50E-07	ND	4.30E-07
Anthracene	0.05	2.30E-07	1.20E-07	ND	3.50E-07
Arsenic	2.20	6.10E-03	1.20E-03	1.50E-04	7.50E-03
Barium	130.00	8.70E-04	5.00E-04	2.60E-04	1.60E-03
Benzo(a)pyrene	0.25	1.10E-03	5.80E-04	1.20E-04	1.80E-03
Chromium (III)	5.90	5.40E-06	1.70E-05	ND	2.20E-05
Chromium (VI)	0.99	4.50E-04	7.20E-04	1.00E-05	1.20E-03
Cobalt	2.00	8.90E-03	3.60E-04	3.30E-04	9.60E-03
Copper	11.00	3.70E-04	1.50E-05	ND	3.80E-04
Fluoranthene	0.54	1.80E-05	9.60E-06	ND	2.80E-05
Fluorene	0.01	4.30E-07	2.20E-07	ND	6.50E-07
Mercury	0.05	2.30E-04	1.30E-04	1.70E-07	3.60E-04
Naphthalene	0.01	8.60E-07	4.40E-07	1.30E-04	1.30E-04
Nickel	5.00	3.40E-04	3.40E-04	5.60E-05	7.40E-04
Phenol	0.01	2.30E-08	9.10E-09	2.50E-11	3.20E-08
Pyrene	0.44	2.00E-05	1.00E-05	ND	3.00E-05
Tin	0.50	1.10E-06	4.60E-08	ND	1.20E-06
Zinc	96.00	4.40E-04	1.70E-05	ND	4.50E-04
Totals		1.90E-02	4.00E-03	1.10E-03	2.40E-02

Table S3. Sector no. 11 – cancer risk – aggregate residents

Substance	Content (mg/kg)	CRo	CRd	CRinh	CR
Arsenic	2.20	3.10E-06	5.00E-07	4.10E-09	3.60E-06
Benzo(a)anthracene	0.25	1.60E-07	6.30E-08	7.40E-09	2.30E-07
Benzo(a)pyrene	0.25	1.60E-06	6.30E-07	1.60E-10	2.30E-06
Benzo(b)fluoranthene	0.34	2.30E-07	8.80E-08	2.20E-11	3.20E-07
Benzo(k)fluoranthene	0.13	8.80E-09	3.40E-09	8.60E-13	1.20E-08
Chromium (VI)	0.99	3.30E-06	3.90E-06	9.10E-08	7.30E-06
Chrysene	0.27	1.80E-09	6.90E-10	1.80E-13	2.50E-09
Cobalt	2.00	ND	ND	7.60E-09	7.60E-09
Dibenzo(a,h)anthracene	0.01	8.40E-08	3.20E-08	8.20E-12	1.20E-07
Indeno(1,2,3-cd)pyrene	0.20	1.30E-07	5.10E-08	1.30E-11	1.80E-07
Naphthalene	0.01	ND	ND	6.80E-09	6.80E-09
Nickel	5.00	ND	ND	5.60E-10	5.60E-10
Totals		8.60E-06	5.30E-06	1.20E-07	1.40E-05

Table S4. Sector no. 12 – non-cancer risk – children

Substance	Content (mg/kg)	HQo	HQd	HQinh	HI
Acenaphtene	0.05	9.80E-06	3.60E-06	ND	1.30E-05
Anthracene	0.14	5.80E-06	2.10E-06	ND	7.90E-06
Arsenic	4.40	1.10E-01	1.60E-02	3.00E-04	1.30E-01
Barium	95.00	6.00E-03	2.40E-03	2.00E-04	8.70E-03
Benzo(a)pyrene	0.54	2.30E-02	8.30E-03	2.80E-04	3.20E-02
Chromium (III)	7.70	6.50E-05	1.40E-04	ND	2.10E-04
Chromium (VI)	1.30	5.50E-03	6.10E-03	1.30E-05	1.20E-02
Cobalt	2.60	1.10E-01	3.20E-03	4.60E-04	1.20E-01
Copper	12.00	3.80E-03	1.10E-04	ND	3.90E-03
Fluoranthene	1.10	3.60E-04	1.30E-04	ND	5.00E-04
Fluorene	0.05	1.70E-05	6.30E-06	ND	2.40E-05
Mercury	0.05	2.10E-03	8.50E-04	1.70E-07	3.00E-03
Naphthalene	0.01	8.00E-06	2.90E-06	2.70E-04	2.80E-04
Nickel	7.90	5.00E-03	3.50E-03	9.10E-05	8.70E-03
Phenol	0.01	2.10E-07	6.00E-08	2.60E-11	2.70E-07
Pyrene	0.94	4.00E-04	1.50E-04	ND	5.40E-04
Tin	1.00	2.10E-05	6.00E-07	ND	2.20E-05
Zinc	95.00	4.00E-03	1.10E-04	ND	4.10E-03
Totals		2.70E-01	4.10E-02	1.60E-03	3.2 0E-01

Table S5. Sector no. 12 – non-cancer risk – adults

Substance	Content (mg/kg)	HQo	HQd	HQinh	HI
Acenaphtene	0.05	1.10E-06	5.40E-07	ND	1.60E-06
Anthracene	0.14	6.20E-07	3.20E-07	ND	9.40E-07
Arsenic	4.40	1.20E-02	2.40E-03	3.00E-04	1.50E-02
Barium	95.00	6.50E-04	3.70E-04	2.00E-04	1.20E-03
Benzo(a)pyrene	0.54	2.50E-03	1.30E-03	2.80E-04	4.00E-03
Chromium (III)	7.70	7.00E-06	2.20E-05	ND	2.90E-05
Chromium (VI)	1.30	5.80E-04	9.30E-04	1.30E-05	1.50E-03
Cobalt	2.60	1.20E-02	4.80E-04	4.60E-04	1.30E-02
Copper	12.00	4.00E-04	1.60E-05	ND	4.20E-04
Fluoranthene	1.10	3.90E-05	2.00E-05	ND	5.90E-05
Fluorene	0.05	1.80E-06	9.60E-07	ND	2.80E-06
Mercury	0.05	2.30E-04	1.30E-04	1.70E-07	3.60E-04
Naphthalene	0.01	8.60E-07	4.40E-07	1.30E-04	1.40E-04
Nickel	7.90	5.40E-04	5.40E-04	9.10E-05	1.20E-03
Phenol	0.01	2.30E-08	9.10E-09	2.60E-11	3.20E-08
Pyrene	0.94	4.30E-05	2.20E-05	ND	6.50E-05
Tin	1.00	2.30E-06	9.10E-08	ND	2.40E-06
Zinc	95.00	4.30E-04	1.70E-05	ND	4.50E-04
Totals		3.00E-02	6.30E-03	1.50E-03	3.70E-02

Table S6. Sector no. 12 – cancer risk – aggregate residents

Substance	Content (mg/kg)	CRo	CRd	CRinh	CR
Arsenic	4.40	6.20E-06	9.70E-07	8.40E-09	7.10E-06
Benzo(a)anthracene	0.51	3.40E-07	1.30E-07	1.60E-08	4.80E-07
Benzo(a)pyrene	0.54	3.60E-06	1.40E-06	3.60E-10	5.00E-06
Benzo(b)fluoranthene	0.73	4.90E-07	1.90E-07	4.90E-11	6.80E-07
Benzo(k)fluoranthene	0.30	2.00E-08	7.60E-09	2.00E-12	2.70E-08
Chromium (VI)	1.30	4.30E-06	5.10E-06	1.20E-07	9.50E-06
Chrysene	0.55	3.70E-09	1.40E-09	3.80E-13	5.10E-09
Cobalt	2.60	ND	ND	1.10E-08	1.10E-08
Dibenzo(a,h)anthracene	0.01	8.40E-08	3.20E-08	8.50E-12	1.20E-07
Indeno(1,2,3-cd)pyrene	0.44	2.90E-07	1.10E-07	2.90E-11	4.00E-07
Naphthalene	0.01	ND	ND	7.00E-09	7.00E-09
Nickel	7.90	ND	ND	9.10E-10	9.10E-10
Totals		1.50E-05	7.90E-06	1.60E-07	2.30E-05

Table S7. Sector no. 21 – non-cancer risk – children

Substance	Content (mg/kg)	HQo	HQd	HQinh	HI
Acenaphtene	0.01	2.70E-06	9.70E-07	ND	3.60E-06
Anthracene	0.04	1.90E-06	6.80E-07	ND	2.60E-06
Arsenic	1.60	4.20E-02	5.90E-03	9.60E-05	4.80E-02
Barium	40.00	2.60E-03	1.00E-03	7.10E-05	3.70E-03
Benzo(a)pyrene	0.26	1.10E-02	4.10E-03	1.20E-04	1.50E-02
Chromium (III)	4.10	3.50E-05	7.60E-05	ND	1.10E-04
Chromium (VI)	0.69	2.90E-03	3.30E-03	6.10E-06	6.20E-03
Cobalt	1.30	5.50E-02	1.50E-03	1.90E-04	5.60E-02
Copper	7.50	2.40E-03	6.70E-05	ND	2.50E-03
Fluoranthene	0.50	1.60E-04	5.80E-05	ND	2.20E-04
Fluorene	0.01	4.00E-06	1.50E-06	ND	5.40E-06
Mercury	0.05	2.10E-03	8.50E-04	1.50E-07	3.00E-03
Naphthalene	0.01	8.00E-06	2.90E-06	2.30E-04	2.40E-04
Nickel	3.20	2.10E-03	1.40E-03	3.10E-05	3.50E-03
Phenol	0.01	2.10E-07	6.00E-08	2.20E-11	2.70E-07
Pyrene	0.42	1.80E-04	6.50E-05	ND	2.40E-04
Tin	0.50	1.10E-05	3.00E-07	ND	1.10E-05
Zinc	55.00	2.30E-03	6.50E-05	ND	2.40E-03
Totals		1.30E-01	1.90E-02	7.70E-04	1.40E-01

Table S8. Sector no. 21 – non-cancer risk – adults

Substance	Content (mg/kg)	HQo	HQd	HQinh	HI
Acenaphtene	0.01	2.90E-07	1.50E-07	ND	4.30E-07
Anthracene	0.04	2.00E-07	1.00E-07	ND	3.10E-07
Arsenic	1.60	4.50E-03	9.00E-04	9.60E-05	5.50E-03
Barium	40.00	2.80E-04	1.60E-04	7.10E-05	5.00E-04
Benzo(a)pyrene	0.26	1.20E-03	6.30E-04	1.20E-04	1.90E-03
Chromium (III)	4.10	3.80E-06	1.20E-05	ND	1.50E-05
Chromium (VI)	0.69	3.20E-04	5.00E-04	6.10E-06	8.20E-04
Cobalt	1.30	5.80E-03	2.30E-04	1.90E-04	6.30E-03
Copper	7.50	2.60E-04	1.00E-05	ND	2.70E-04
Fluoranthene	0.50	1.70E-05	8.80E-06	ND	2.60E-05
Fluorene	0.01	4.30E-07	2.20E-07	ND	6.50E-07
Mercury	0.05	2.30E-04	1.30E-04	1.50E-07	3.60E-04
Naphthalene	0.01	8.60E-07	4.40E-07	1.10E-04	1.10E-04
Nickel	3.20	2.20E-04	2.20E-04	3.10E-05	4.70E-04
Phenol	0.01	2.30E-08	9.10E-09	2.20E-11	3.20E-08
Pyrene	0.42	1.90E-05	9.90E-06	ND	2.90E-05
Tin	0.50	1.10E-06	4.60E-08	ND	1.20E-06
Zinc	55.00	2.50E-04	1.00E-05	ND	2.60E-04
Totals		1.40E-02	2.90E-03	6.50E-04	1.70E-02

Table S9. Sector no. 21 – cancer risk – aggregate residents

Substance	Content (mg/kg)	CRo	CRd	CRinh	CR
Arsenic	1.60	2.30E-06	3.60E-07	2.70E-09	2.70E-06
Benzo(a)anthracene	0.17	1.20E-07	4.50E-08	4.60E-09	1.70E-07
Benzo(a)pyrene	0.26	1.80E-06	6.80E-07	1.50E-10	2.50E-06
Benzo(b)fluoranthene	0.39	2.60E-07	1.00E-07	2.20E-11	3.60E-07
Benzo(k)fluoranthene	0.15	9.90E-09	3.80E-09	8.50E-13	1.40E-08
Chromium (VI)	0.69	2.30E-06	2.70E-06	5.50E-08	5.10E-06
Chrysene	0.18	1.20E-09	4.60E-10	1.00E-13	1.70E-09
Cobalt	1.30	ND	ND	4.30E-09	4.30E-09
Dibenzo(a,h)anthracene	0.01	8.40E-08	3.20E-08	7.20E-12	1.20E-07
Indeno(1,2,3-cd)pyrene	0.23	1.50E-07	5.80E-08	1.30E-11	2.10E-07
Naphthalene	0.01	ND	ND	5.90E-09	5.90E-09
Nickel	3.20	ND	ND	3.20E-10	3.20E-10
Totals		7.00E-06	4.00E-06	7.30E-08	1.10E-05

Table S10. Sector no. 22 – non-cancer risk – children

Substance	Content (mg/kg)	HQo	HQd	HQinh	HI
Acenaphtene	0.01	2.70E-06	9.70E-07	ND	3.60E-06
Anthracene	0.06	2.30E-06	8.50E-07	ND	3.20E-06
Arsenic	1.90	4.80E-02	6.70E-03	1.20E-04	5.50E-02
Barium	68.00	4.40E-03	1.70E-03	1.40E-04	6.30E-03
Benzo(a)pyrene	0.23	9.90E-03	3.60E-03	1.20E-04	1.40E-02
Chromium (III)	4.90	4.20E-05	9.10E-05	ND	1.30E-04
Chromium (VI)	0.82	3.50E-03	3.90E-03	8.20E-06	7.40E-03
Cobalt	1.90	8.20E-02	2.30E-03	3.20E-04	8.50E-02
Copper	8.30	2.70E-03	7.40E-05	ND	2.70E-03
Fluoranthene	0.51	1.60E-04	5.90E-05	ND	2.20E-04
Fluorene	0.03	8.90E-06	3.30E-06	ND	1.20E-05
Mercury	0.05	2.10E-03	8.50E-04	1.70E-07	3.00E-03
Naphthalene	0.01	8.00E-06	2.90E-06	2.60E-04	2.70E-04
Nickel	4.90	3.10E-03	2.20E-03	5.40E-05	5.40E-03
Phenol	0.01	2.10E-07	6.00E-08	2.50E-11	2.70E-07
Pyrene	0.42	1.80E-04	6.60E-05	ND	2.50E-04
Tin	0.50	1.10E-05	3.00E-07	ND	1.10E-05
Zinc	130.00	5.50E-03	1.50E-04	ND	5.60E-03
Totals		1.70E-01	2.20E-02	1.00E-03	1.90E-01

Table S11. Sector no. 22 – non-cancer risk – adults

Substance	Content (mg/kg)	HQo	HQd	HQinh	HI
Acenaphtene	0.01	2.90E-07	1.50E-07	ND	4.30E-07
Anthracene	0.06	2.50E-07	1.30E-07	ND	3.80E-07
Arsenic	1.90	5.10E-03	1.00E-03	1.20E-04	6.30E-03
Barium	68.00	4.70E-04	2.70E-04	1.40E-04	8.70E-04
Benzo(a)pyrene	0.23	1.10E-03	5.50E-04	1.20E-04	1.70E-03
Chromium (III)	4.90	4.50E-06	1.40E-05	ND	1.80E-05
Chromium (VI)	0.82	3.70E-04	6.00E-04	8.20E-06	9.80E-04
Cobalt	1.90	8.80E-03	3.50E-04	3.20E-04	9.50E-03
Copper	8.30	2.80E-04	1.10E-05	ND	3.00E-04
Fluoranthene	0.51	1.70E-05	9.00E-06	ND	2.60E-05
Fluorene	0.03	9.60E-07	5.00E-07	ND	1.50E-06
Mercury	0.05	2.30E-04	1.30E-04	1.70E-07	3.60E-04
Naphthalene	0.01	8.60E-07	4.40E-07	1.30E-04	1.30E-04
Nickel	4.90	3.40E-04	3.30E-04	5.40E-05	7.20E-04
Phenol	0.01	2.30E-08	9.10E-09	2.50E-11	3.20E-08
Pyrene	0.42	1.90E-05	1.00E-05	ND	2.90E-05
Tin	0.50	1.10E-06	4.60E-08	ND	1.20E-06
Zinc	130.00	5.80E-04	2.30E-05	ND	6.10E-04
Totals		1.80E-02	3.40E-03	9.20E-04	2.20E-02

Table S12. Sector no. 22 – cancer risk – aggregate residents

Substance	Content (mg/kg)	CRo	CRd	CRinh	CR
Arsenic	1.90	2.60E-06	4.20E-07	3.40E-09	3.10E-06
Benzo(a)anthracene	0.24	1.60E-07	6.20E-08	7.10E-09	2.30E-07
Benzo(a)pyrene	0.23	1.60E-06	6.00E-07	1.50E-10	2.20E-06
Benzo(b)fluoranthene	0.33	2.20E-07	8.40E-08	2.10E-11	3.00E-07
Benzo(k)fluoranthene	0.13	8.50E-09	3.30E-09	8.20E-13	1.20E-08
Chromium (VI)	0.82	2.70E-06	3.20E-06	7.50E-08	6.10E-06
Chrysene	0.24	1.60E-09	6.10E-10	1.50E-13	2.20E-09
Cobalt	1.90	ND	ND	7.40E-09	7.40E-09
Dibenzo(a,h)anthracene	0.01	8.40E-08	3.20E-08	8.10E-12	1.20E-07
Indeno(1,2,3-cd)pyrene	0.19	1.20E-07	4.80E-08	1.20E-11	1.70E-07
Naphthalene	0.01	ND	ND	6.70E-09	6.70E-09
Nickel	4.90	ND	ND	5.40E-10	5.40E-10
Totals		7.50E-06	4.40E-06	1.00E-07	1.20E-05

Table S13. Sector no. 23 – non-cancer risk – children

Substance	Content (mg/kg)	HQo	HQd	HQinh	HI
Acenaphtene	0.01	2.70E-06	9.70E-07	ND	3.60E-06
Anthracene	0.04	1.70E-06	6.40E-07	ND	2.40E-06
Arsenic	2.60	6.50E-02	9.10E-03	1.70E-04	7.40E-02
Barium	64.00	4.10E-03	1.60E-03	1.20E-04	5.90E-03
Benzo(a)pyrene	0.21	9.10E-03	3.30E-03	1.00E-04	1.20E-02
Chromium (III)	9.30	7.90E-05	1.70E-04	ND	2.50E-04
Chromium (VI)	1.50	6.60E-03	7.40E-03	1.50E-05	1.40E-02
Cobalt	2.50	1.10E-01	2.90E-03	4.00E-04	1.10E-01
Copper	9.90	3.20E-03	8.90E-05	ND	3.30E-03
Fluoranthene	0.38	1.20E-04	4.40E-05	ND	1.60E-04
Fluorene	0.01	4.00E-06	1.50E-06	ND	5.40E-06
Mercury	0.05	2.10E-03	8.50E-04	1.60E-07	3.00E-03
Naphthalene	0.01	8.00E-06	2.90E-06	2.50E-04	2.60E-04
Nickel	6.50	4.10E-03	2.90E-03	7.00E-05	7.10E-03
Phenol	0.01	4.30E-07	1.20E-07	4.90E-11	5.50E-07
Pyrene	0.33	1.40E-04	5.00E-05	ND	1.90E-04
Tin	1.50	3.20E-05	8.90E-07	ND	3.30E-05
Zinc	68.00	2.90E-03	8.20E-05	ND	3.00E-03
Totals		2.10E-01	2.90E-02	1.10E-03	2.30E-01

Table S14. Sector no. 23 – non-cancer risk – adults

Substance	Content (mg/kg)	HQo	HQd	HQinh	HI
Acenaphtene	0.01	2.90E-07	1.50E-07	ND	4.30E-07
Anthracene	0.04	1.90E-07	9.70E-08	ND	2.80E-07
Arsenic	2.60	7.00E-03	1.40E-03	1.70E-04	8.50E-03
Barium	64.00	4.40E-04	2.50E-04	1.20E-04	8.10E-04
Benzo(a)pyrene	0.21	9.70E-04	5.00E-04	1.00E-04	1.60E-03
Chromium (III)	9.30	8.50E-06	2.60E-05	ND	3.40E-05
Chromium (VI)	1.50	7.00E-04	1.10E-03	1.50E-05	1.80E-03
Cobalt	2.50	1.10E-02	4.50E-04	4.00E-04	1.20E-02
Copper	9.90	3.40E-04	1.40E-05	ND	3.50E-04
Fluoranthene	0.38	1.30E-05	6.70E-06	ND	2.00E-05
Fluorene	0.01	4.30E-07	2.20E-07	ND	6.50E-07
Mercury	0.05	2.30E-04	1.30E-04	1.60E-07	3.60E-04
Naphthalene	0.01	8.60E-07	4.40E-07	1.30E-04	1.30E-04
Nickel	6.50	4.40E-04	4.40E-04	7.00E-05	9.50E-04
Phenol	0.01	4.60E-08	1.80E-08	4.90E-11	6.40E-08
Pyrene	0.33	1.50E-05	7.70E-06	ND	2.30E-05
Tin	1.50	3.40E-06	1.40E-07	ND	3.50E-06
Zinc	68.00	3.10E-04	1.20E-05	ND	3.20E-04
Totals		2.20E-02	4.40E-03	1.00E-03	2.70E-02

Table S15. Sector no. 23 – cancer risk – aggregate residents

Substance	Content (mg/kg)	CRo	CRd	CRinh	CR
Arsenic	2.60	3.60E-06	5.70E-07	4.60E-09	4.20E-06
Benzo(a)anthracene	0.21	1.40E-07	5.40E-08	6.10E-09	2.00E-07
Benzo(a)pyrene	0.21	1.40E-06	5.50E-07	1.30E-10	2.00E-06
Benzo(b)fluoranthene	0.29	1.90E-07	7.40E-08	1.80E-11	2.70E-07
Benzo(k)fluoranthene	0.11	7.20E-09	2.80E-09	6.80E-13	1.00E-08
Chromium (VI)	1.50	5.20E-06	6.10E-06	1.40E-07	1.10E-05
Chrysene	0.20	1.30E-09	5.10E-10	1.30E-13	1.80E-09
Cobalt	2.50	ND	ND	9.30E-09	9.30E-09
Dibenzo(a,h)anthracene	0.01	8.40E-08	3.20E-08	7.90E-12	1.20E-07
Indeno(1,2,3-cd)pyrene	0.18	1.20E-07	4.50E-08	1.10E-11	1.60E-07
Naphthalene	0.01	ND	ND	6.60E-09	6.60E-09
Nickel	6.50	ND	ND	7.00E-10	7.00E-10
Totals		1.10E-05	7.40E-06	1.70E-07	1.80E-05

Table S16. Sector no. 24 – non-cancer risk – children

Substance	Content (mg/kg)	HQo	HQd	HQinh	HI
Acenaphtene	0.01	2.70E-06	9.70E-07	ND	3.60E-06
Anthracene	0.09	3.80E-06	1.40E-06	ND	5.10E-06
Arsenic	3.10	8.00E-02	1.10E-02	2.10E-04	9.10E-02
Barium	91.00	5.80E-03	2.30E-03	1.90E-04	8.40E-03
Benzo(a)pyrene	0.54	2.30E-02	8.40E-03	2.80E-04	3.20E-02
Chromium (III)	13.00	1.10E-04	2.30E-04	ND	3.40E-04
Chromium (VI)	2.10	8.90E-03	1.00E-02	2.20E-05	1.90E-02
Cobalt	4.00	1.70E-01	4.70E-03	6.80E-04	1.70E-01
Copper	14.00	4.50E-03	1.30E-04	ND	4.70E-03
Fluoranthene	0.93	3.00E-04	1.10E-04	ND	4.10E-04
Fluorene	0.03	1.00E-05	3.70E-06	ND	1.40E-05
Mercury	0.05	2.10E-03	8.50E-04	1.70E-07	3.00E-03
Naphthalene	0.01	8.00E-06	2.90E-06	2.70E-04	2.80E-04
Nickel	15.00	9.60E-03	6.70E-03	1.70E-04	1.60E-02
Phenol	0.01	2.10E-07	6.00E-08	2.60E-11	2.70E-07
Pyrene	0.79	3.40E-04	1.20E-04	ND	4.60E-04
Tin	1.40	3.00E-05	8.40E-07	ND	3.10E-05
Zinc	120.00	5.20E-03	1.50E-04	ND	5.30E-03
Totals		3.10E-01	4.50E-02	1.80E-03	3.50E-01

Table S17. Sector no. 24 – non-cancer risk – adults

Substance	Content (mg/kg)	HQo	HQd	HQinh	HI
Acenaphtene	0.01	2.90E-07	1.50E-07	ND	4.30E-07
Anthracene	0.09	4.00E-07	2.10E-07	ND	6.10E-07
Arsenic	3.10	8.50E-03	1.70E-03	2.10E-04	1.00E-02
Barium	91.00	6.30E-04	3.60E-04	1.90E-04	1.20E-03
Benzo(a)pyrene	0.54	2.50E-03	1.30E-03	2.80E-04	4.10E-03
Chromium (III)	13.00	1.10E-05	3.50E-05	ND	4.60E-05
Chromium (VI)	2.10	9.50E-04	1.50E-03	2.20E-05	2.50E-03
Cobalt	4.00	1.80E-02	7.20E-04	6.80E-04	1.90E-02
Copper	14.00	4.90E-04	1.90E-05	ND	5.10E-04
Fluoranthene	0.93	3.20E-05	1.70E-05	ND	4.80E-05
Fluorene	0.03	1.10E-06	5.70E-07	ND	1.70E-06
Mercury	0.05	2.30E-04	1.30E-04	1.70E-07	3.60E-04
Naphthalene	0.01	8.60E-07	4.40E-07	1.30E-04	1.30E-04
Nickel	15.00	1.00E-03	1.00E-03	1.70E-04	2.20E-03
Phenol	0.01	2.30E-08	9.10E-09	2.60E-11	3.20E-08
Pyrene	0.79	3.60E-05	1.90E-05	ND	5.50E-05
Tin	1.40	3.20E-06	1.30E-07	ND	3.30E-06
Zinc	120.00	5.60E-04	2.20E-05	ND	5.80E-04
Totals		3.30E-02	6.90E-03	1.70E-03	4.10E-02

Table S18. Sector no. 24 – cancer risk – aggregate residents

Substance	Content (mg/kg)	CRo	CRd	CRinh	CR
Arsenic	3.10	4.40E-06	6.90E-07	5.90E-09	5.10E-06
Benzo(a)anthracene	0.48	3.20E-07	1.20E-07	1.50E-08	4.60E-07
Benzo(a)pyrene	0.54	3.60E-06	1.40E-06	3.70E-10	5.00E-06
Benzo(b)fluoranthene	0.76	5.10E-07	2.00E-07	5.10E-11	7.00E-07
Benzo(k)fluoranthene	0.27	1.80E-08	6.90E-09	1.80E-12	2.50E-08
Chromium (VI)	2.10	7.00E-06	8.30E-06	2.00E-07	1.50E-05
Chrysene	0.52	3.50E-09	1.30E-09	3.50E-13	4.80E-09
Cobalt	4.00	ND	ND	1.60E-08	1.60E-08
Dibenzo(a,h)anthracene	0.01	8.40E-08	3.20E-08	8.40E-12	1.20E-07
Indeno(1,2,3-cd)pyrene	0.46	3.10E-07	1.20E-07	3.10E-11	4.30E-07
Naphthalene	0.01	ND	ND	7.00E-09	7.00E-09
Nickel	15.00	ND	ND	1.70E-09	1.70E-09
Totals		1.60E-05	1.10E-05	2.50E-07	2.70E-05

Table S19. Sector no. 25 – non-cancer risk – children

Substance	Content (mg/kg)	HQo	HQd	HQinh	HI
Acenaphtene	0.01	2.70E-06	9.70E-07	ND	3.60E-06
Anthracene	0.07	3.20E-06	1.10E-06	ND	4.30E-06
Arsenic	3.30	8.40E-02	1.20E-02	2.20E-04	9.60E-02
Barium	85.00	5.40E-03	2.20E-03	1.70E-04	7.80E-03
Benzo(a)pyrene	0.61	2.60E-02	9.40E-03	3.10E-04	3.60E-02
Chromium (III)	7.30	6.20E-05	1.30E-04	ND	2.00E-04
Chromium (VI)	1.20	5.20E-03	5.80E-03	1.20E-05	1.10E-02
Cobalt	3.10	1.30E-01	3.70E-03	5.30E-04	1.40E-01
Copper	11.00	3.40E-03	9.50E-05	ND	3.50E-03
Fluoranthene	1.00	3.20E-04	1.20E-04	ND	4.30E-04
Fluorene	0.01	4.00E-06	1.50E-06	ND	5.40E-06
Mercury	0.05	2.10E-03	8.50E-04	1.70E-07	3.00E-03
Naphthalene	0.01	8.00E-06	2.90E-06	2.60E-04	2.70E-04
Nickel	8.90	5.70E-03	4.00E-03	1.00E-04	9.80E-03
Phenol	0.01	2.10E-07	6.00E-08	2.60E-11	2.70E-07
Pyrene	0.88	3.70E-04	1.40E-04	ND	5.10E-04
Tin	0.50	1.10E-05	3.00E-07	ND	1.10E-05
Zinc	160.00	6.60E-03	1.90E-04	ND	6.80E-03
Totals		2.70E-01	3.90E-02	1.60E-03	3.20E-01

Table S20. Sector no. 25 – non-cancer risk – adults

Substance	Content (mg/kg)	HQo	HQd	HQinh	HI
Acenaphtene	0.01	2.90E-07	1.50E-07	ND	4.30E-07
Anthracene	0.07	3.40E-07	1.80E-07	ND	5.10E-07
Arsenic	3.30	9.00E-03	1.80E-03	2.20E-04	1.10E-02
Barium	85.00	5.80E-04	3.30E-04	1.70E-04	1.10E-03
Benzo(a)pyrene	0.61	2.80E-03	1.40E-03	3.10E-04	4.50E-03
Chromium (III)	7.30	6.70E-06	2.10E-05	ND	2.70E-05
Chromium (VI)	1.20	5.60E-04	8.90E-04	1.20E-05	1.50E-03
Cobalt	3.10	1.40E-02	5.70E-04	5.30E-04	1.50E-02
Copper	11.00	3.60E-04	1.40E-05	ND	3.80E-04
Fluoranthene	1.00	3.40E-05	1.80E-05	ND	5.20E-05
Fluorene	0.01	4.30E-07	2.20E-07	ND	6.50E-07
Mercury	0.05	2.30E-04	1.30E-04	1.70E-07	3.60E-04
Naphthalene	0.01	8.60E-07	4.40E-07	1.30E-04	1.30E-04
Nickel	8.90	6.10E-04	6.10E-04	1.00E-04	1.30E-03
Phenol	0.01	2.30E-08	9.10E-09	2.60E-11	3.20E-08
Pyrene	0.88	4.00E-05	2.10E-05	ND	6.10E-05
Tin	0.50	1.10E-06	4.60E-08	ND	1.20E-06
Zinc	160.00	7.10E-04	2.80E-05	ND	7.40E-04
Totals		2.90E-02	5.90E-03	1.50E-03	3.60E-02

Table S21. Sector no. 25 – cancer risk – aggregate residents

Substance	Content (mg/kg)	CRo	CRd	CRinh	CR
Arsenic	3.30	4.60E-06	7.30E-07	6.20E-09	5.40E-06
Benzo(a)anthracene	0.52	3.50E-07	1.30E-07	1.60E-08	5.00E-07
Benzo(a)pyrene	0.61	4.10E-06	1.60E-06	4.10E-10	5.60E-06
Benzo(b)fluoranthene	0.84	5.60E-07	2.20E-07	5.60E-11	7.80E-07
Benzo(k)fluoranthene	0.31	2.10E-08	7.90E-09	2.00E-12	2.80E-08
Chromium (VI)	1.20	4.10E-06	4.80E-06	1.10E-07	9.00E-06
Chrysene	0.57	3.80E-09	1.50E-09	3.80E-13	5.30E-09
Cobalt	3.10	ND	ND	1.20E-08	1.20E-08
Dibenzo(a,h)anthracene	0.01	8.40E-08	3.20E-08	8.30E-12	1.20E-07
Indeno(1,2,3-cd)pyrene	0.53	3.50E-07	1.40E-07	3.50E-11	4.90E-07
Naphthalene	0.01	ND	ND	6.90E-09	6.90E-09
Nickel	8.90	ND	ND	1.00E-09	1.00E-09
Totals		1.40E-05	7.70E-06	1.50E-07	2.20E-05

Table S22. Sector no. 31 – non-cancer risk – children

Substance	Content (mg/kg)	HQo	HQd	HQinh	HI
Acenaphtene	0.01	2.70E-06	9.70E-07	ND	3.60E-06
Anthracene	0.07	3.10E-06	1.10E-06	ND	4.20E-06
Arsenic	ND	ND	ND	ND	ND
Barium	ND	ND	ND	ND	ND
Benzo(a)pyrene	0.47	2.00E-02	7.30E-03	2.00E-04	2.80E-02
Cadmium	ND	ND	ND	ND	ND
Chromium (III)	ND	ND	ND	ND	ND
Chromium (VI)	ND	ND	ND	ND	ND
Cobalt	ND	ND	ND	ND	ND
Copper	ND	ND	ND	ND	ND
Fluoranthene	0.88	2.80E-04	1.00E-04	ND	3.80E-04
Fluorene	0.01	4.00E-06	1.50E-06	ND	5.40E-06
Mercury	ND	ND	ND	ND	ND
Molybdenum	ND	ND	ND	ND	ND
Naphthalene	0.01	8.00E-06	2.90E-06	2.10E-04	2.20E-04
Nickel	ND	ND	ND	ND	ND
Phenol	0.01	2.10E-07	6.00E-08	2.10E-11	2.70E-07
Pyrene	0.75	3.20E-04	1.20E-04	ND	4.30E-04
Tin	ND	ND	ND	ND	ND
Zinc	ND	ND	ND	ND	ND
Totals		2.10E-02	7.50E-03	4.10E-04	2.90E-02

Table S23. Sector no. 31 – non-cancer risk – adults

Substance	Content (mg/kg)	HQo	HQd	HQinh	HI
Acenaphtene	0.01	2.90E-07	1.50E-07	ND	4.30E-07
Anthracene	0.07	3.30E-07	1.70E-07	ND	5.00E-07
Arsenic	ND	ND	ND	ND	ND
Barium	ND	ND	ND	ND	ND
Benzo(a)pyrene	0.47	2.20E-03	1.10E-03	2.00E-04	3.50E-03
Cadmium	ND	ND	ND	ND	ND
Chromium (III)	ND	ND	ND	ND	ND
Chromium (VI)	ND	ND	ND	ND	ND
Cobalt	ND	ND	ND	ND	ND
Copper	ND	ND	ND	ND	ND
Fluoranthene	0.88	3.00E-05	1.60E-05	ND	4.60E-05
Fluorene	0.01	4.30E-07	2.20E-07	ND	6.50E-07
Mercury	ND	ND	ND	ND	ND
Molybdenum	ND	ND	ND	ND	ND
Naphthalene	0.01	8.60E-07	4.40E-07	1.10E-04	1.10E-04
Nickel	ND	ND	ND	ND	ND
Phenol	0.01	2.30E-08	9.10E-09	2.10E-11	3.20E-08
Pyrene	0.75	3.40E-05	1.80E-05	ND	5.20E-05
Tin	ND	ND	ND	ND	ND
Zinc	ND	ND	ND	ND	ND
Totals		2.30E-03	1.10E-03	3.10E-04	3.70E-03

Table S24. Sector no. 31 – cancer risk – aggregate residents

Substance	Content (mg/kg)	CRo	CRd	CRinh	CR
Arsenic	ND	ND	ND	ND	ND
Benzo(a)anthracene	0.33	2.20E-07	8.50E-08	8.20E-09	3.10E-07
Benzo(a)pyrene	0.47	3.20E-06	1.20E-06	2.60E-10	4.40E-06
Benzo(b)fluoranthene	0.64	4.30E-07	1.60E-07	3.40E-11	5.90E-07
Benzo(k)fluoranthene	0.24	1.60E-08	6.30E-09	1.30E-12	2.30E-08
Cadmium	ND	ND	ND	ND	ND
Chromium (VI)	ND	ND	ND	ND	ND
Chrysene	0.30	2.00E-09	7.70E-10	1.60E-13	2.80E-09
Cobalt	ND	ND	ND	ND	ND
Dibenzo(a,h)anthracene	0.01	8.40E-08	3.20E-08	6.80E-12	1.20E-07
Indeno(1,2,3-cd)pyrene	0.39	2.60E-07	1.00E-07	2.10E-11	3.60E-07
Naphthalene	0.01	ND	ND	5.60E-09	5.60E-09
Nickel	ND	ND	ND	ND	ND
Totals		4.20E-06	1.60E-06	1.40E-08	5.80E-06

Table S25. Sector no. 32 – non-cancer risk – children

Substance	Content (mg/kg)	HQo	HQd	HQinh	HI
Acenaphtene	0.74	1.60E-04	5.70E-05	ND	2.10E-04
Anthracene	1.90	7.90E-05	2.90E-05	ND	1.10E-04
Arsenic	ND	ND	ND	ND	ND
Barium	ND	ND	ND	ND	ND
Benzo(a)pyrene	5.10	2.20E-01	7.90E-02	2.60E-03	3.00E-01
Cadmium	ND	ND	ND	ND	ND
Chromium (III)	ND	ND	ND	ND	ND
Chromium (VI)	ND	ND	ND	ND	ND
Cobalt	ND	ND	ND	ND	ND
Copper	ND	ND	ND	ND	ND
Fluoranthene	14.00	4.50E-03	1.60E-03	ND	6.10E-03
Fluorene	0.97	3.10E-04	1.10E-04	ND	4.20E-04
Mercury	ND	ND	ND	ND	ND
Molybdenum	ND	ND	ND	ND	ND
Naphthalene	0.49	3.10E-04	1.10E-04	1.00E-02	1.10E-02
Nickel	ND	ND	ND	ND	ND
Phenol	0.01	2.10E-07	6.00E-08	2.50E-11	2.70E-07
Pyrene	10.00	4.40E-03	1.60E-03	ND	6.00E-03
Tin	ND	ND	ND	ND	ND
Zinc	ND	ND	ND	ND	ND
Totals		2.30E-01	8.30E-02	1.30E-02	3.20E-01

Table S26. Sector no. 32 – non-cancer risk – adults

Substance	Content (mg/kg)	HQo	HQd	HQinh	HI
Acenaphtene	0.74	1.70E-05	8.80E-06	ND	2.60E-05
Anthracene	1.90	8.50E-06	4.40E-06	ND	1.30E-05
Arsenic	ND	ND	ND	ND	ND
Barium	ND	ND	ND	ND	ND
Benzo(a)pyrene	5.10	2.30E-02	1.20E-02	2.60E-03	3.80E-02
Cadmium	ND	ND	ND	ND	ND
Chromium (III)	ND	ND	ND	ND	ND
Chromium (VI)	ND	ND	ND	ND	ND
Cobalt	ND	ND	ND	ND	ND
Copper	ND	ND	ND	ND	ND
Fluoranthene	14.00	4.80E-04	2.50E-04	ND	7.30E-04
Fluorene	0.97	3.30E-05	1.70E-05	ND	5.10E-05
Mercury	ND	ND	ND	ND	ND
Molybdenum	ND	ND	ND	ND	ND
Naphthalene	0.49	3.40E-05	1.70E-05	5.10E-03	5.20E-03
Nickel	ND	ND	ND	ND	ND
Phenol	0.01	2.30E-08	9.10E-09	2.50E-11	3.20E-08
Pyrene	10.00	4.70E-04	2.40E-04	ND	7.10E-04
Tin	ND	ND	ND	ND	ND
Zinc	ND	ND	ND	ND	ND
Totals		2.40E-02	1.30E-02	7.70E-03	4.50E-02

Table S27. Sector no. 32 – cancer risk – aggregate residents

Substance	Content (mg/kg)	CRo	CRd	CRinh	CR
Arsenic	ND	ND	ND	ND	ND
Benzo(a)anthracene	6.40	4.30E-06	1.60E-06	1.90E-07	6.10E-06
Benzo(a)pyrene	5.10	3.40E-05	1.30E-05	3.40E-09	4.70E-05
Benzo(b)fluoranthene	7.50	5.00E-06	1.90E-06	5.00E-10	7.00E-06
Benzo(k)fluoranthene	2.60	1.70E-07	6.70E-08	1.70E-11	2.40E-07
Cadmium	ND	ND	ND	ND	ND
Chromium (VI)	ND	ND	ND	ND	ND
Chrysene	6.70	4.50E-08	1.70E-08	4.40E-12	6.20E-08
Cobalt	ND	ND	ND	ND	ND
Dibenzo(a,h)anthracene	0.88	5.90E-06	2.30E-06	5.80E-10	8.10E-06
Indeno(1,2,3-cd)pyrene	3.70	2.50E-06	9.40E-07	2.40E-10	3.40E-06
Naphthalene	0.49	ND	ND	2.70E-07	2.70E-07
Nickel	ND	ND	ND	ND	ND
Totals		5.20E-05	2.00E-05	4.60E-07	7.20E-05

Table S28. Sector no. 33 – non-cancer risk – children

Substance	Content (mg/kg)	HQo	HQd	HQinh	HI
Acenaphtene	0.01	2.70E-06	9.70E-07	ND	3.60E-06
Anthracene	0.08	3.20E-06	1.20E-06	ND	4.40E-06
Arsenic	ND	ND	ND	ND	ND
Barium	ND	ND	ND	ND	ND
Benzo(a)pyrene	0.63	2.70E-02	9.80E-03	3.20E-04	3.70E-02
Cadmium	ND	ND	ND	ND	ND
Chromium (III)	ND	ND	ND	ND	ND
Chromium (VI)	ND	ND	ND	ND	ND
Cobalt	ND	ND	ND	ND	ND
Copper	ND	ND	ND	ND	ND
Fluoranthene	1.10	3.50E-04	1.30E-04	ND	4.70E-04
Fluorene	0.01	4.00E-06	1.50E-06	ND	5.40E-06
Mercury	ND	ND	ND	ND	ND
Molybdenum	ND	ND	ND	ND	ND
Naphthalene	0.01	8.00E-06	2.90E-06	2.60E-04	2.70E-04
Nickel	ND	ND	ND	ND	ND
Phenol	0.01	2.10E-07	6.00E-08	2.50E-11	2.70E-07
Pyrene	1.10	4.50E-04	1.60E-04	ND	6.10E-04
Tin	ND	ND	ND	ND	ND
Zinc	ND	ND	ND	ND	ND
Totals		2.80E-02	1.00E-02	5.80E-04	3.80E-02

Table S29. Sector no. 33 – non-cancer risk – adults

Substance	Content (mg/kg)	HQo	HQd	HQinh	HI
Acenaphtene	0.01	2.90E-07	1.50E-07	ND	4.30E-07
Anthracene	0.08	3.40E-07	1.80E-07	ND	5.20E-07
Arsenic	ND	ND	ND	ND	ND
Barium	ND	ND	ND	ND	ND
Benzo(a)pyrene	0.63	2.90E-03	1.50E-03	3.20E-04	4.70E-03
Cadmium	ND	ND	ND	ND	ND
Chromium (III)	ND	ND	ND	ND	ND
Chromium (VI)	ND	ND	ND	ND	ND
Cobalt	ND	ND	ND	ND	ND
Copper	ND	ND	ND	ND	ND
Fluoranthene	1.10	3.70E-05	1.90E-05	ND	5.60E-05
Fluorene	0.01	4.30E-07	2.20E-07	ND	6.50E-07
Mercury	ND	ND	ND	ND	ND
Molybdenum	ND	ND	ND	ND	ND
Naphthalene	0.01	8.60E-07	4.40E-07	1.30E-04	1.30E-04
Nickel	0.00	ND	ND	ND	ND
Phenol	0.01	2.30E-08	9.10E-09	2.50E-11	3.20E-08
Pyrene	1.10	4.80E-05	2.50E-05	ND	7.30E-05
Tin	ND	ND	ND	ND	ND
Zinc	ND	ND	ND	ND	ND
Totals		3.00E-03	1.50E-03	4.50E-04	5.00E-03

Table S30. Sector no. 33 – cancer risk – aggregate residents

Substance	Content (mg/kg)	CRo	CRd	CRinh	CR
Arsenic	ND	ND	ND	ND	ND
Benzo(a)anthracene	0.72	4.80E-07	1.80E-07	2.20E-08	6.90E-07
Benzo(a)pyrene	0.63	4.20E-06	1.60E-06	4.20E-10	5.90E-06
Benzo(b)fluoranthene	1.10	7.60E-07	2.90E-07	7.40E-11	1.00E-06
Benzo(k)fluoranthene	0.41	2.70E-08	1.00E-08	2.70E-12	3.80E-08
Cadmium	ND	ND	ND	ND	ND
Chromium (VI)	ND	ND	ND	ND	ND
Chrysene	0.76	5.10E-09	2.00E-09	5.00E-13	7.10E-09
Cobalt	ND	ND	ND	ND	ND
Dibenzo(a,h)anthracene	0.10	6.40E-07	2.40E-07	6.30E-11	8.80E-07
Indeno(1,2,3-cd)pyrene	0.54	3.60E-07	1.40E-07	3.50E-11	5.00E-07
Naphthalene	0.01	ND	ND	6.80E-09	6.80E-09
Nickel	ND	ND	ND	ND	ND
Totals		6.50E-06	2.50E-06	2.90E-08	9.00E-06

Table S31. Sector no. 34– non-cancer risk – children

Substance	Content (mg/kg)	HQo	HQd	HQinh	HI
Acenaphtene	0.05	1.10E-05	4.10E-06	ND	1.50E-05
Anthracene	0.29	1.20E-05	4.50E-06	ND	1.70E-05
Arsenic	ND	ND	ND	ND	ND
Barium	ND	ND	ND	ND	ND
Benzo(a)pyrene	2.30	9.90E-02	3.60E-02	1.10E-03	1.40E-01
Cadmium	ND	ND	ND	ND	ND
Chromium (III)	ND	ND	ND	ND	ND
Chromium (VI)	ND	ND	ND	ND	ND
Cobalt	ND	ND	ND	ND	ND
Copper	ND	ND	ND	ND	ND
Fluoranthene	4.00	1.30E-03	4.60E-04	ND	1.70E-03
Fluorene	0.06	2.00E-05	7.30E-06	ND	2.70E-05
Mercury	ND	ND	ND	ND	ND
Molybdenum	ND	ND	ND	ND	ND
Naphthalene	0.01	8.00E-06	2.90E-06	2.50E-04	2.60E-04
Nickel	ND	ND	ND	ND	ND
Phenol	0.01	2.10E-07	6.00E-08	2.40E-11	2.70E-07
Pyrene	3.40	1.40E-03	5.30E-04	ND	2.00E-03
Tin	ND	ND	ND	ND	ND
Zinc	ND	ND	ND	ND	ND
Totals		1.00E-01	3.70E-02	1.40E-03	1.40E-01

Table S32. Sector no. 34 – non-cancer risk – adults

Substance	Content (mg/kg)	HQo	HQd	HQinh	HI
Acenaphtene	0.05	1.20E-06	6.30E-07	ND	1.80E-06
Anthracene	0.29	1.30E-06	6.80E-07	ND	2.00E-06
Arsenic	ND	ND	ND	ND	ND
Barium	ND	ND	ND	ND	ND
Benzo(a)pyrene	2.30	1.10E-02	5.50E-03	1.10E-03	1.70E-02
Cadmium	ND	ND	ND	ND	ND
Chromium (III)	ND	ND	ND	ND	ND
Chromium (VI)	ND	ND	ND	ND	ND
Cobalt	ND	ND	ND	ND	ND
Copper	ND	ND	ND	ND	ND
Fluoranthene	4.00	1.40E-04	7.10E-05	ND	2.10E-04
Fluorene	0.06	2.20E-06	1.10E-06	ND	3.30E-06
Mercury	ND	ND	ND	ND	ND
Molybdenum	ND	ND	ND	ND	ND
Naphthalene	0.01	8.60E-07	4.40E-07	1.20E-04	1.30E-04
Nickel	ND	ND	ND	ND	ND
Phenol	0.01	2.30E-08	9.10E-09	2.40E-11	3.20E-08
Pyrene	3.40	1.50E-04	8.00E-05	ND	2.40E-04
Tin	ND	ND	ND	ND	ND
Zinc	ND	ND	ND	ND	ND
Totals		1.10E-02	5.70E-03	1.20E-03	1.80E-02

Table S33. Sector no. 34 – cancer risk – aggregate residents

Substance	Content (mg/kg)	CRo	CRd	CRinh	CR
Arsenic	ND	ND	ND	ND	ND
Benzo(a)anthracene	1.30	8.90E-07	3.40E-07	3.80E-08	1.30E-06
Benzo(a)pyrene	2.30	1.60E-05	6.00E-06	1.50E-09	2.20E-05
Benzo(b)fluoranthene	3.40	2.30E-06	8.70E-07	2.10E-10	3.10E-06
Benzo(k)fluoranthene	1.20	8.00E-08	3.10E-08	7.60E-12	1.10E-07
Cadmium	ND	ND	ND	ND	ND
Chromium (VI)	ND	ND	ND	ND	ND
Chrysene	2.20	1.50E-08	5.70E-09	1.40E-12	2.10E-08
Cobalt	ND	ND	ND	ND	ND
Dibenzo(a,h)anthracene	0.37	2.50E-06	9.50E-07	2.30E-10	3.40E-06
Indeno(1,2,3-cd)pyrene	1.90	1.30E-06	4.80E-07	1.20E-10	1.70E-06
Naphthalene	0.01	ND	ND	6.50E-09	6.50E-09
Nickel	ND	ND	ND	ND	ND
Totals		2.30E-05	8.70E-06	4.70E-08	3.20E-05

Table S34. Sector no. 35 – non-cancer risk – children

Substance	Content (mg/kg)	HQo	HQd	HQinh	HI
Acenaphtene	2.20	4.80E-04	1.70E-04	ND	6.50E-04
Anthracene	3.00	1.30E-04	4.60E-05	ND	1.70E-04
Arsenic	ND	ND	ND	ND	ND
Barium	ND	ND	ND	ND	ND
Benzo(a)pyrene	3.10	1.30E-01	4.80E-02	1.60E-03	1.80E-01
Cadmium	ND	ND	ND	ND	ND
Chromium (III)	ND	ND	ND	ND	ND
Chromium (VI)	ND	ND	ND	ND	ND
Cobalt	ND	ND	ND	ND	ND
Copper	ND	ND	ND	ND	ND
Fluoranthene	8.60	2.70E-03	1.00E-03	ND	3.70E-03
Fluorene	1.80	5.80E-04	2.10E-04	ND	7.90E-04
Mercury	ND	ND	ND	ND	ND
Molybdenum	ND	ND	ND	ND	ND
Naphthalene	0.33	2.10E-04	7.60E-05	6.80E-03	7.10E-03
Nickel	ND	ND	ND	ND	ND
Phenol	0.01	2.10E-07	6.00E-08	2.50E-11	2.70E-07
Pyrene	7.20	3.10E-03	1.10E-03	ND	4.20E-03
Tin	ND	ND	ND	ND	ND
Zinc	ND	ND	ND	ND	ND
Totals		1.40E-01	5.10E-02	8.40E-03	2.00E-01

Table S35. Sector no. 35 – non-cancer risk – adults

Substance	Content (mg/kg)	HQo	HQd	HQinh	HI
Acenaphtene	2.20	5.10E-05	2.60E-05	ND	7.70E-05
Anthracene	3.00	1.30E-05	7.00E-06	ND	2.00E-05
Arsenic	ND	ND	ND	ND	ND
Barium	ND	ND	ND	ND	ND
Benzo(a)pyrene	3.10	1.40E-02	7.40E-03	1.60E-03	2.30E-02
Cadmium	ND	ND	ND	ND	ND
Chromium (III)	ND	ND	ND	ND	ND
Chromium (VI)	ND	ND	ND	ND	ND
Cobalt	ND	ND	ND	ND	ND
Copper	ND	ND	ND	ND	ND
Fluoranthene	8.60	2.90E-04	1.50E-04	ND	4.50E-04
Fluorene	1.80	6.20E-05	3.20E-05	ND	9.50E-05
Mercury	ND	ND	ND	ND	ND
Molybdenum	ND	ND	ND	ND	ND
Naphthalene	0.33	2.20E-05	1.20E-05	3.40E-03	3.50E-03
Nickel	ND	ND	ND	ND	ND
Phenol	0.01	2.30E-08	9.10E-09	2.50E-11	3.20E-08
Pyrene	7.20	3.30E-04	1.70E-04	ND	5.00E-04
Tin	ND	ND	ND	ND	ND
Zinc	ND	ND	ND	ND	ND
Totals		1.50E-02	7.80E-03	5.00E-03	2.80E-02

Table S36. Sector no. 35 – cancer risk – aggregate residents

Substance	Content (mg/kg)	CRo	CRd	CRinh	CR
Arsenic	ND	ND	ND	ND	ND
Benzo(a)anthracene	4.30	2.80E-06	1.10E-06	1.30E-07	4.10E-06
Benzo(a)pyrene	3.10	2.10E-05	8.00E-06	2.10E-09	2.90E-05
Benzo(b)fluoranthene	3.80	2.60E-06	9.90E-07	2.50E-10	3.60E-06
Benzo(k)fluoranthene	1.40	9.40E-08	3.60E-08	9.30E-12	1.30E-07
Cadmium	ND	ND	ND	ND	ND
Chromium (VI)	ND	ND	ND	ND	ND
Chrysene	3.50	2.40E-08	9.00E-09	2.30E-12	3.30E-08
Cobalt	ND	ND	ND	ND	ND
Dibenzo(a,h)anthracene	0.41	2.70E-06	1.00E-06	2.70E-10	3.80E-06
Indeno(1,2,3-cd)pyrene	1.50	9.90E-07	3.80E-07	9.70E-11	1.40E-06
Naphthalene	0.33	ND	ND	1.80E-07	1.80E-07
Nickel	ND	ND	ND	ND	ND
Totals		3.00E-05	1.20E-05	3.10E-07	4.20E-05

Table S37. Sector no. 41 – non-cancer risk – children

Substance	Content (mg/kg)	HQo	HQd	HQinh	HI
Acenaphtene	0.01	2.70E-06	9.70E-07	ND	3.60E-06
Anthracene	0.01	5.30E-07	1.90E-07	ND	7.30E-07
Arsenic	ND	ND	ND	ND	ND
Barium	ND	ND	ND	ND	ND
Benzo(a)pyrene	0.01	5.30E-04	1.90E-04	6.00E-06	7.30E-04
Cadmium	ND	ND	ND	ND	ND
Chromium (III)	ND	ND	ND	ND	ND
Chromium (VI)	ND	ND	ND	ND	ND
Cobalt	ND	ND	ND	ND	ND
Copper	ND	ND	ND	ND	ND
Fluoranthene	0.01	4.00E-06	1.50E-06	ND	5.40E-06
Fluorene	0.01	4.00E-06	1.50E-06	ND	5.40E-06
Mercury	ND	ND	ND	ND	ND
Molybdenum	ND	ND	ND	ND	ND
Naphthalene	0.01	8.00E-06	2.90E-06	2.50E-04	2.60E-04
Nickel	ND	ND	ND	ND	ND
Phenol	0.01	2.10E-07	6.00E-08	2.40E-11	2.70E-07
Pyrene	0.01	5.30E-06	1.90E-06	ND	7.30E-06
Tin	ND	ND	ND	ND	ND
Zinc	ND	ND	ND	ND	ND
Totals		5.50E-04	2.00E-04	2.60E-04	1.00E-03

Table S38. Sector no. 41 – non-cancer risk – adults

Substance	Content (mg/kg)	HQo	HQd	HQinh	HI
Acenaphtene	0.01	2.90E-07	1.50E-07	ND	4.30E-07
Anthracene	0.01	5.70E-08	3.00E-08	ND	8.70E-08
Arsenic	ND	ND	ND	ND	ND
Barium	ND	ND	ND	ND	ND
Benzo(a)pyrene	0.01	5.70E-05	3.00E-05	6.00E-06	9.30E-05
Cadmium	ND	ND	ND	ND	ND
Chromium (III)	ND	ND	ND	ND	ND
Chromium (VI)	ND	ND	ND	ND	ND
Cobalt	ND	ND	ND	ND	ND
Copper	ND	ND	ND	ND	ND
Fluoranthene	0.01	4.30E-07	2.20E-07	ND	6.50E-07
Fluorene	0.01	4.30E-07	2.20E-07	ND	6.50E-07
Mercury	ND	ND	ND	ND	ND
Molybdenum	ND	ND	ND	ND	ND
Naphthalene	0.01	8.60E-07	4.40E-07	1.20E-04	1.20E-04
Nickel	ND	ND	ND	ND	ND
Phenol	0.01	2.30E-08	9.10E-09	2.40E-11	3.20E-08
Pyrene	0.01	5.70E-07	3.00E-07	ND	8.70E-07
Tin	ND	ND	ND	ND	ND
Zinc	ND	ND	ND	ND	ND
Totals		6.00E-05	3.10E-05	1.30E-04	2.20E-04

Table S39. Sector no. 41 – cancer risk – aggregate residents

Substance	Content (mg/kg)	CRo	CRd	CRinh	CR
Arsenic	ND	ND	ND	ND	ND
Benzo(a)anthracene	0.01	8.40E-09	3.20E-09	3.60E-10	1.20E-08
Benzo(a)pyrene	0.01	8.40E-08	3.20E-08	7.80E-12	1.20E-07
Benzo(b)fluoranthene	0.01	8.40E-09	3.20E-09	7.80E-13	1.20E-08
Benzo(k)fluoranthene	0.01	8.40E-10	3.20E-10	7.80E-14	1.20E-09
Cadmium	ND	ND	ND	ND	ND
Chromium (VI)	ND	ND	ND	ND	ND
Chrysene	0.01	8.40E-11	3.20E-11	8.00E-15	1.20E-10
Cobalt	ND	ND	ND	ND	ND
Dibenzo(a,h)anthracene	0.01	8.40E-08	3.20E-08	7.80E-12	1.20E-07
Indeno(1,2,3-cd)pyrene	0.01	8.40E-09	3.20E-09	7.80E-13	1.20E-08
Naphthalene	0.01	ND	ND	6.50E-09	6.50E-09
Nickel	ND	ND	ND	ND	ND
Totals		1.90E-07	7.40E-08	6.90E-09	2.80E-07

Table S40. Sector no. 42– non-cancer risk – children

Substance	Content (mg/kg)	HQo	HQd	HQinh	HI
Acenaphtene	0.01	2.70E-06	9.70E-07	ND	3.60E-06
Anthracene	0.01	5.30E-07	1.90E-07	ND	7.30E-07
Arsenic	ND	ND	ND	ND	ND
Barium	ND	ND	ND	ND	ND
Benzo(a)pyrene	0.01	5.30E-04	1.90E-04	6.20E-06	7.30E-04
Cadmium	ND	ND	ND	ND	ND
Chromium (III)	ND	ND	ND	ND	ND
Chromium (VI)	ND	ND	ND	ND	ND
Cobalt	ND	ND	ND	ND	ND
Copper	ND	ND	ND	ND	ND
Fluoranthene	0.01	4.00E-06	1.50E-06	ND	5.40E-06
Fluorene	0.01	4.00E-06	1.50E-06	ND	5.40E-06
Mercury	ND	ND	ND	ND	ND
Molybdenum	ND	ND	ND	ND	ND
Naphthalene	0.01	8.00E-06	2.90E-06	2.50E-04	2.60E-04
Nickel	ND	ND	ND	ND	ND
Phenol	0.01	2.10E-07	6.00E-08	2.50E-11	2.70E-07
Pyrene	0.01	5.30E-06	1.90E-06	ND	7.30E-06
Tin	ND	ND	ND	ND	ND
Zinc	ND	ND	ND	ND	ND
Totals		5.50E-04	2.00E-04	2.60E-04	1.00E-03

Table S41. Sector no. 42 – non-cancer risk – adults

Substance	Content (mg/kg)	HQo	HQd	HQinh	HI
Acenaphtene	0.01	2.90E-07	1.50E-07	ND	4.30E-07
Anthracene	0.01	5.70E-08	3.00E-08	ND	8.70E-08
Arsenic	ND	ND	ND	ND	ND
Barium	ND	ND	ND	ND	ND
Benzo(a)pyrene	0.01	5.70E-05	3.00E-05	6.20E-06	9.30E-05
Cadmium	ND	ND	ND	ND	ND
Chromium (III)	ND	ND	ND	ND	ND
Chromium (VI)	ND	ND	ND	ND	ND
Cobalt	ND	ND	ND	ND	ND
Copper	ND	ND	ND	ND	ND
Fluoranthene	0.01	4.30E-07	2.20E-07	ND	6.50E-07
Fluorene	0.01	4.30E-07	2.20E-07	ND	6.50E-07
Mercury	ND	ND	ND	ND	ND
Molybdenum	ND	ND	ND	ND	ND
Naphthalene	0.01	8.60E-07	4.40E-07	1.30E-04	1.30E-04
Nickel	ND	ND	ND	ND	ND
Phenol	0.01	2.30E-08	9.10E-09	2.50E-11	3.20E-08
Pyrene	0.01	5.70E-07	3.00E-07	ND	8.70E-07
Tin	ND	ND	ND	ND	ND
Zinc	ND	ND	ND	ND	ND
Totals		6.00E-05	3.10E-05	1.40E-04	2.30E-04

Table S42. Sector no. 42 – cancer risk – aggregate residents

Substance	Content (mg/kg)	CRo	CRd	CRinh	CR
Arsenic	ND	ND	ND	ND	ND
Benzo(a)anthracene	0.01	8.40E-09	3.20E-09	3.70E-10	1.20E-08
Benzo(a)pyrene	0.01	8.40E-08	3.20E-08	8.00E-12	1.20E-07
Benzo(b)fluoranthene	0.01	8.40E-09	3.20E-09	8.00E-13	1.20E-08
Benzo(k)fluoranthene	0.01	8.40E-10	3.20E-10	8.00E-14	1.20E-09
Cadmium	ND	ND	ND	ND	ND
Chromium (VI)	ND	ND	ND	ND	ND
Chrysene	0.01	8.40E-11	3.20E-11	8.00E-15	1.20E-10
Cobalt	ND	ND	ND	ND	ND
Dibenzo(a,h)anthracene	0.01	8.40E-08	3.20E-08	8.00E-12	1.20E-07
Indeno(1,2,3-cd)pyrene	0.01	8.40E-09	3.20E-09	8.00E-13	1.20E-08
Naphthalene	0.01	ND	ND	6.70E-09	6.70E-09
Nickel	ND	ND	ND	ND	ND
Totals		1.90E-07	7.40E-08	7.10E-09	2.80E-07

Table S43. Sector no. 43 – non-cancer risk – children

Substance	Content (mg/kg)	HQo	HQd	HQinh	HI
Acenaphtene	0.03	5.30E-06	1.90E-06	ND	7.30E-06
Anthracene	0.05	2.20E-06	7.90E-07	ND	3.00E-06
Arsenic	ND	ND	ND	ND	ND
Barium	ND	ND	ND	ND	ND
Benzo(a)pyrene	0.07	2.80E-03	1.00E-03	3.30E-05	3.80E-03
Cadmium	ND	ND	ND	ND	ND
Chromium (III)	ND	ND	ND	ND	ND
Chromium (VI)	ND	ND	ND	ND	ND
Cobalt	ND	ND	ND	ND	ND
Copper	ND	ND	ND	ND	ND
Fluoranthene	0.16	5.20E-05	1.90E-05	ND	7.20E-05
Fluorene	0.03	9.90E-06	3.60E-06	ND	1.40E-05
Mercury	ND	ND	ND	ND	ND
Molybdenum	ND	ND	ND	ND	ND
Naphthalene	0.01	8.00E-06	2.90E-06	2.60E-04	2.70E-04
Nickel	ND	ND	ND	ND	ND
Phenol	0.01	2.10E-07	6.00E-08	2.50E-11	2.70E-07
Pyrene	0.13	5.50E-05	2.00E-05	ND	7.40E-05
Tin	ND	ND	ND	ND	ND
Zinc	ND	ND	ND	ND	ND
Totals		2.90E-03	1.00E-03	2.90E-04	4.20E-03

Table S44. Sector no. 43 – non-cancer risk – adults

Substance	Content (mg/kg)	HQo	HQd	HQinh	HI
Acenaphtene	0.03	5.70E-07	3.00E-07	ND	8.70E-07
Anthracene	0.05	2.30E-07	1.20E-07	ND	3.50E-07
Arsenic	ND	ND	ND	ND	ND
Barium	ND	ND	ND	ND	ND
Benzo(a)pyrene	0.07	3.00E-04	1.50E-04	3.30E-05	4.80E-04
Cadmium	ND	ND	ND	ND	ND
Chromium (III)	ND	ND	ND	ND	ND
Chromium (VI)	ND	ND	ND	ND	ND
Cobalt	ND	ND	ND	ND	ND
Copper	ND	ND	ND	ND	ND
Fluoranthene	0.16	5.60E-06	2.90E-06	ND	8.50E-06
Fluorene	0.03	1.10E-06	5.50E-07	ND	1.60E-06
Mercury	ND	ND	ND	ND	ND
Molybdenum	ND	ND	ND	ND	ND
Naphthalene	0.01	8.60E-07	4.40E-07	1.30E-04	1.30E-04
Nickel	ND	ND	ND	ND	ND
Phenol	0.01	2.30E-08	9.10E-09	2.50E-11	3.20E-08
Pyrene	0.13	5.80E-06	3.00E-06	ND	8.90E-06
Tin	ND	ND	ND	ND	ND
Zinc	ND	ND	ND	ND	ND
Totals		3.10E-04	1.60E-04	1.60E-04	6.30E-04

Table S45. Sector no. 43 – cancer risk – aggregate residents

Substance	Content (mg/kg)	CRo	CRd	CRinh	CR
Arsenic	ND	ND	ND	ND	ND
Benzo(a)anthracene	0.07	4.90E-08	1.90E-08	2.20E-09	7.00E-08
Benzo(a)pyrene	0.07	4.40E-07	1.70E-07	4.30E-11	6.00E-07
Benzo(b)fluoranthene	0.08	5.50E-08	2.10E-08	5.40E-12	7.60E-08
Benzo(k)fluoranthene	0.03	1.90E-09	7.20E-10	1.80E-13	2.60E-09
Cadmium	ND	ND	ND	ND	ND
Chromium (VI)	ND	ND	ND	ND	ND
Chrysene	0.07	4.80E-10	1.90E-10	4.70E-14	6.70E-10
Cobalt	ND	ND	ND	ND	ND
Dibenzo(a,h)anthracene	0.01	8.40E-08	3.20E-08	8.20E-12	1.20E-07
Indeno(1,2,3-cd)pyrene	0.04	2.90E-08	1.10E-08	2.80E-12	4.00E-08
Naphthalene	0.01	ND	ND	6.80E-09	6.80E-09
Nickel	ND	ND	ND	ND	ND
Totals		6.60E-07	2.50E-07	9.10E-09	9.20E-07

Table S46. Sector no. 44 – non-cancer risk – children

Substance	Content (mg/kg)	HQo	HQd	HQinh	HI
Acenaphtene	0.03	7.20E-06	2.60E-06	ND	9.90E-06
Anthracene	0.09	3.80E-06	1.40E-06	ND	5.20E-06
Arsenic	ND	ND	ND	ND	ND
Barium	ND	ND	ND	ND	ND
Benzo(a)pyrene	0.58	2.50E-02	8.90E-03	3.00E-04	3.40E-02
Cadmium	ND	ND	ND	ND	ND
Chromium (III)	ND	ND	ND	ND	ND
Chromium (VI)	ND	ND	ND	ND	ND
Cobalt	ND	ND	ND	ND	ND
Copper	ND	ND	ND	ND	ND
Fluoranthene	0.94	3.00E-04	1.10E-04	ND	4.10E-04
Fluorene	0.03	1.00E-05	3.70E-06	ND	1.40E-05
Mercury	ND	ND	ND	ND	ND
Molybdenum	ND	ND	ND	ND	ND
Naphthalene	0.01	8.00E-06	2.90E-06	2.60E-04	2.70E-04
Nickel	ND	ND	ND	ND	ND
Phenol	0.01	2.10E-07	6.00E-08	2.60E-11	2.70E-07
Pyrene	0.81	3.40E-04	1.20E-04	ND	4.70E-04
Tin	ND	ND	ND	ND	ND
Zinc	ND	ND	ND	ND	ND
Totals		2.60E-02	9.10E-03	5.60E-04	3.50E-02

Table S47. Sector no. 44 – non-cancer risk – adults

Substance	Content (mg/kg)	HQo	HQd	HQinh	HI
Acenaphtene	0.03	7.80E-07	4.00E-07	ND	1.20E-06
Anthracene	0.09	4.10E-07	2.10E-07	ND	6.20E-07
Arsenic	ND	ND	ND	ND	ND
Barium	ND	ND	ND	ND	ND
Benzo(a)pyrene	0.58	2.60E-03	1.40E-03	3.00E-04	4.30E-03
Cadmium	ND	ND	ND	ND	ND
Chromium (III)	ND	ND	ND	ND	ND
Chromium (VI)	ND	ND	ND	ND	ND
Cobalt	ND	ND	ND	ND	ND
Copper	ND	ND	ND	ND	ND
Fluoranthene	0.94	3.20E-05	1.70E-05	ND	4.90E-05
Fluorene	0.03	1.10E-06	5.70E-07	ND	1.70E-06
Mercury	0.00	ND	ND	ND	ND
Molybdenum	0.00	ND	ND	ND	ND
Naphthalene	0.01	8.60E-07	4.40E-07	1.30E-04	1.30E-04
Nickel	ND	ND	ND	ND	ND
Phenol	0.01	2.30E-08	9.10E-09	2.60E-11	3.20E-08
Pyrene	0.81	3.70E-05	1.90E-05	ND	5.60E-05
Tin	ND	ND	ND	ND	ND
Zinc	ND	ND	ND	ND	ND
Totals		2.70E-03	1.40E-03	4.30E-04	4.50E-03

Table S48. Sector no. 44 – cancer risk – aggregate residents

Substance	Content (mg/kg)	CRo	CRd	CRinh	CR
Arsenic	ND	ND	ND	ND	ND
Benzo(a)anthracene	0.49	3.30E-07	1.30E-07	1.50E-08	4.70E-07
Benzo(a)pyrene	0.58	3.90E-06	1.50E-06	3.80E-10	5.30E-06
Benzo(b)fluoranthene	0.82	5.50E-07	2.10E-07	5.40E-11	7.60E-07
Benzo(k)fluoranthene	0.29	1.90E-08	7.40E-09	1.90E-12	2.70E-08
Cadmium	ND	ND	ND	ND	ND
Chromium (VI)	ND	ND	ND	ND	ND
Chrysene	0.54	3.60E-09	1.40E-09	3.60E-13	5.00E-09
Cobalt	ND	ND	ND	ND	ND
Dibenzo(a,h)anthracene	0.09	6.00E-07	2.30E-07	6.00E-11	8.30E-07
Indeno(1,2,3-cd)pyrene	0.50	3.40E-07	1.30E-07	3.30E-11	4.60E-07
Naphthalene	0.01	ND	ND	6.90E-09	6.90E-09
Nickel	ND	ND	ND	ND	ND
Totals		5.70E-06	2.20E-06	2.20E-08	7.90E-06

Table S49. Sector no. 45 – non-cancer risk – children

Substance	Content (mg/kg)	HQo	HQd	HQinh	HI
Acenaphtene	0.01	2.70E-06	9.70E-07	ND	3.60E-06
Anthracene	0.04	1.70E-06	6.40E-07	ND	2.40E-06
Arsenic	ND	ND	ND	ND	ND
Barium	ND	ND	ND	ND	ND
Benzo(a)pyrene	0.32	1.30E-02	4.90E-03	1.60E-04	1.90E-02
Cadmium	ND	ND	ND	ND	ND
Chromium (III)	ND	ND	ND	ND	ND
Chromium (VI)	ND	ND	ND	ND	ND
Cobalt	ND	ND	ND	ND	ND
Copper	ND	ND	ND	ND	ND
Fluoranthene	0.56	1.80E-04	6.50E-05	ND	2.40E-04
Fluorene	0.01	4.00E-06	1.50E-06	ND	5.40E-06
Mercury	ND	ND	ND	ND	ND
Molybdenum	ND	ND	ND	ND	ND
Naphthalene	0.01	8.00E-06	2.90E-06	2.60E-04	2.70E-04
Nickel	ND	ND	ND	ND	ND
Phenol	0.01	2.10E-07	6.00E-08	2.50E-11	2.70E-07
Pyrene	0.47	2.00E-04	7.20E-05	ND	2.70E-04
Tin	ND	ND	ND	ND	ND
Zinc	ND	ND	ND	ND	ND
Totals		1.30E-02	5.00E-03	4.20E-04	2.00E-02

Table S50. Sector no. 45 – non-cancer risk – adults

Substance	Content (mg/kg)	HQo	HQd	HQinh	HI
Acenaphtene	0.01	2.90E-07	1.50E-07	ND	4.30E-07
Anthracene	0.04	1.90E-07	9.70E-08	ND	2.80E-07
Arsenic	ND	ND	ND	ND	ND
Barium	ND	ND	ND	ND	ND
Benzo(a)pyrene	0.32	1.40E-03	7.50E-04	1.60E-04	2.40E-03
Cadmium	ND	ND	ND	ND	ND
Chromium (III)	ND	ND	ND	ND	ND
Chromium (VI)	ND	ND	ND	ND	ND
Cobalt	ND	ND	ND	ND	ND
Copper	ND	ND	ND	ND	ND
Fluoranthene	0.56	1.90E-05	9.90E-06	ND	2.90E-05
Fluorene	0.01	4.30E-07	2.20E-07	ND	6.50E-07
Mercury	ND	ND	ND	ND	ND
Molybdenum	ND	ND	ND	ND	ND
Naphthalene	ND	8.60E-07	4.40E-07	1.30E-04	1.30E-04
Nickel	ND	ND	ND	ND	ND
Phenol	0.01	2.30E-08	9.10E-09	2.50E-11	3.20E-08
Pyrene	0.47	2.10E-05	1.10E-05	ND	3.20E-05
Tin	ND	ND	ND	ND	ND
Zinc	ND	ND	ND	ND	ND
Totals		1.40E-03	7.70E-04	2.90E-04	2.60E-03

Table S51. Sector no. 45 – cancer risk – aggregate residents

Substance	Content (mg/kg)	CRo	CRd	CRinh	CR
Arsenic	ND	ND	ND	ND	ND
Benzo(a)anthracene	0.28	1.90E-07	7.20E-08	8.40E-09	2.70E-07
Benzo(a)pyrene	0.32	2.10E-06	8.10E-07	2.10E-10	2.90E-06
Benzo(b)fluoranthene	0.45	3.00E-07	1.10E-07	2.90E-11	4.10E-07
Benzo(k)fluoranthene	0.17	1.10E-08	4.20E-09	1.10E-12	1.50E-08
Cadmium	ND	ND	ND	ND	ND
Chromium (VI)	ND	ND	ND	ND	ND
Chrysene	0.31	2.10E-09	8.00E-10	2.00E-13	2.90E-09
Cobalt	ND	ND	ND	ND	ND
Dibenzo(a,h)anthracene	0.05	3.60E-07	1.40E-07	3.50E-11	5.00E-07
Indeno(1,2,3-cd)pyrene	0.26	1.70E-07	6.60E-08	1.70E-11	2.40E-07
Naphthalene	0.01	ND	ND	6.80E-09	6.80E-09
Nickel	ND	ND	ND	ND	ND
Totals		3.10E-06	1.20E-06	1.50E-08	4.30E-06

Table S52. Sector no. 46 – non-cancer risk – children

Substance	Content (mg/kg)	HQo	HQd	HQinh	HI
Acenaphtene	0.01	2.70E-06	9.70E-07	ND	3.60E-06
Anthracene	0.05	2.30E-06	8.40E-07	ND	3.10E-06
Arsenic	ND	ND	ND	ND	ND
Barium	ND	ND	ND	ND	ND
Benzo(a)pyrene	0.21	8.90E-03	3.20E-03	1.00E-04	1.20E-02
Cadmium	ND	ND	ND	ND	ND
Chromium (III)	ND	ND	ND	ND	ND
Chromium (VI)	ND	ND	ND	ND	ND
Cobalt	ND	ND	ND	ND	ND
Copper	ND	ND	ND	ND	ND
Fluoranthene	0.46	1.50E-04	5.40E-05	ND	2.00E-04
Fluorene	0.01	4.00E-06	1.50E-06	ND	5.40E-06
Mercury	ND	ND	ND	ND	ND
Molybdenum	ND	ND	ND	ND	ND
Naphthalene	0.01	8.00E-06	2.90E-06	2.50E-04	2.60E-04
Nickel	ND	ND	ND	ND	ND
Phenol	0.01	2.10E-07	6.00E-08	2.40E-11	2.70E-07
Pyrene	0.37	1.60E-04	5.70E-05	ND	2.10E-04
Tin	ND	ND	ND	ND	ND
Zinc	ND	ND	ND	ND	ND
Totals		9.20E-03	3.30E-03	3.50E-04	1.30E-02

Table S53. Sector no. 46 – non-cancer risk – adults

Substance	Content (mg/kg)	HQo	HQd	HQinh	HI
Acenaphtene	0.01	2.90E-07	1.50E-07	ND	4.30E-07
Anthracene	0.05	2.50E-07	1.30E-07	ND	3.70E-07
Arsenic	ND	ND	ND	ND	ND
Barium	ND	ND	ND	ND	ND
Benzo(a)pyrene	0.21	9.50E-04	4.90E-04	1.00E-04	1.50E-03
Cadmium	ND	ND	ND	ND	ND
Chromium (III)	ND	ND	ND	ND	ND
Chromium (VI)	ND	ND	ND	ND	ND
Cobalt	ND	ND	ND	ND	ND
Copper	ND	ND	ND	ND	ND
Fluoranthene	0.46	1.60E-05	8.20E-06	ND	2.40E-05
Fluorene	0.01	4.30E-07	2.20E-07	ND	6.50E-07
Mercury	ND	ND	ND	ND	ND
Molybdenum	ND	ND	ND	ND	ND
Naphthalene	0.01	8.60E-07	4.40E-07	1.30E-04	1.30E-04
Nickel	ND	ND	ND	ND	ND
Phenol	0.01	2.30E-08	9.10E-09	2.40E-11	3.20E-08
Pyrene	0.37	1.70E-05	8.70E-06	ND	2.50E-05
Tin	ND	ND	ND	ND	ND
Zinc	ND	ND	ND	ND	ND
Totals		9.80E-04	5.10E-04	2.30E-04	1.70E-03

Table S54. Sector no. 46 – cancer risk – aggregate residents

Substance	Content (mg/kg)	CRo	CRd	CRinh	CR
Arsenic	ND	ND	ND	ND	ND
Benzo(a)anthracene	0.21	1.40E-07	5.40E-08	6.10E-09	2.00E-07
Benzo(a)pyrene	0.21	1.40E-06	5.40E-07	1.30E-10	1.90E-06
Benzo(b)fluoranthene	0.29	2.00E-07	7.50E-08	1.90E-11	2.70E-07
Benzo(k)fluoranthene	0.11	7.00E-09	2.70E-09	6.70E-13	9.70E-09
Cadmium	ND	ND	ND	ND	ND
Chromium (VI)	ND	ND	ND	ND	ND
Chrysene	0.22	1.40E-09	5.50E-10	1.40E-13	2.00E-09
Cobalt	ND	ND	ND	ND	ND
Dibenzo(a,h)anthracene	0.03	1.70E-07	6.70E-08	1.70E-11	2.40E-07
Indeno(1,2,3-cd)pyrene	0.16	1.10E-07	4.20E-08	1.00E-11	1.50E-07
Naphthalene	0.01	ND	ND	6.60E-09	6.60E-09
Nickel	ND	ND	ND	ND	ND
Totals		2.00E-06	7.80E-07	1.30E-08	2.80E-06

Table S55. Sector no. 47 – non-cancer risk – children

Substance	Content (mg/kg)	HQo	HQd	HQinh	HI
Acenaphtene	0.01	2.70E-06	9.70E-07	ND	3.60E-06
Anthracene	0.10	4.10E-06	1.50E-06	ND	5.60E-06
Arsenic	ND	ND	ND	ND	ND
Barium	ND	ND	ND	ND	ND
Benzo(a)pyrene	0.64	2.70E-02	9.90E-03	3.30E-04	3.70E-02
Cadmium	ND	ND	ND	ND	ND
Chromium (III)	ND	ND	ND	ND	ND
Chromium (VI)	ND	ND	ND	ND	ND
Cobalt	ND	ND	ND	ND	ND
Copper	ND	ND	ND	ND	ND
Fluoranthene	1.20	3.70E-04	1.40E-04	ND	5.10E-04
Fluorene	0.03	9.90E-06	3.60E-06	ND	1.40E-05
Mercury	ND	ND	ND	ND	ND
Molybdenum	ND	ND	ND	ND	ND
Naphthalene	0.01	8.00E-06	2.90E-06	2.60E-04	2.70E-04
Nickel	ND	ND	ND	ND	ND
Phenol	0.01	2.10E-07	6.00E-08	2.60E-11	2.70E-07
Pyrene	0.99	4.20E-04	1.50E-04	ND	5.80E-04
Tin	ND	ND	ND	ND	ND
Zinc	ND	ND	ND	ND	ND
Totals		2.80E-02	1.00E-02	5.90E-04	3.80E-02

Table S56. Sector no. 47 – non-cancer risk – adults

Substance	Content (mg/kg)	HQo	HQd	HQinh	HI
Acenaphtene	0.01	2.90E-07	1.50E-07	ND	4.30E-07
Anthracene	0.10	4.40E-07	2.30E-07	ND	6.70E-07
Arsenic	ND	ND	ND	ND	ND
Barium	ND	ND	ND	ND	ND
Benzo(a)pyrene	0.64	2.90E-03	1.50E-03	3.30E-04	4.80E-03
Cadmium	ND	ND	ND	ND	ND
Chromium (III)	ND	ND	ND	ND	ND
Chromium (VI)	ND	ND	ND	ND	ND
Cobalt	ND	ND	ND	ND	ND
Copper	ND	ND	ND	ND	ND
Fluoranthene	1.20	4.00E-05	2.10E-05	ND	6.10E-05
Fluorene	0.03	1.10E-06	5.50E-07	ND	1.60E-06
Mercury	ND	ND	ND	ND	ND
Molybdenum	ND	ND	ND	ND	ND
Naphthalene	0.01	8.60E-07	4.40E-07	1.30E-04	1.30E-04
Nickel	ND	ND	ND	ND	ND
Phenol	0.01	2.30E-08	9.10E-09	2.60E-11	3.20E-08
Pyrene	0.99	4.50E-05	2.30E-05	ND	6.90E-05
Tin	ND	ND	ND	ND	ND
Zinc	ND	ND	ND	ND	ND
Totals		3.00E-03	1.50E-03	4.60E-04	5.10E-03

Table S57. Sector no. 47 – cancer risk – aggregate residents

Substance	Content (mg/kg)	CRo	CRd	CRinh	CR
Arsenic	ND	ND	ND	ND	ND
Benzo(a)anthracene	0.56	3.80E-07	1.40E-07	1.70E-08	5.40E-07
Benzo(a)pyrene	0.64	4.30E-06	1.60E-06	4.20E-10	5.90E-06
Benzo(b)fluoranthene	0.95	6.30E-07	2.40E-07	6.30E-11	8.80E-07
Benzo(k)fluoranthene	0.32	2.20E-08	8.30E-09	2.10E-12	3.00E-08
Cadmium	ND	ND	ND	ND	ND
Chromium (VI)	ND	ND	ND	ND	ND
Chrysene	0.65	4.30E-09	1.70E-09	4.30E-13	6.00E-09
Cobalt	ND	ND	ND	ND	ND
Dibenzo(a,h)anthracene	0.11	7.10E-07	2.70E-07	7.00E-11	9.80E-07
Indeno(1,2,3-cd)pyrene	0.53	3.60E-07	1.40E-07	3.50E-11	4.90E-07
Naphthalene	0.01	ND	ND	6.90E-09	6.90E-09
Nickel	ND	ND	ND	ND	ND
Totals		6.40E-06	2.40E-06	2.40E-08	8.80E-06

Table S58. Sector no. 51 – non-cancer risk – children

Substance	Content (mg/kg)	HQo	HQd	HQinh	HI
Acenaphtene	0.04	7.50E-06	2.70E-06	ND	1.00E-05
Anthracene	0.06	2.60E-06	9.60E-07	ND	3.60E-06
Arsenic	ND	ND	ND	ND	ND
Barium	ND	ND	ND	ND	ND
Benzo(a)pyrene	0.35	1.50E-02	5.40E-03	1.70E-04	2.00E-02
Cadmium	ND	ND	ND	ND	ND
Chromium (III)	ND	ND	ND	ND	ND
Chromium (VI)	ND	ND	ND	ND	ND
Cobalt	ND	ND	ND	ND	ND
Copper	ND	ND	ND	ND	ND
Fluoranthene	0.78	2.50E-04	9.00E-05	ND	3.40E-04
Fluorene	0.03	9.90E-06	3.60E-06	ND	1.40E-05
Mercury	ND	ND	ND	ND	ND
Molybdenum	ND	ND	ND	ND	ND
Naphthalene	0.01	8.00E-06	2.90E-06	2.60E-04	2.70E-04
Nickel	ND	ND	ND	ND	ND
Phenol	0.01	2.10E-07	6.00E-08	2.50E-11	2.70E-07
Pyrene	0.64	2.70E-04	9.90E-05	ND	3.70E-04
Tin	ND	ND	ND	ND	ND
Zinc	ND	ND	ND	ND	ND
Totals		1.60E-02	5.60E-03	4.30E-04	2.10E-02

Table S59. Sector no. 51 – non-cancer risk – adults

Substance	Content (mg/kg)	HQo	HQd	HQinh	HI
Acenaphtene	0.04	8.00E-07	4.10E-07	ND	1.20E-06
Anthracene	0.06	2.80E-07	1.50E-07	ND	4.30E-07
Arsenic	ND	ND	ND	ND	ND
Barium	ND	ND	ND	ND	ND
Benzo(a)pyrene	0.35	1.60E-03	8.20E-04	1.70E-04	2.60E-03
Cadmium	ND	ND	ND	ND	ND
Chromium (III)	ND	ND	ND	ND	ND
Chromium (VI)	ND	ND	ND	ND	ND
Cobalt	ND	ND	ND	ND	ND
Copper	ND	ND	ND	ND	ND
Fluoranthene	0.78	2.70E-05	1.40E-05	ND	4.00E-05
Fluorene	0.03	1.10E-06	5.50E-07	ND	1.60E-06
Mercury	ND	ND	ND	ND	ND
Molybdenum	ND	ND	ND	ND	ND
Naphthalene	0.01	8.60E-07	4.40E-07	1.30E-04	1.30E-04
Nickel	ND	ND	ND	ND	ND
Phenol	0.01	2.30E-08	9.10E-09	2.50E-11	3.20E-08
Pyrene	0.64	2.90E-05	1.50E-05	ND	4.40E-05
Tin	ND	ND	ND	ND	ND
Zinc	ND	ND	ND	ND	ND
Totals		1.70E-03	8.50E-04	3.00E-04	2.80E-03

Table S60. Sector no. 51 – cancer risk – aggregate residents

Substance	Content (mg/kg)	CRo	CRd	CRinh	CR
Arsenic	ND	ND	ND	ND	ND
Benzo(a)anthracene	0.33	2.20E-07	8.50E-08	9.90E-09	3.20E-07
Benzo(a)pyrene	0.35	2.30E-06	8.90E-07	2.30E-10	3.20E-06
Benzo(b)fluoranthene	0.51	3.40E-07	1.30E-07	3.30E-11	4.70E-07
Benzo(k)fluoranthene	0.19	1.20E-08	4.80E-09	1.20E-12	1.70E-08
Cadmium	ND	ND	ND	ND	ND
Chromium (VI)	ND	ND	ND	ND	ND
Chrysene	0.39	2.60E-09	1.00E-09	2.60E-13	3.60E-09
Cobalt	ND	ND	ND	ND	ND
Dibenzo(a,h)anthracene	0.06	4.20E-07	1.60E-07	4.00E-11	5.80E-07
Indeno(1,2,3-cd)pyrene	0.27	1.80E-07	7.00E-08	1.80E-11	2.50E-07
Naphthalene	0.01	ND	ND	6.70E-09	6.70E-09
Nickel	ND	ND	ND	ND	ND
Totals		3.50E-06	1.30E-06	1.70E-08	4.80E-06

Table S61. Backyard K02 – non-cancer risk – children

Substance	Content (mg/kg)	HQo	HQd	HQinh	HI
Acenaphtene	0.01	2.70E-06	9.70E-07	ND	3.60E-06
Anthracene	0.03	1.40E-06	5.30E-07	ND	2.00E-06
Arsenic	ND	ND	ND	ND	ND
Barium	ND	ND	ND	ND	ND
Benzo(a)pyrene	0.26	1.10E-02	4.00E-03	7.10E-05	1.50E-02
Cadmium	ND	ND	ND	ND	ND
Chromium (III)	ND	ND	ND	ND	ND
Chromium (VI)	ND	ND	ND	ND	ND
Cobalt	ND	ND	ND	ND	ND
Copper	ND	ND	ND	ND	ND
Fluoranthene	0.46	1.50E-04	5.40E-05	ND	2.00E-04
Fluorene	0.01	4.00E-06	1.50E-06	ND	5.40E-06
Mercury	ND	ND	ND	ND	ND
Molybdenum	ND	ND	ND	ND	ND
Naphthalene	0.01	8.00E-06	2.90E-06	1.40E-04	1.50E-04
Nickel	ND	ND	ND	ND	ND
Phenol	0.01	2.10E-07	6.00E-08	1.40E-11	2.70E-07
Pyrene	0.40	1.70E-04	6.10E-05	ND	2.30E-04
Tin	ND	ND	ND	ND	ND
Zinc	ND	ND	ND	ND	ND
Totals		1.10E-02	4.10E-03	2.10E-04	1.60E-02

Table S62. Backyard K02 – non-cancer risk – adults

Substance	Content (mg/kg)	HQo	HQd	HQinh	HI
Acenaphtene	0.01	2.90E-07	1.50E-07	ND	4.30E-07
Anthracene	0.03	1.60E-07	8.10E-08	ND	2.40E-07
Arsenic	ND	ND	ND	ND	ND
Barium	ND	ND	ND	ND	ND
Benzo(a)pyrene	0.26	1.20E-03	6.10E-04	7.10E-05	1.90E-03
Cadmium	ND	ND	ND	ND	ND
Chromium (III)	ND	ND	ND	ND	ND
Chromium (VI)	ND	ND	ND	ND	ND
Cobalt	ND	ND	ND	ND	ND
Copper	ND	ND	ND	ND	ND
Fluoranthene	0.46	1.60E-05	8.20E-06	ND	2.40E-05
Fluorene	0.01	4.30E-07	2.20E-07	ND	6.50E-07
Mercury	ND	ND	ND	ND	ND
Molybdenum	ND	ND	ND	ND	ND
Naphthalene	0.01	8.60E-07	4.40E-07	7.00E-05	7.10E-05
Nickel	ND	ND	ND	ND	ND
Phenol	0.01	2.30E-08	9.10E-09	1.40E-11	3.20E-08
Pyrene	0.40	1.80E-05	9.40E-06	ND	2.70E-05
Tin	ND	ND	ND	ND	ND
Zinc	ND	ND	ND	ND	ND
Totals		1.20E-03	6.30E-04	1.40E-04	2.00E-03

Table S63. Backyard K02 – cancer risk – aggregate residents

Substance	Content (mg/kg)	CRo	CRd	CRinh	CR
Arsenic	ND	ND	ND	ND	ND
Benzo(a)anthracene	0.24	1.60E-07	6.20E-08	3.90E-09	2.30E-07
Benzo(a)pyrene	0.26	1.70E-06	6.70E-07	9.20E-11	2.40E-06
Benzo(b)fluoranthene	0.38	2.60E-07	9.90E-08	1.40E-11	3.60E-07
Benzo(k)fluoranthene	0.15	9.80E-09	3.80E-09	5.20E-13	1.40E-08
Cadmium	ND	ND	ND	ND	ND
Chromium (VI)	ND	ND	ND	ND	ND
Chrysene	0.25	1.70E-09	6.40E-10	8.80E-14	2.30E-09
Cobalt	ND	ND	ND	ND	ND
Dibenzo(a,h)anthracene	0.01	8.40E-08	3.20E-08	4.40E-12	1.20E-07
Indeno(1,2,3-cd)pyrene	0.24	1.60E-07	6.20E-08	8.60E-12	2.20E-07
Naphthalene	0.01	ND	ND	3.70E-09	3.70E-09
Nickel	ND	ND	ND	ND	ND
Totals		2.40E-06	9.30E-07	7.70E-09	3.40E-06

Table S64. Backyard K11 – non-cancer risk – children

Substance	Content (mg/kg)	HQo	HQd	HQinh	HI
Acenaphtene	0.01	2.70E-06	9.70E-07	ND	3.60E-06
Anthracene	0.03	1.20E-06	4.20E-07	ND	1.60E-06
Arsenic	ND	ND	ND	ND	ND
Barium	ND	ND	ND	ND	ND
Benzo(a)pyrene	0.26	1.10E-02	4.00E-03	9.50E-05	1.50E-02
Cadmium	ND	ND	ND	ND	ND
Chromium (III)	ND	ND	ND	ND	ND
Chromium (VI)	ND	ND	ND	ND	ND
Cobalt	ND	ND	ND	ND	ND
Copper	ND	ND	ND	ND	ND
Fluoranthene	0.31	9.80E-05	3.60E-05	ND	1.30E-04
Fluorene	0.01	4.00E-06	1.50E-06	ND	5.40E-06
Mercury	ND	ND	ND	ND	ND
Molybdenum	ND	ND	ND	ND	ND
Naphthalene	0.01	8.00E-06	2.90E-06	1.90E-04	2.00E-04
Nickel	ND	ND	ND	ND	ND
Phenol	0.01	2.10E-07	6.00E-08	1.80E-11	2.70E-07
Pyrene	0.27	1.10E-04	4.10E-05	ND	1.60E-04
Tin	ND	ND	ND	ND	ND
Zinc	ND	ND	ND	ND	ND
Totals		1.10E-02	4.10E-03	2.90E-04	1.60E-02

Table S65. Backyard K11 – non-cancer risk – adults

Substance	Content (mg/kg)	HQo	HQd	HQinh	HI
Acenaphtene	0.01	2.90E-07	1.50E-07	ND	4.30E-07
Anthracene	0.03	1.20E-07	6.40E-08	ND	1.90E-07
Arsenic	ND	ND	ND	ND	ND
Barium	ND	ND	ND	ND	ND
Benzo(a)pyrene	0.26	1.20E-03	6.10E-04	9.50E-05	1.90E-03
Cadmium	ND	ND	ND	ND	ND
Chromium (III)	ND	ND	ND	ND	ND
Chromium (VI)	ND	ND	ND	ND	ND
Cobalt	ND	ND	ND	ND	ND
Copper	ND	ND	ND	ND	ND
Fluoranthene	0.31	1.00E-05	5.40E-06	ND	1.60E-05
Fluorene	0.01	4.30E-07	2.20E-07	ND	6.50E-07
Mercury	ND	ND	ND	ND	ND
Molybdenum	ND	ND	ND	ND	ND
Naphthalene	0.01	8.60E-07	4.40E-07	9.40E-05	9.50E-05
Nickel	ND	ND	ND	ND	ND
Phenol	0.01	2.30E-08	9.10E-09	1.80E-11	3.20E-08
Pyrene	0.27	1.20E-05	6.30E-06	ND	1.90E-05
Tin	ND	ND	ND	ND	ND
Zinc	ND	ND	ND	ND	ND
Totals		1.20E-03	6.20E-04	1.90E-04	2.00E-03

Table S66. Backyard K11 – cancer risk – aggregate residents

Substance	Content (mg/kg)	CRo	CRd	CRinh	CR
Arsenic	ND	ND	ND	ND	ND
Benzo(a)anthracene	0.20	1.30E-07	5.10E-08	4.30E-09	1.90E-07
Benzo(a)pyrene	0.26	1.70E-06	6.60E-07	1.20E-10	2.40E-06
Benzo(b)fluoranthene	0.36	2.40E-07	9.20E-08	1.70E-11	3.30E-07
Benzo(k)fluoranthene	0.14	9.30E-09	3.60E-09	6.60E-13	1.30E-08
Cadmium	ND	ND	ND	ND	ND
Chromium (VI)	ND	ND	ND	ND	ND
Chrysene	0.21	1.40E-09	5.30E-10	9.80E-14	1.90E-09
Cobalt	0.00	ND	ND	ND	ND
Dibenzo(a,h)anthracene	0.01	8.40E-08	3.20E-08	6.00E-12	1.20E-07
Indeno(1,2,3-cd)pyrene	0.24	1.60E-07	6.10E-08	1.10E-11	2.20E-07
Naphthalene	0.01	ND	ND	4.90E-09	4.90E-09
Nickel	ND	ND	ND	ND	ND
Totals		2.30E-06	9.00E-07	9.40E-09	3.30E-06

Table S67. Backyard K26 – non-cancer risk – children

Substance	Content (mg/kg)	HQo	HQd	HQinh	HI
Acenaphtene	0.01	2.70E-06	9.70E-07	ND	3.60E-06
Anthracene	0.01	5.30E-07	1.90E-07	ND	7.30E-07
Arsenic	ND	ND	ND	ND	ND
Barium	ND	ND	ND	ND	ND
Benzo(a)pyrene	0.08	3.50E-03	1.30E-03	2.90E-05	4.80E-03
Cadmium	ND	ND	ND	ND	ND
Chromium (III)	ND	ND	ND	ND	ND
Chromium (VI)	ND	ND	ND	ND	ND
Cobalt	ND	ND	ND	ND	ND
Copper	ND	ND	ND	ND	ND
Fluoranthene	0.12	3.90E-05	1.40E-05	ND	5.30E-05
Fluorene	0.01	4.00E-06	1.50E-06	ND	5.40E-06
Mercury	ND	ND	ND	ND	ND
Molybdenum	ND	ND	ND	ND	ND
Naphthalene	0.01	8.00E-06	2.90E-06	1.80E-04	1.90E-04
Nickel	ND	ND	ND	ND	ND
Phenol	0.01	2.10E-07	6.00E-08	1.80E-11	2.70E-07
Pyrene	0.10	4.40E-05	1.60E-05	ND	6.00E-05
Tin	ND	ND	ND	ND	ND
Zinc	ND	ND	ND	ND	ND
Totals		3.60E-03	1.30E-03	2.10E-04	5.10E-03

Table S68. Backyard K26 – non-cancer risk – adults

Substance	Content (mg/kg)	HQo	HQd	HQinh	HI
Acenaphtene	0.01	2.90E-07	1.50E-07	ND	4.30E-07
Anthracene	0.01	5.70E-08	3.00E-08	ND	8.70E-08
Arsenic	ND	ND	ND	ND	ND
Barium	ND	ND	ND	ND	ND
Benzo(a)pyrene	0.08	3.70E-04	1.90E-04	2.90E-05	6.00E-04
Cadmium	ND	ND	ND	ND	ND
Chromium (III)	ND	ND	ND	ND	ND
Chromium (VI)	ND	ND	ND	ND	ND
Cobalt	ND	ND	ND	ND	ND
Copper	ND	ND	ND	ND	ND
Fluoranthene	0.12	4.10E-06	2.10E-06	ND	6.30E-06
Fluorene	0.01	4.30E-07	2.20E-07	ND	6.50E-07
Mercury	ND	ND	ND	ND	ND
Molybdenum	ND	ND	ND	ND	ND
Naphthalene	0.01	8.60E-07	4.40E-07	9.00E-05	9.20E-05
Nickel	ND	ND	ND	ND	ND
Phenol	0.01	2.30E-08	9.10E-09	1.80E-11	3.20E-08
Pyrene	0.10	4.70E-06	2.50E-06	ND	7.20E-06
Tin	ND	ND	ND	ND	ND
Zinc	ND	ND	ND	ND	ND
Totals		3.80E-04	2.00E-04	1.20E-04	7.10E-04

Table S69. Backyard K26 – cancer risk – aggregate residents

Substance	Content (mg/kg)	CRo	CRd	CRinh	CR
Arsenic	ND	ND	ND	ND	ND
Benzo(a)anthracene	0.07	4.60E-08	1.80E-08	1.40E-09	6.50E-08
Benzo(a)pyrene	0.08	5.50E-07	2.10E-07	3.80E-11	7.60E-07
Benzo(b)fluoranthene	0.14	9.10E-08	3.50E-08	6.30E-12	1.30E-07
Benzo(k)fluoranthene	0.04	2.50E-09	9.80E-10	1.80E-13	3.50E-09
Cadmium	ND	ND	ND	ND	ND
Chromium (VI)	ND	ND	ND	ND	ND
Chrysene	0.07	4.90E-10	1.90E-10	3.40E-14	6.80E-10
Cobalt	ND	ND	ND	ND	ND
Dibenzo(a,h)anthracene	0.01	8.40E-08	3.20E-08	5.70E-12	1.20E-07
Indeno(1,2,3-cd)pyrene	0.07	4.80E-08	1.90E-08	3.30E-12	6.70E-08
Naphthalene	0.01	ND	ND	4.70E-09	4.70E-09
Nickel	ND	ND	ND	ND	ND
Totals		8.20E-07	3.20E-07	6.20E-09	1.20E-06

Table S70. Backyard K30 – non-cancer risk – children

Substance	Content (mg/kg)	HQo	HQd	HQinh	HI
Acenaphtene	0.04	9.20E-06	3.30E-06	ND	1.20E-05
Anthracene	0.20	8.40E-06	3.10E-06	ND	1.10E-05
Arsenic	3.90	1.00E-01	1.40E-02	1.60E-04	1.20E-01
Barium	130.00	8.00E-03	3.20E-03	1.50E-04	1.10E-02
Benzo(a)pyrene	1.30	5.70E-02	2.10E-02	4.00E-04	7.80E-02
Chromium (III)	9.70	8.30E-05	1.80E-04	ND	2.60E-04
Chromium (VI)	1.60	6.90E-03	7.70E-03	9.60E-06	1.50E-02
Cobalt	3.30	1.40E-01	3.90E-03	3.30E-04	1.40E-01
Copper	16.00	5.20E-03	1.40E-04	ND	5.30E-03
Fluoranthene	1.80	5.70E-04	2.10E-04	ND	7.80E-04
Fluorene	0.04	1.20E-05	4.50E-06	ND	1.70E-05
Mercury	0.12	5.30E-03	2.10E-03	2.50E-07	7.40E-03
Naphthalene	0.01	8.00E-06	2.90E-06	1.50E-04	1.60E-04
Nickel	10.00	6.60E-03	4.70E-03	6.90E-05	1.10E-02
Phenol	0.01	2.10E-07	6.00E-08	1.50E-11	2.70E-07
Pyrene	1.60	7.00E-04	2.50E-04	ND	9.50E-04
Tin	1.40	3.00E-05	8.30E-07	ND	3.00E-05
Zinc	160.00	6.90E-03	1.90E-04	ND	7.10E-03
Totals		3.40E-01	5.80E-02	1.30E-03	4.00E-01

Table S71. Backyard K30 – non-cancer risk – adults

Substance	Content (mg/kg)	HQo	HQd	HQinh	HI
Acenaphtene	0.04	9.80E-07	5.10E-07	ND	1.50E-06
Anthracene	0.20	9.00E-07	4.70E-07	ND	1.40E-06
Arsenic	3.90	1.10E-02	2.20E-03	1.60E-04	1.30E-02
Barium	130.00	8.60E-04	4.90E-04	1.50E-04	1.50E-03
Benzo(a)pyrene	1.30	6.10E-03	3.20E-03	4.00E-04	9.70E-03
Chromium (III)	9.70	8.80E-06	2.70E-05	ND	3.60E-05
Chromium (VI)	1.60	7.40E-04	1.20E-03	9.60E-06	1.90E-03
Cobalt	3.30	1.50E-02	6.00E-04	3.30E-04	1.60E-02
Copper	16.00	5.50E-04	2.20E-05	ND	5.80E-04
Fluoranthene	1.80	6.20E-05	3.20E-05	ND	9.30E-05
Fluorene	0.04	1.30E-06	6.90E-07	ND	2.00E-06
Mercury	0.12	5.70E-04	3.20E-04	2.50E-07	8.90E-04
Naphthalene	0.01	8.60E-07	4.40E-07	7.70E-05	7.80E-05
Nickel	10.00	7.10E-04	7.10E-04	6.90E-05	1.50E-03
Phenol	0.01	2.30E-08	9.10E-09	1.50E-11	3.20E-08
Pyrene	1.60	7.50E-05	3.90E-05	ND	1.10E-04
Tin	1.40	3.20E-06	1.30E-07	ND	3.30E-06
Zinc	160.00	7.40E-04	3.00E-05	ND	7.70E-04
Totals		3.70E-02	8.90E-03	1.20E-03	4.60E-02

Table S72. Backyard K30 – cancer risk – aggregate residents

Substance	Content (mg/kg)	CRo	CRd	CRinh	CR
Arsenic	3.90	5.60E-06	8.80E-07	4.40E-09	6.40E-06
Benzo(a)anthracene	0.72	4.80E-07	1.90E-07	1.30E-08	6.80E-07
Benzo(a)pyrene	1.30	9.00E-06	3.40E-06	5.20E-10	1.20E-05
Benzo(b)fluoranthene	1.90	1.30E-06	4.80E-07	7.30E-11	1.70E-06
Benzo(k)fluoranthene	0.61	4.10E-08	1.60E-08	2.40E-12	5.60E-08
Chromium (VI)	1.60	5.40E-06	6.40E-06	8.80E-08	1.20E-05
Chrysene	1.20	7.70E-09	3.00E-09	4.50E-13	1.10E-08
Cobalt	3.30	ND	ND	7.60E-09	7.60E-09
Dibenzo(a,h)anthracene	0.01	8.40E-08	3.20E-08	4.90E-12	1.20E-07
Indeno(1,2,3-cd)pyrene	1.10	7.50E-07	2.90E-07	4.40E-11	1.00E-06
Naphthalene	0.01	ND	ND	4.00E-09	4.00E-09
Nickel	10.00	ND	ND	6.90E-10	6.90E-10
Totals		2.30E-05	1.20E-05	1.20E-07	3.40E-05

Table S73. Backyard K31 – non-cancer risk – children

Substance	Content (mg/kg)	HQo	HQd	HQinh	HI
Acenaphtene	0.01	2.70E-06	9.70E-07	ND	3.60E-06
Anthracene	0.05	2.20E-06	7.90E-07	ND	3.00E-06
Arsenic	3.30	8.50E-02	1.20E-02	1.50E-04	9.70E-02
Barium	75.00	4.80E-03	1.90E-03	1.00E-04	6.80E-03
Benzo(a)pyrene	0.27	1.10E-02	4.20E-03	9.30E-05	1.60E-02
Chromium (III)	6.40	5.40E-05	1.20E-04	ND	1.70E-04
Chromium (VI)	1.10	4.50E-03	5.10E-03	7.40E-06	9.60E-03
Cobalt	2.30	9.80E-02	2.70E-03	2.70E-04	1.00E-01
Copper	9.40	3.00E-03	8.40E-05	ND	3.10E-03
Fluoranthene	0.48	1.50E-04	5.50E-05	ND	2.10E-04
Fluorene	0.01	4.00E-06	1.50E-06	ND	5.40E-06
Mercury	0.05	2.10E-03	8.50E-04	1.20E-07	3.00E-03
Naphthalene	0.01	8.00E-06	2.90E-06	1.80E-04	1.90E-04
Nickel	6.90	4.40E-03	3.10E-03	5.30E-05	7.50E-03
Phenol	0.01	2.10E-07	6.00E-08	1.70E-11	2.70E-07
Pyrene	0.42	1.80E-04	6.40E-05	ND	2.40E-04
Tin	0.50	1.10E-05	3.00E-07	ND	1.10E-05
Zinc	59.00	2.50E-03	7.00E-05	ND	2.60E-03
Totals		2.20E-01	3.10E-02	8.70E-04	2.50E-01

Table S74. Backyard K31 – non-cancer risk – adults

Substance	Content (mg/kg)	HQo	HQd	HQinh	HI
Acenaphtene	0.01	2.90E-07	1.50E-07	ND	4.30E-07
Anthracene	0.05	2.30E-07	1.20E-07	ND	3.50E-07
Arsenic	3.30	9.10E-03	1.80E-03	1.50E-04	1.10E-02
Barium	75.00	5.20E-04	2.90E-04	1.00E-04	9.10E-04
Benzo(a)pyrene	0.27	1.20E-03	6.40E-04	9.30E-05	2.00E-03
Chromium (III)	6.40	5.80E-06	1.80E-05	ND	2.40E-05
Chromium (VI)	1.10	4.80E-04	7.70E-04	7.40E-06	1.30E-03
Cobalt	2.30	1.10E-02	4.20E-04	2.70E-04	1.10E-02
Copper	9.40	3.20E-04	1.30E-05	ND	3.30E-04
Fluoranthene	0.48	1.60E-05	8.50E-06	ND	2.50E-05
Fluorene	0.01	4.30E-07	2.20E-07	ND	6.50E-07
Mercury	0.05	2.30E-04	1.30E-04	1.20E-07	3.60E-04
Naphthalene	0.01	8.60E-07	4.40E-07	8.90E-05	9.00E-05
Nickel	6.90	4.70E-04	4.70E-04	5.30E-05	9.90E-04
Phenol	0.01	2.30E-08	9.10E-09	1.70E-11	3.20E-08
Pyrene	0.42	1.90E-05	9.80E-06	ND	2.90E-05
Tin	0.50	1.10E-06	4.60E-08	ND	1.20E-06
Zinc	59.00	2.70E-04	1.10E-05	ND	2.80E-04
Totals		2.40E-02	4.60E-03	7.80E-04	2.80E-02

Table S75. Backyard K31 – cancer risk – aggregate residents

Substance	Content (mg/kg)	CRo	CRd	CRinh	CR
Arsenic	3.30	4.70E-06	7.40E-07	4.20E-09	5.40E-06
Benzo(a)anthracene	0.26	1.70E-07	6.60E-08	5.30E-09	2.40E-07
Benzo(a)pyrene	0.27	1.80E-06	6.90E-07	1.20E-10	2.50E-06
Benzo(b)fluoranthene	0.37	2.50E-07	9.60E-08	1.70E-11	3.50E-07
Benzo(k)fluoranthene	0.14	9.30E-09	3.60E-09	6.30E-13	1.30E-08
Chromium (VI)	1.10	3.60E-06	4.20E-06	6.70E-08	7.80E-06
Chrysene	0.16	1.00E-09	4.00E-10	7.00E-14	1.40E-09
Cobalt	2.30	ND	ND	6.20E-09	6.20E-09
Dibenzo(a,h)anthracene	0.01	8.40E-08	3.20E-08	5.70E-12	1.20E-07
Indeno(1,2,3-cd)pyrene	0.23	1.60E-07	6.00E-08	1.10E-11	2.20E-07
Naphthalene	0.01	ND	ND	4.70E-09	4.70E-09
Nickel	6.90	ND	ND	5.30E-10	5.30E-10
Totals		1.10E-05	5.90E-06	8.80E-08	1.70E-05

Table S76. Backyard K33 – non-cancer risk – children

Substance	Content (mg/kg)	HQo	HQd	HQinh	HI
Acenaphtene	0.01	2.70E-06	9.70E-07	ND	3.60E-06
Anthracene	0.04	1.60E-06	5.90E-07	ND	2.20E-06
Arsenic	2.50	6.50E-02	9.10E-03	1.00E-04	7.40E-02
Barium	46.00	3.00E-03	1.20E-03	5.70E-05	4.20E-03
Benzo(a)pyrene	0.20	8.40E-03	3.00E-03	6.00E-05	1.10E-02
Chromium (III)	4.50	3.80E-05	8.30E-05	ND	1.20E-04
Chromium (VI)	0.75	3.20E-03	3.60E-03	4.60E-06	6.80E-03
Cobalt	1.90	7.90E-02	2.20E-03	1.90E-04	8.10E-02
Copper	18.00	5.80E-03	1.60E-04	ND	5.90E-03
Fluoranthene	0.33	1.10E-04	3.90E-05	ND	1.40E-04
Fluorene	0.01	4.00E-06	1.50E-06	ND	5.40E-06
Mercury	0.05	2.10E-03	8.50E-04	1.00E-07	3.00E-03
Naphthalene	0.01	8.00E-06	2.90E-06	1.60E-04	1.70E-04
Nickel	4.60	2.90E-03	2.10E-03	3.10E-05	5.00E-03
Phenol	0.01	2.10E-07	6.00E-08	1.50E-11	2.70E-07
Pyrene	0.28	1.20E-04	4.40E-05	ND	1.60E-04
Tin	1.40	3.10E-05	8.60E-07	ND	3.20E-05
Zinc	73.00	3.10E-03	8.70E-05	ND	3.20E-03
Totals		1.80E-01	2.30E-02	6.20E-04	1.90E-01

Table S77. Backyard K33 – non-cancer risk – adults

Substance	Content (mg/kg)	HQo	HQd	HQinh	HI
Acenaphtene	0.01	2.90E-07	1.50E-07	ND	4.30E-07
Anthracene	0.04	1.70E-07	9.00E-08	ND	2.60E-07
Arsenic	2.50	6.90E-03	1.40E-03	1.00E-04	8.40E-03
Barium	46.00	3.20E-04	1.80E-04	5.70E-05	5.50E-04
Benzo(a)pyrene	0.20	8.90E-04	4.60E-04	6.00E-05	1.40E-03
Chromium (III)	4.50	4.10E-06	1.30E-05	ND	1.70E-05
Chromium (VI)	0.75	3.40E-04	5.50E-04	4.60E-06	8.90E-04
Cobalt	1.90	8.40E-03	3.40E-04	1.90E-04	9.00E-03
Copper	18.00	6.20E-04	2.50E-05	ND	6.40E-04
Fluoranthene	0.33	1.10E-05	5.90E-06	ND	1.70E-05
Fluorene	0.01	4.30E-07	2.20E-07	ND	6.50E-07
Mercury	0.05	2.30E-04	1.30E-04	1.00E-07	3.60E-04
Naphthalene	0.01	8.60E-07	4.40E-07	7.90E-05	8.00E-05
Nickel	4.60	3.20E-04	3.10E-04	3.10E-05	6.60E-04
Phenol	0.01	2.30E-08	9.10E-09	1.50E-11	3.20E-08
Pyrene	0.28	1.30E-05	6.70E-06	ND	2.00E-05
Tin	1.40	3.30E-06	1.30E-07	ND	3.40E-06
Zinc	73.00	3.30E-04	1.30E-05	ND	3.50E-04
Totals		1.90E-02	3.50E-03	5.40E-04	2.20E-02

Table S78. Backyard K33 – cancer risk – aggregate residents

Substance	Content (mg/kg)	CRo	CRd	CRinh	CR
Arsenic	2.50	3.60E-06	5.60E-07	2.90E-09	4.10E-06
Benzo(a)anthracene	0.18	1.20E-07	4.60E-08	3.30E-09	1.70E-07
Benzo(a)pyrene	0.20	1.30E-06	5.00E-07	7.90E-11	1.80E-06
Benzo(b)fluoranthene	0.26	1.70E-07	6.60E-08	1.00E-11	2.40E-07
Benzo(k)fluoranthene	0.10	6.40E-09	2.40E-09	3.80E-13	8.80E-09
Chromium (VI)	0.75	2.50E-06	3.00E-06	4.20E-08	5.50E-06
Chrysene	0.17	1.10E-09	4.30E-10	6.70E-14	1.60E-09
Cobalt	1.90	ND	ND	4.40E-09	4.40E-09
Dibenzo(a,h)anthracene	0.01	8.40E-08	3.20E-08	5.00E-12	1.20E-07
Indeno(1,2,3-cd)pyrene	0.17	1.10E-07	4.20E-08	6.60E-12	1.50E-07
Naphthalene	0.01	ND	ND	4.10E-09	4.10E-09
Nickel	4.60	ND	ND	3.20E-10	3.20E-10
Totals		7.90E-06	4.20E-06	5.70E-08	1.2E-05
